# Microbial communities of aquatic environments on Heard Island characterized by pyrotag sequencing and environmental data

**DOI:** 10.1038/srep44480

**Published:** 2017-03-14

**Authors:** Michelle A. Allen, Ricardo Cavicchioli

**Affiliations:** 1 School of Biotechnology and Biomolecular Sciences, The University of New South Wales, Sydney, New South Wales, 2052, Australia

## Abstract

Heard Island in the Southern Ocean is a biological hotspot that is suffering the effects of climate change. Significant glacier retreat has generated proglacial lagoons, some of which are open to the ocean. We used pyrotag sequencing of SSU rRNA genes and environmental data to characterize microorganisms from two pools adjacent to animal breeding areas, two glacial lagoons and Atlas Cove (marine site). The more abundant taxa included *Actinobacteria, Bacteroidetes* and *Proteobacteria*, ciliates and picoflagellates (e.g. *Micromonas*), and relatively few *Archaea*. Seal Pool, which is rich in organic matter, was characterized by a heterotrophic degradative community, while the less eutrophic Atlas Pool had more eucaryotic primary producers. Brown Lagoon, with the lowest nutrient levels, had *Eucarya* and *Bacteria* predicted to be oligotrophs, possess small cell sizes, and have the ability to metabolize organic matter. The marine influence on Winston Lagoon was evident by its salinity and the abundance of marine-like *Gammaproteobacteria*, while also lacking typical marine eucaryotes indicating the system was still functioning as a distinct niche. This is the first microbiology study of Heard Island and revealed that communities are distinct at each location and heavily influenced by local environmental factors.

Heard Island (53°05′S, 73°30′E) is a 367 km^2^ volcanic island located ~4350 km southwest of Western Australia on the Kerguelen Plateau^1^. Surrounded by cold Antarctic waters and located south of the Antarctic Polar Front, it experiences strong and persistent westerly winds, low seasonal and daily temperature ranges, and high precipitation^2^. The vast bulk of the island is covered in glaciers, which radiate from the summit of the intermittently active volcanic peak Big Ben (2750 m above sea level) to the tidewater (those with northerly to southwesterly aspects) or terminate on land or in lagoons inland from the ocean (those with southerly to northeasterly aspects)^3^ (Fig. 1). Isolated vegetated headlands emerge between the glaciers, and together with the exposed plains and shoreline, are colonized by very high numbers of breeding and non-breeding mammals and seabirds^4^,^5^,^6^. Coastal eutrophication due to input from penguins, other seabirds and seals, combined with long daylight hours in summer, can lead to high primary production^4^,^7^. In contrast to the studies of the geology, climate, vegetation and wildlife (invertebrates, fish, marine mammals and birds) of Heard Island^8^, and despite the possible presence of microorganisms in lava tube caves^9^ and the potential of microorganisms to cause disease on the island^10^, no studies characterizing microorganisms from this World-Heritage-listed site have been reported.

Consistent with an almost 1 °C rise in the average annual air temperature of Heard Island from 1948–1954 to 1997–2001^11^, total glacier coverage has decreased from 288 km^2^ in 1947 (79% coverage) to 257 km^2^ in 1988 (70%) and 231 km^2^ in 2008 (63%)^12^,^13^ (Fig. 1a). While all glaciers have retreated, individual glaciers to the east of Big Ben have been particularly affected and closely monitored, both during the infrequent research expeditions to the island and via satellite. In 1947 Winston Glacier had already retreated from the shoreline, and continued to retreat a further 1.6 km by 1963^14^. It then advanced during the 1970s, only to retreat again in the 1980s^12^. The Brown and Stephenson glaciers began to retreat in the 1960s^14^, with proglacial lagoons developing by the mid-1980s. The terminus of Brown Glacier retreated at an average rate of 30 m per year between 1947 and 2004 to reach a total of 1.2 km from the coast, resulting in a loss of 29% of its original area, which is a remarkable change given its 25 m high terminal ice cliffs were calving into the ocean in 1947^3^. Between January 2004 and January 2006, Brown Glacier retreated <0.1 km and Winston Glacier retreated by ~0.2 km^13^,^15^. Based on data from 1947 and 2008, ~55 km^2^ or 15% of the island’s terrain has been exposed by the retreating ice, revealing or expanding several large proglacial lagoons and making newly deglaciated land available for colonization. Together with higher ambient temperatures and increased availability of liquid water (more precipitation as rain rather than snow), animal, plant and microbial communities will be able to expand into new niches. The effect of these changes on terrestrial and lacustrine food webs will be important to establish and monitor.

Compared to other Sub-Antarctic islands which experience tourism or more regular research visits, Heard Island is largely pristine and remarkably free of invasive species^16^. However, climate change and warming conditions increase the potential for non-native species to become established. Moreover, the Kerguelen Plateau is the largest volcanic plateau in the Southern Ocean and provides a major source of iron that stimulates phytoplankton blooms around, and to its east^1^. Climate-related changes have implications for the marine environment surrounding Heard Island, as increased freshwater from melting glaciers will carry iron and nitrogen rich minerals, as well as microbial populations with as yet uncharacterized roles in C, N and P cycling, out into the sea and eastward along the flow of the Antarctic Circumpolar Current.

In order to begin the characterization of microbial communities present in pools and lagoons on Heard Island, pyrotag sequencing of SSU rRNA genes was performed on biomass from water samples collected in December 2008 from two proglacial lagoons (Brown Lagoon and Winston Lagoon), two pools formed from rainwater and snowmelt adjacent to seal and penguin breeding areas on the Azorella Peninsula, and seawater from Atlas Cove (Fig. 1). The biomass was captured by sequential fractionation through a 20 μm prefilter onto 3.0-, 0.8-, and 0.1-μm filters using an approach previously adopted for Southern Ocean^17^ and Antarctic lake systems^18^ that facilitates taxonomic analyses by reducing the overall complexity and enabling size partitioning of samples^19^,^20^. By combining environmental with pyrotag sequencing data, we were able to infer important functional processes and the environmental factors affecting communities and ecosystem function. This snapshot provides the first insight into microbial community diversity and richness in aquatic systems on Heard Island, and provides a baseline for ongoing monitoring of this unique environment that is subject to significant environmental change.

## Results

### Water composition of each location

**Figure 1:**
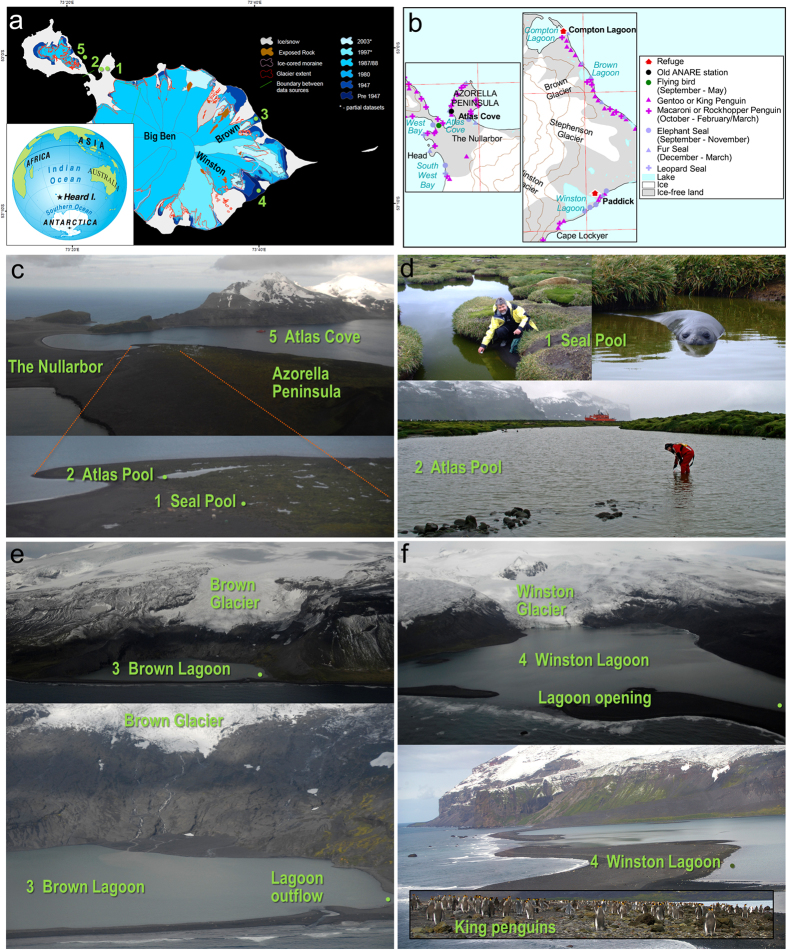
Heard Island sampling sites. (**a**) Map showing change in glacier boundaries from pre 1947 to 2006–2009 (red outline), with sample collection sites numbered: 1, Seal Pool; 2, Atlas Pool; 3, Brown Lagoon; 4, Winston Lagoon; 5, Atlas Cove. Modified from a map courtesy of the Australian Antarctic Data Centre, CAASM metadata record ID Heard_Island_digitising_2009, map number 13691, © Commonwealth of Australia 2009. Inset: Depiction of Earth showing the isolation of Heard Island relative to surrounding continents with the closest being Antarctica (1,700 km to Davis Station), Australia (4,100 km to Perth) and Africa (4,800 to Johannesburg). Image courtesy of the Australian Geographic Cartographic Division. (**b**) Distribution of wildlife near the sample collection sites. Sections of a map courtesy of the Australian Antarctic Data Centre, map number 12583, © Commonwealth of Australia 2000. (**c**) Seal Pool (1) and Atlas Pool (2) on the Azorella Peninsula relative to the Atlas Cove (5) location where seawater was collected from the *Aurora Australis* (red ship). Photographer Gary Miller. (**d**) Sample collection at Seal Pool with elephant seal in Seal Pool prior to sampling, and Atlas Pool with *Aurora Australis* in the background. Photographers the Australian Antarctic Division and Rick Cavicchioli. (**e**) Aerial views of Brown Glacier above Brown Lagoon showing the rocky sill separating the lagoon from the ocean, and the lagoon outflow and approximate sampling sites. Photographer Gary Miller. (**f**) Aerial views of Winston Glacier and Winston Lagoon showing the glacier tongue in contact with the lagoon, and the opening of the lagoon permitting influx from the ocean, the approximate sampling site, and king penguins (inset) on the ocean-side shore of the lagoon at the time of sampling. Photographer Gary Miller and Stephen Brown.

Seal Pool and Atlas Pool were pools designated in this study (see Methods and Fig. 1). Chemical analysis revealed a great deal of variation among the five aquatic locations (Table 1). All waters were brackish, with Seal Pool the closest to freshwater, followed by Atlas Pool, and then Brown Lagoon with conductivity of 6580 μS cm^−1^. The conductivity of Winston Lagoon indicates it was ~72% as saline as typical seawater (taken as 55000 μS cm^−1^; ref. 21), with the conductivity of the Atlas Cove site being 88% of seawater salinity, which may reflect the input of glacial meltwater from Heard Island. Sulfur levels increased with increasing salinity. Brown Lagoon had the lowest phosphorus levels, with all samples containing ≥27 μg P L^−1^. Nitrogen compounds were most prevalent in Seal and Atlas pools, with highest levels of ammonia (24.9 mg N L^−1^) in Seal Pool and nitrate in Atlas Pool (4.32 mg N L^−1^). Brown Lagoon had the lowest levels of ammonia, nitrate, nitrite and total nitrogen, even though it had a moderate level of dissolved organic carbon (DOC) (27 mg L^−1^). Seal Pool had the highest DOC (170 mg L^−1^).

### Microbial diversity

The biomass captured on each filter from each location (total of 15) is referred to as a sample. After quality control processing in QIIME^22^, a total of 198709 SSU rRNA gene pyrotag sequences (length 222–538 bp, mean 379 bp) were retained from the initial 209304 denoised, chimera checked and trimmed sequences obtained from the 15 samples. As pyrotags of length 250–400 bp have been shown to be adequate for determining accurate community diversity^23^,^24^ and taxonomy, even down to the genus level provided suitable reference species and trees are employed^24^,^25^, we proceeded with comparisons of community diversity and investigation of the taxa present. Note that all taxonomic identifications are considered putative – we report similarity of OTUs to closest matching characterized species in order to illustrate potential community members and function, rather than implying those species are actually present. Relative abundances of OTUs should be interpreted with caution due to the potential for organisms (particularly *Eucarya*) to possess multiple copies of SSU genes – see Methods for how this was addressed. The number of sequences per sample ranged from 5118 (Seal Pool 3.0 μm) to 36132 (Seal Pool 0.1 μm) (Table 2). A total of 2794 operational taxonomic units (OTUs) were formed when sequences were clustered at the level of 97% sequence identity. Seal Pool 0.1 μm filter had the greatest number of OTUs (859), followed by Atlas Pool 0.8 μm (650), while the Brown Lagoon 0.1 μm and 3.0 μm filters had the fewest (87 and 99 OTUs, respectively).

In order to correct for variation in sequencing depth, samples were rarefied to 5118 sequences before alpha-diversity metrics were calculated. Faith’s Phylogenetic Diversity (Faith’s PD) provides a measure of how widely dispersed taxa are across a phylogenetic tree^26^, and the Chao1 metric provides an estimate of richness which prioritizes the number of rare species^27^. By both of these measures, the Seal Pool 0.1 μm filter had the greatest diversity (Table 2). Samples with the lowest number of OTUs also had the fewest observed species and the lowest Chao1 estimates and Faith’s PD values (Brown 0.1 μm, Brown 3.0 μm, Atlas Cove 0.1 μm and Winston Lagoon 0.8 μm).

Shannon index is a measure of the richness and evenness of a system, and was highest for the Atlas Cove 3.0 μm sample (6.49), followed by Altas Pool 0.8 μm (5.75), Seal Pool 3.0 μm (5.61) and Seal Pool 0.8 μm (5.46) samples. The non-linearity of the Shannon index curves can make direct comparison of samples difficult, so the effective number of species was also calculated (number of equally-common species required to give a particular Shannon index value^28^). This showed the Atlas Cove sample with 660 effective species to be more than twice as diverse as the next most diverse sample (Atlas Pool 0.8 μm, 314 effective species) and 55 times more diverse than the filter with the lowest Shannon index (Brown Lagoon 0.1 μm, 12 effective species). Full alpha-diversity metrics (diversity of each site) are provided in Fig. S1.

### Overall microbial composition

**Figure 2:**
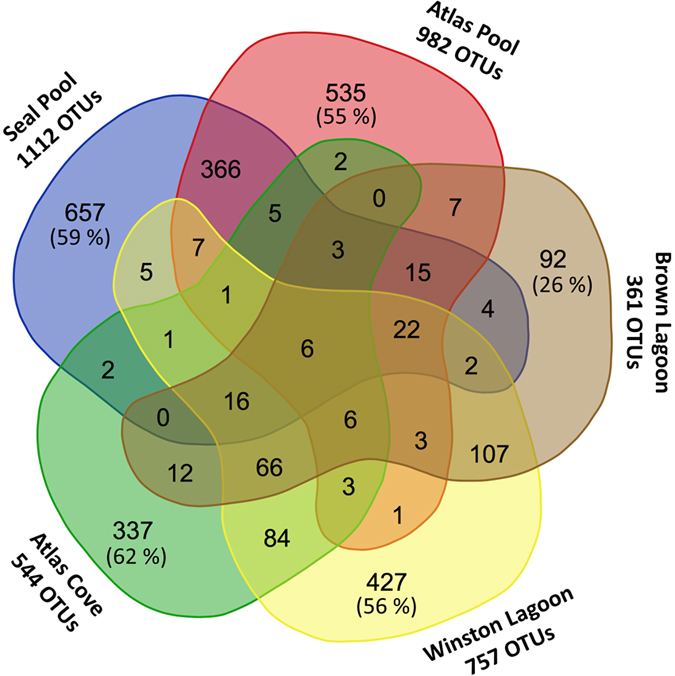
Venn diagram showing OTUs shared between the Heard Island sampling locations. Total number of OTUs per site, percentage of non-shared OTUs at each location (outer lobe), and shared OTUs are shown. The six OTUs present in all locations were denovo1904, denovo179, denovo1428, denovo2631, denovo1094 and denovo1322. Image created using the Venn diagram tool at http://bioinformatics.psb.ugent.be/cgi-bin/liste/Venn/calculate_venn.htpl.

Based on assignments of OTUs (see Methods), the distribution of taxa in each location and sample was examined. A total of six OTUs were present in all five sampling locations, with one eucaryotic and five bacterial representatives (Fig. 2 and Table S1). Their relative abundance varied between locations, with for example, the *Comamonadaceae*-related OTU denovo1428 comprising a maximum of 1.9%, 0.75%, 16.5%, 8.2% and 0.008% reads from Seal Pool, Atlas Pool, Brown Lagoon, Winston Lagoon, and Atlas Cove, respectively (Table S1). Brown Lagoon had 26% of OTUs unique to its location while the other four had ≥55% unique sequences (Fig. 2). In pair-wise comparisons, Seal and Atlas pools shared the greatest number of OTUs (366), followed by Brown and Winston lagoons (107), and Winston Lagoon and Atlas Cove (84).

A total of 47% of the Heard Island OTUs had matches ≥95% to sequences in the nt database, including 90 with 100% match. OTUs representing novel species, genera or families, included those with no matches (78 sequences), <80% identity (139), 80–90% identity (526) or 90–95% identity (710) to available sequences in the nt database. Most of the novel taxa were not very abundant, with 78 of the top 100 most abundant OTUs (totaling 82% of sequences) having a best BLAST match ≥97%. The proportion of sequences in samples that could not be taxonomically assigned was 0.4–3.1%. We acknowledge that the differential susceptibilities of particular species to lysis by the DNA extraction method used, along with possible primer bias, may mean that additional species are present on Heard Island but were not detected in this study.

#### Bacteria

**Figure 3:**
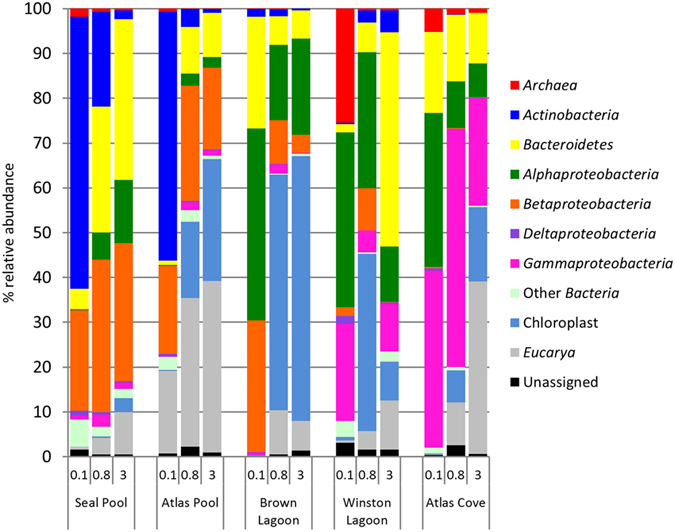
Relative abundance of the major microbial groups detected in Heard Island samples. Samples are ordered by filter size (0.1 μm, 0.8 μm and 3.0 μm) and by increasing salinity from Seal Pool to Atlas Cove.

The major bacterial phyla obtained from the Heard Island samples, namely *Actinobacteria, Bacteroidetes* and *Proteobacteria*, showed a varied distribution across the five locations and three filter sizes (Fig. 3), with the total proportion of sequences on each filter belonging to bacterial phyla ranging from 33% (Brown 3.0 μm) to 99.5% (Brown 0.1 μm). Bacteria belonging to the *Firmicutes*, Candidate Division OP11, Candidate Division OD1, *Fusobacteria*, Candidate Division TM7, *Deferribacteres, Verrucomicrobia, Cyanobacteria* and *Planctomycetes* were present at very low levels, with each representing 0.3% or less of total sequences.

**Figure 4:**
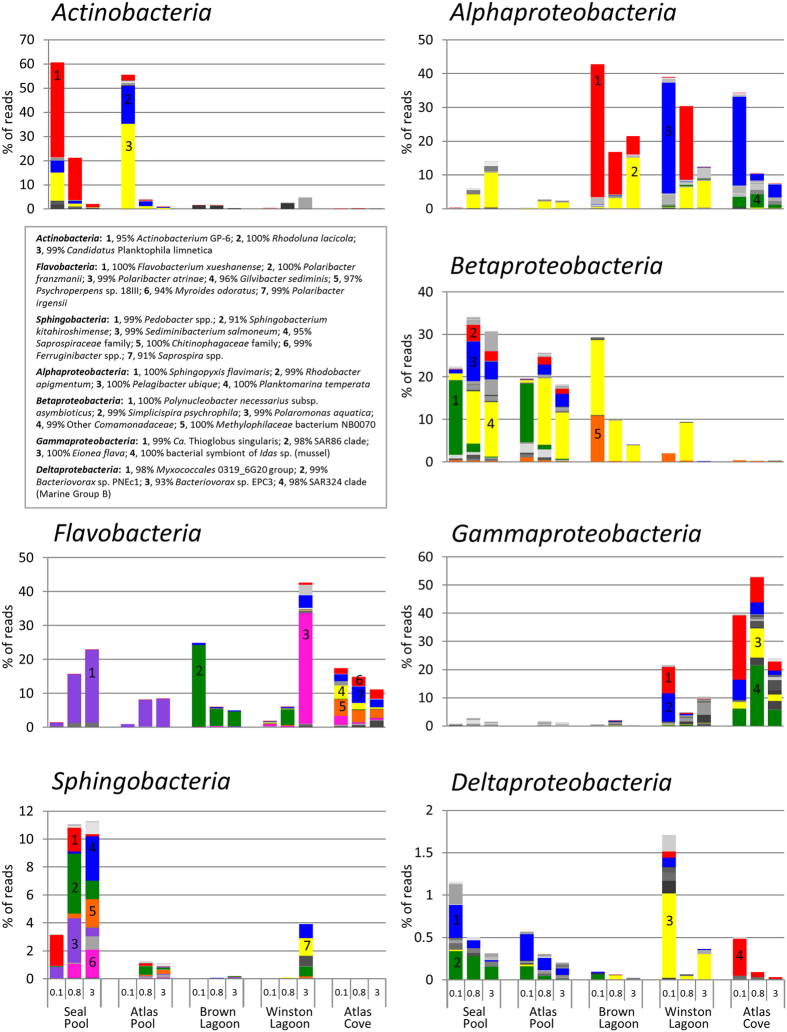
Relative abundance of OTUs for individual phyla of *Bacteria*. Colours represent different OTUs at the level of 97% sequence identity. For selected dominant OTUs an indicative best BLAST match for the taxa is shown in the key, and percent identity has been rounded to a whole number. If the taxa could not be assigned to a genus, the next most specific grouping was reported. Full OTU data is provided in Table S4. The scales for relative abundance (% of reads) vary between panels. Samples are ordered by filter size and increasing salinity as for Fig. 3.

OTUs for *Actinobacteria* comprised greater than 55% of both the Seal Pool and Atlas Pool 0.1 μm filters (Fig. 4). The predominant OTU from Seal Pool had 95% identity to *Actinobacterium* GP-6 and likely represents a novel species. In contrast, the predominant OTUs from Atlas Pool had 100% sequence identity to the well characterized *Rhodoluna lacicola* strain MWH-Ta8^29^ and 98–99% to *Planktophila limnetica* MWH-EgelM2-3.acl^30^.

*Flavobacteria* were detected in every location and filter size, but specific species dominated particular locations: OTUs with highest identity to *Flavobacterium xueshanense* from Seal Pool and Atlas Pool; *Polaribacter franzmannii* from Brown Lagoon; *Polaribacter atrinae* from Winston Lagoon. Atlas Cove was the only location to have relatively even proportions of OTUs related to multiple species of *Flavobacteria: Polaribacter irgensii, Psychropserpens* sp., *Gilvibacter sediminis* and a distant relative of *Myroides odoratus*.

Sphingobacterial OTUs from Seal Pool included those with high similarity to characterized organisms (*Pedobacter* spp., *Sediminibacterium salmoneum* and *Ferruginibacter* spp.), and low similarity to known taxa (91% to *Sphingobacterium kitahiroshimense*, and *Saprospria* spp., or best matches to *Saprospiraceae* family or *Chitinophagaceae* family); these OTUs were absent from 9 of the other 12 filters from the other four locations.

In terms of *Proteobacteria*, OTUs with 100% identity to *Pelagibacter ubique* and *Sphingopyxis flavimaris* were the dominant *Alphaproteobacteria*. Size fractionation of these OTUs was apparent in Winston Lagoon, but *Sphingopyxis* OTUs were present in all filter fractions of Brown Lagoon, and *Pelagibacter* dominated the Atlas Cove samples. Two OTUs closely related to the non-photosynthetic alphaproteobacterium *Rhodobacter apigmentum* were found in all locations except Atlas Cove. *Betaproteobacteria* included an OTU with 100% identity to *Polynucleobacter necessarius* subsp. *asymbioticus* in the 0.1 μm filters of Seal Pool and Altas Pool, and species which were classified as “Other *Comamonadaeae*” were detected in all locations except Atlas Cove. OTUs for *Gammaproteobacteria* were present at very low levels (<3% of total) in Seal Pool, Atlas Pool and Brown Lagoon, whereas the Winston Lagoon 0.1 μm filter contained members of the SAR86 clade and close relatives of the facultatively aerobic sulfur-oxidizing carbon-fixing mixotroph, *Candidatus* Thioglobus singularis PS1 (denovo611, 99.4% identity)^31^. In addition to SAR86 and *Ca*. T. singularis, the Atlas Cove samples contained high levels of sequences related to a mussel symbiont^32^ (denovo1277, 99.7% identity, 21% of reads on 0.8 μm filter) and to *Eionea flava* IMCC1962^33^ (10.4% of 0.8 μm filter). *Deltaproteobacteria* were present in low numbers with OTUs for the SAR324 clade members present from all three Atlas Cove filter fractions, an OTU with 93% similarity to a *Bacteriovorax* species dominating the Winston Lagoon cohort, and a mixture of *Myxococcales*-related OTUs and *Bacteriovorax* sp. PNec1-related (99.3%) OTUs present in both Seal and Altas pools.

#### Eucarya

The relative abundance of eucaryal sequences, detected as chloroplast 16S rRNA gene sequences and 18S rRNA gene sequences, ranged from 0.23–65.6%. For each location, the 0.1 μm filter had the fewest signatures of eucaryotes and the 3.0 μm filter had the greatest proportion, except for Winston Lagoon where the 0.8 μm filter had a higher level (44%) than the 3.0 μm filter (20%) (Fig. 3).

**Figure 5:**
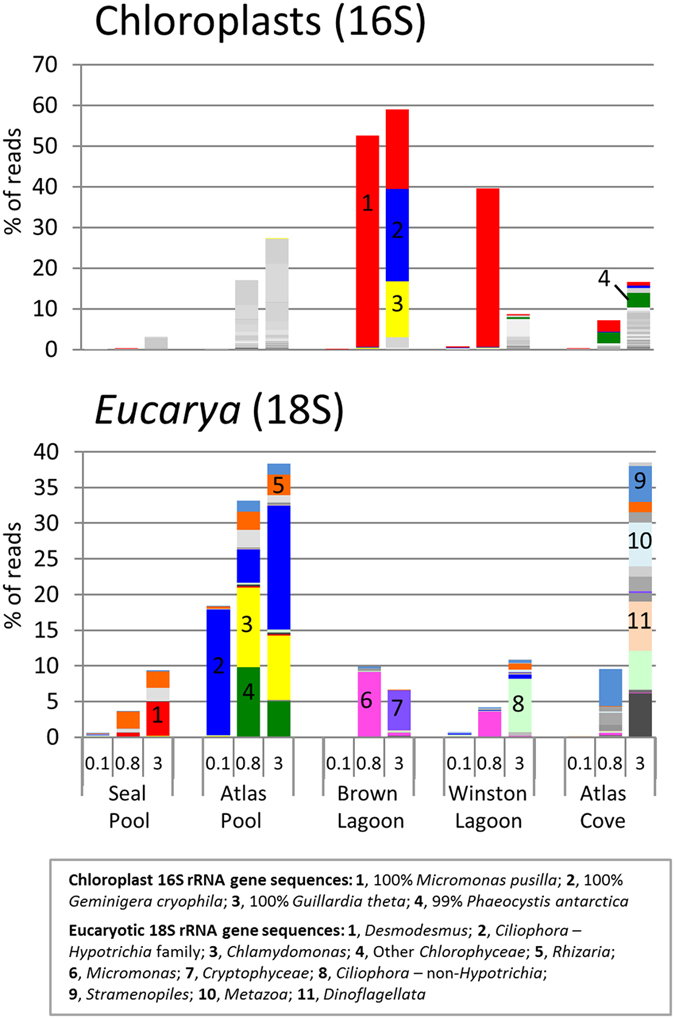
Relative abundance of OTUs for *Eucarya*. For chloroplast sequences, selected dominant OTUs and their best BLAST match are shown in the key. For *Eucarya* detected as 18S rRNA gene sequences, OTUs are grouped at Order/Family level. Note comments about the colours, sequence identity, abundance scale, sample order and OTU table as for Fig. 4.

Over 52% of the reads from the Brown Lagoon 0.8 μm filter belonged to a single OTU with 100% sequence identity to *Micromonas pusilla* chloroplast (10485 seq) (Fig. 5). This OTU was also abundant on the Winston Lagoon 0.8 μm filter, comprising 39% of total sequences. The presence of *Micromonas* in the two glacial lagoons was corroborated by the detection of 18S rRNA gene sequences for this organism from the 0.8 μm filters. In addition to the dominant OTUs matching *Micromonas pusilla* in Brown Lagoon, other OTUs were predominantly affiliated with the *Cryptophyceae* - *Guillardia theta* and the Antarctic/Sub-Antarctic species *Geminigera cryophila*^34^.

The characteristics of the other 18S rRNA gene sequences indicated the presence of location-specific eucaryotic consortia (Fig. 5). Atlas Pool had OTUs for members of the *Hypotrichia* family, *Chlamydomonas* and other *Chlorophyceae*, while Atlas Cove included *Dinoflagellata*, non-*Hypotrichia Ciliophora*, and *Metazoa*. OTUs for *Stramenopiles* and *Rhizaria* were detected in all five locations at low to moderate levels, while the main OTU detected in Seal Pool had 100% identity to *Desmodesmus communis*, a common freshwater green alga.

#### Archaea

**Figure 6:**
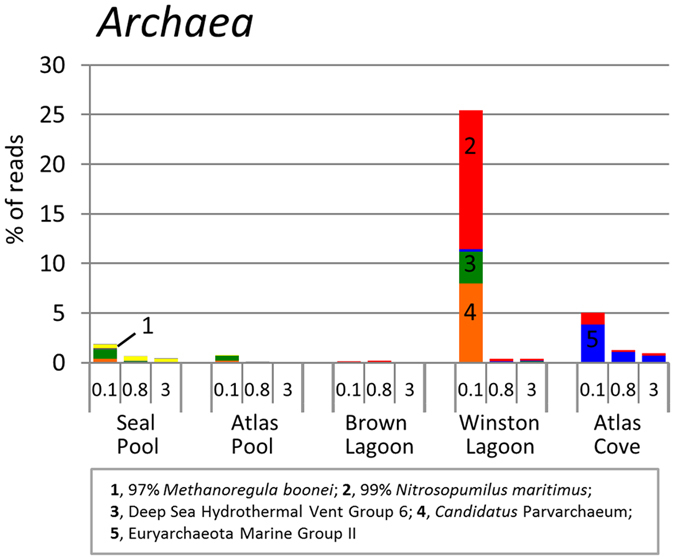
Relative abundance of OTUs for *Archaea*. Note comments about the colours, sequence identity, BLAST matches, abundance scale, sample order and OTU table as for Fig. 4.

Archaea were present at only low levels (0 to 5%) in all samples except for the Winston Lagoon 0.1 μm filter where they comprised 25.4%. OTUs belonged to four main groups (Fig. 6), with 153 OTUs from the Euryarchaeal Deep Sea Hydrothermal Vent Group 6 family (DHVEG-6) which includes *Candidatus* Parvarchaeum. The best BLAST matches related to this group of OTUs were 91.6% to the genome of Archaeon GW2011_AR15^35^, and 99.7% to 90.2% to environmental sequence data. These OTUs represent a diverse population of archaea present in Winston Lagoon (0.1 μm filter, 109 OTUs; 0.8 μm filter, 4 OTUs; 3.0 μm filter, 6 OTUs), Seal Pool (0.1 μm filter, 32 OTUs) and Atlas Pool (0.1 μm filter, 18 OTUs). The other major archaeal group of OTUs in Winston Lagoon was closely related to ammonia-oxidizing *Nitrosopumilis maritimus*. Low levels of *N. maritimus*-related OTUs were also detected in Atlas Cove and Brown Lagoon, while a small number of *Methanoregula*-related sequences were recovered from Seal Pool and Atlas Pool. Atlas Cove was the only sample in which Marine Group II (MGII) *Euryarchaeota* were detected (all three filters). Although initial classification based on the Silva database could not identify them beyond family-level, BLAST searches and construction of a 16S rRNA gene phylogenetic tree (Fig. S2) confirmed that two OTUs (denovo2505 and denovo420) were closely related to *Candidatus* Thalassoarchaea mediterranei from Marine group IIb^36^ and five OTUs (denovo486, denovo2226, denovo1995, denovo1041 and denovo1042) were closely related to the MGIIa uncultured strain MG2-GG3 for which a whole genome sequence has been reconstructed^37^.

### Community comparisons and correlation with environmental parameters

**Figure 7:**
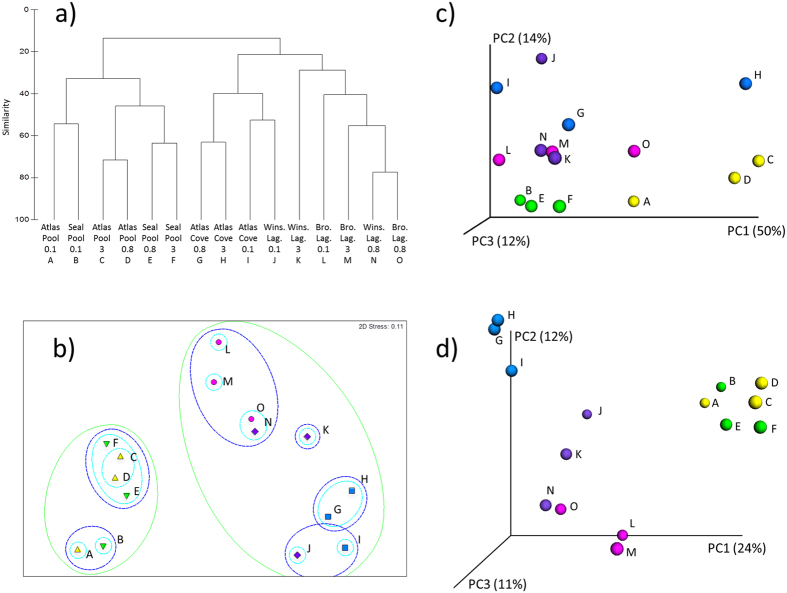
Clustering of Heard Island samples based on genus-level taxon abundance. Taxon abundance data calculated as Bray-Curtis dissimilarity, normalised and square-root transformed. Samples which clustered together with 20%, 40% and 60% similarity in the dendrogram (**a**) are circled by green, blue and aqua ovals, respectively, in the nMDS plot (**b**). Clustering based on beta-diversity measured as weighted (**c**) and unweighted Unifrac principal coordinates analysis (**d**). For beta-diversity calculations, samples were rarefied to 5118 sequences. Samples are labelled A – O as per the dendrogram (**a**), with colour coding to indicate sampling location: Seal Pool (green), Atlas Pool (yellow), Brown Lagoon (pink), Winston Lagoon (purple) and Atlas Cove (blue).

Similarities and differences between the samples were explored with Hierarchical Cluster Analysis (HCA) and non-metric multidimensional scaling (nMDS) at genus-level (Fig. 7) or kingdom/phylum-level (Fig. S3). In the clustering dendrogram, the pools clustered together on the left, while the glacial lagoons and the Atlas Cove sample grouped together on the right (Fig. 7a). Similarity Profile (SIMPROF) analysis yielded a significant Pi statistic (Pi = 9.151, p < 0.1%) rejecting the null hypothesis that no multivariate structure exists within the data, and 2-way crossed Analysis of Similarity (ANOSIM) also confirmed these differences (by location: R = 0.786, p < 0.1%; by filter size: R = 1, p < 0.3%). Pairwise testing (ANOSIM, Table S2) confirmed that the profile of Atlas Pool and Seal Pool overlapped and could not be distinguished (R = 0.111), as was the case for Winston Lagoon and Brown Lagoon (R = 0.222). The remainder of the pairwise comparisons gave values of R greater than 0.5 (all except one were close to 1) indicating the pairs of samples were well separated. These differences between locations were also reflected in the SIMPER average dissimilarity values which ranged from 60 to 92%. A full listing of which taxa most contributed to the dissimilarities between locations is provided in Table S3.

Placement of samples in 2-dimensional space by nMDS showed that the Altas Pool and Seal Pool 0.1 μm fractions grouped together (40% similarity), as did the Altas Pool 0.8 μm and 3.0 μm filters (60% similarity) and the Seal Pool 0.8 μm and 3.0 μm fractions (60% similarity) (Fig. 7b). The placement of the Brown Lagoon, Winston Lagoon and Atlas Cove samples followed a rough trajectory from least to most saline, with the 0.8 μm filters of Brown and Winston lagoons grouped together (60% similarity) and the Atlas Cove 0.8 and 3.0 μm filters grouped together (60% similarity).

The quantitative measure of beta-diversity (weighted unifrac) first dimension (PC1) explains a full 50% of the variation among samples (Fig. 7c). This measure highlights community differences among sites including changes in relative taxon abundance. The qualitative measure (unweighted unifrac), which utilizes presence/absence data but not abundance information, shows the samples from each location grouping with themselves, with those from both Atlas Pool and Seal Pool particularly near to each other (Fig. 7d). The first three dimensions of PCoA in the unweighted plot do not explain as great a total proportion of the observed variation among samples as the first dimension of the weighted plot.

In order to assess to what degree the abiotic factors (Table 1) explained the biotic patterns observed, the BEST analysis routine in PRIMER-E was employed. The null hypothesis of no correlation between biotic and abiotic factors was strongly rejected (R = 0.824, p < 0.001), with the most important single factor being either conductivity (proxy for salinity) or dissolved sulfur (equal best correlation of 0.740). The best total correlation of 0.824 was obtained when the abiotic variables conductivity, nitrite, total nitrogen and dissolved sulfur were considered.

## Discussion

This study presents the first assessment of microbial diversity in Heard Island’s aquatic systems. The distribution patterns of abundance of individual OTUs support the existence of distinct microbial communities at each location that are influenced by the environmental factors characteristic of each site (Figs 4–6). Only six OTUs were present across all five systems (Table S1), and many OTUs were only present in a single location indicating each community was likely to perform specific microbial processes. A large number of OTUs possessed little similarity to characterized strains (53%, <95% 16S/18S rRNA gene identity), underscoring the undersampling and/or novelty of microorganisms in Antarctic and Sub-Antarctic environments.

There are limits to the extent to which inferences about functional processes can be made from purely taxonomic data, with the literature supporting active discussion about methodological approaches and the validity of rationalizing function^38^,^39^,^40^,^41^,^42^. While pyrotag data are not a substitute for shotgun metagenome data^20^, this method has been successfully applied to study changes in community composition and infer microbial processes in Antarctic lakes^43^,^44^, and to assess the biogeography and effects of advection on Southern Ocean microbial communities^17^,^45^,^46^. Here we demonstrate that in combination with analyses of measured environmental variables, pyrotag sequencing can provide a relatively inexpensive approach for gaining valuable insight into distinctive ecological properties of previously unstudied sites. Naturally, functional inferences are putative and provide a broad approximation of ecosystem processes based upon the OTUs detected.

### Seal Pool and Atlas Pool

The high levels of nutrients evident in Seal Pool (Table 1) are provided by seals in and around the pool, and the wet mixed herbfield vegetation nearby (Fig. 1). Eucaryotic OTUs, including those related to the photosynthetic green alga *Desmodesmus*, and OTUs for *Cyanobacteria* were detected at low relative abundance, possibly indicating that limited primary production was occurring (Figs 5 and 6). OTUs related to bacteria that are capable of organic matter turnover, including *Actinobacteria, Sphingobacteria* and *Flavobacteria*, were abundant (Fig. 5). The *Actinobacteria* have been linked to Antarctic lake eutrophication and *Flavobacteria* to degradation of Antarctic aquatic detritus^19^,^47^,^48^, and *Sphingobacteria* more broadly to a degradative capacity^49^. *Myxococcales* were also present and likely contribute to degradation of organic matter^49^.

The bacterial OTUs for Seal Pool are also characterized by a relatively high degree of novelty compared to characterized strains. For example, the most abundant *Actinobacteria* OTU has 95% identity to *Actinobacterium* GP-6, and *Sphingobacteria* have <91% identity. It is possible that the novel taxa in Seal Pool may possess novel physiology or particular adaptations allowing them to dominate in this environment.

The presence of OTUs related to *Bacteriovorax* suggests bacterial predation as these organisms feed on Gram-negative prey^50^. This activity would also contribute to the release and recycling of organic matter. Denitrifying *Betaproteobacteria* also appear to be present, as OTUs with 99–100% identity to *Comamonas* spp. and *Simplicispira psychrophila* were identified; bacteria that are known to perform chemoorganotrophic growth^51^,^52^,^53^. OTUs matching the ubiquitous and cosmopolitan heterotrophic freshwater bacterium, *P. necessarius* subsp. *asymbioticus*^54^ were also abundant in Seal Pool. All of these findings are consistent with the rich organic matter of Seal Pool supporting a microbial ecosystem driven by heterotrophic degradation.

The moderate relative abundance of OTUs (Table S4) for chloroplasts and multiple types of green algal 18S rRNA genes (*Chlamydomonas* and Other *Chlorophyceae*, Fig. 5.) suggest primary production plays a much greater role in Atlas Pool than Seal Pool. The eucaryal OTUs included *Halteria* sp. bLaN2 (98.5% identity) and the abundance of such ciliates has been shown to correspond to summertime runoff of nutrients, with this marine species involved in the incorporation of particulate and dissolved organic carbon into the food web^55^. *Alphaproteobacteria* and *Gammaproteobacteria* are almost entirely absent from Atlas Pool. In contrast, the communities of *Flavobacteria, Betaproteobacteria* and *Deltaproteobacteria* largely mirror that of Seal Pool suggesting they play a similar role in turnover of organic matter. OTUs for *Sphingobacteria* are greatly reduced in Atlas Pool compared to Seal Pool. This may indicate that the nutrients metabolized by *Sphingobacteria* in Seal Pool have been degraded (oxidized) by the time any runoff reaches Atlas Pool (see pool locations Fig. 1; DOC of 170 μg C L^−1^ in Seal pool c.f. 12 μg C L^−1^ in Atlas Pool, Table 1).

The abundance of *Actinobacteria* appears similar to that of Seal Pool, but the proportions of specific types are swapped with OTUs closely related to *Rhodoluna lacicola* and *Candidatus* Planktophila limnetica dominant over those for *Actinobacterium* GP-6. *Cand*. P. limnetica has only been isolated in stable co-culture with *P. necessarius* subsp. *asymbioticus*, with the latter likely providing some essential nutrients for growth^30^. *R. lacicola* and *Cand*. P. limnetica both possess actinorhodopsins for photoheterotrophy^30^,^56^,^57^ and have been reported as dominant members of some freshwater lakes^29^,^58^,^59^. The detection of OTUs matching these bacteria in Atlas and Seal pools suggests that interspecies interactions may have important roles in these less saline food webs.

### Brown Lagoon

Brown Lagoon had the lowest levels of nitrogen (Table 1) and the lowest community diversity (Table 2) and was dominated by OTUs matching the unicellular photosynthetic picoflagellate *Micromonas pusilla* (both 18S rRNA and chloroplast 16S rRNA gene sequences) (Fig. 5). This picoflagellate can be numerically dominant in tropical, temperate and polar waters, is very small in cell size (<2 μm) with a small genome (15 Mb), and is able to effectively scavenge ammonium under nitrogen limiting conditions^60^,^61^. The draft genome of an Antarctic *Micromonas* sp. is reported to encode many enoyl-CoA hydratases which may enhance fatty acid metabolism, and an antifreeze protein^62^. Such properties would likely contribute to the ability of Antarctic *Micromonas* spp., including those in Brown Lagoon, to grow in the cold.

OTUs for *Sphingopyxis* (99.6% similarity to *S. flavimaris*) were abundant, particularly on the 0.1 μm filter (39%) compared to 0.8 μm (12%) and 3.0 μm (5%) filters. The increase in OTU abundance with decreasing filter size may indicate the Brown Lagoon species have properties in common with *Sphingopyxis alaskensis*, which was isolated from cold Alaskan waters, the North Sea and the North Pacific, has a small cell size and can thrive under oligotrophic conditions^63^,^64^,^65^. The abundance (16.5%, 0.1 μm filter) of an OTU 99% similar to the recently isolated oligotrophic *Comamonadaceae* bacterium LSUCC0123^66^ is consistent with Brown Lagoon supporting the growth of oligotrophic bacteria. Oligotrophic bacteria may be innately suited to Antarctic environments due to their ability to weather periods of energy limitation^67^.

OTUs were also abundant for close relatives of *Methylophilaceae* bacterium strain NB0070 (10.7%, 0.1 μm filter) indicating a capacity to metabolize methanol and high molecular weight organic matter^68^, and the psychrophilic heterotrophic *Polaribacter franzmanii* (24%, 0.1 μm filter) indicating an ability to degrade algal polysaccharides and proteins^69^,^70^. Overall, the OTU data suggest Brown Lagoon is characterized by *Eucarya* and *Bacteria* with relatively small cell sizes, oligotrophic strategies, and capacities to metabolize organic matter (including of high molecular weight).

### Atlas Cove and Winston Lagoon

The major taxa of the marine Atlas Cove community consisted of OTUs for *Gammaproteobacteria (Eionea, Thioglobus*), *Alphaproteobacteria* (SAR11, *Roseobacter*), and *Flavobacteria*, with primary producers being *Eucarya (e.g. Phaeocystis antarctica*) rather than cyanobacteria; findings typical of Southern Ocean surface waters^48^,^71^. A notable difference was for *Archaea*, with a higher proportion of OTUs for MGII *Euryarchaeota* than for Marine Group I nitrifying chemolithoautotrophs (e.g. *Nitrosopumilus maritimus*); the latter being reported to dominate Southern Ocean surface waters^19^,^71^. Genome reconstruction of MGII strains indicates they are facultative photoheterotrophs that can degrade organic carbon^36^,^37^,^72^. The OTUs from Atlas Cove had a wide-range of identity (84–99%) to known MGII strains indicating they may possess novel physiological traits.

In Winston Lagoon, the low relative abundance of OTUs for *Eucarya* (18S rRNA and chloroplast genes), but high representation (31% total reads) of *Flavobacteria* (99.7% identity to *Polaribacter atrinae*), is likely to indicate sampling occurred towards the end of an algal bloom cycle. Consistent with this, an unusually high level of brown material (*e.g*. algal detritus) was present on the 20 μm prefilter that was used to filter samples. *P. atrinae* possesses a wide array of amidase, DNase, esterase, lipase, phosphatase and sugar degradative enzymes^73^, and *Polaribacter* spp. are known to degrade algal polysaccharides and proteins and can represent a high proportion of the population during Antarctic algal blooms^19^,^48^,^70^,^74^, including in the iron-rich waters on the Kerguelen Plateau^75^. In coastal Southern Ocean waters, *Alphaproteobacteria* utilize labile substrates liberated from algal detritus by *Flavobacteria*^48^. The abundance (~30–40%) of OTUs for *Alphaproteobacteria* (e.g. *Pelagibacter ubique* and *Sphingopyxis*) on 0.1 and 0.8 μm filters, and *Flavobacteria* (~40%) on 3.0 μm filters, suggests these bacterial taxa in Winston Lagoon associate with detritus from algal blooms in a similar way to their oceanic counterparts. *Sphingobacteria* (26 OTUs on the 3 μm filter) were also likely to have been involved in algal organic matter turnover^49^, whereas OTUs with 99% identity to *Nitrosopumilus maritimus* indicate possible chemolithoautotrophic or mixotrophic ammonia-oxidiation being performed by *Archaea*.

Being open to the ocean, the salinity and nutrient composition of Winston Lagoon was much more similar to Atlas Cove than Brown Lagoon (Table 1), and the presence of *Gammaproteobacteria* in Winston Lagoon was an indicator of oceanic influx as this class of *Bacteria* was abundant in Atlas Cove but essentially absent in Brown Lagoon, Atlas Pool and Seal Pool (Fig. 4). Interestingly, Winston Lagoon lacked the signatures of the types of *Eucarya* present in Atlas Cove, suggesting the environmental conditions were sufficiently distinct that oceanic *Stramenopiles, Metazoa* and dinoflagellates currently struggle to colonize Winston Lagoon (Table S4). As a proxy for Antarctic freshwater glacial lagoons experiencing marine influx, Winston Lagoon will be valuable to monitor to assess whether salinity levels and community composition remain stable. By determining if total diversity increases or if endemic species decline, we will be able to learn about the interplay between species competing for changing niches.

### Factors influencing each ecosystem

Location specific factors can greatly influence microbial communities^76^,^77^,^78^. In wetland streams, dissolved organic matter (DOM) was identified as the main factor controlling community composition^77^, whereas microbial populations ultimately persisted in a tundra lake despite seasonal inputs of DOM and allochthonous species^76^. In a lake on Ardley Island (north of the Antarctic Peninsula), the bacterial sediment community correlated with the historical presence of colonizing penguins, and elemental composition of penguin guano influenced the relative abundance of many taxa^78^. Here we show for the Heard Island systems that salinity, dissolved sulfur, total nitrogen and nitrite are important for explaining the present patterns of community composition. Detailed investigation of S- and N- cycling in the Heard Island pools and lagoons will be of interest for further study – for example, examining the role of dissolved sulfur in Winston Lagoon in supporting sulfur oxidizers such as *Cand*. Thioglobus singularis^31^, which in turn provide organic reduced sulfur for species like SAR86 that lack the capacity to reductively assimilate sulfate^79^.

In addition to water chemistry, we can comment on the possible influences of dispersal, limnology and biological factors. Strong winds across the island would undoubtedly cause Aeolian transport of microorganisms. However, our data shows that communities are largely specific to each location indicating that environmental selection provided by each system overcomes the effects of dispersal. The ground around Brown and Winston lagoons includes recently deglaciated rock and moraine which does not yet support complex vegetation, and glacial meltwater travels a greater distance across deglaciated ground to reach Brown Lagoon compared to Winston Lagoon where the glacier still calves into the lagoon. In addition to water source differences between the five locations (rain, snow, glacier, marine), the microbial communities in the pools on the Azorella Peninsula will be influenced by the high nutrient and microbial inputs from elephant and fur seal excreta and runoff from herbfield vegetation and soil. The lagoons are much larger bodies of water so penguin and seal input will have less influence, although the different seal populations breeding near each lagoon (Brown Lagoon, fur; Winston Lagoon, elephant) may seed each with animal specific microorganisms (Fig. 1). The effects of climate change, particularly glacial retreat and formation of new lagoons, are expected to cause the redistribution of plants, sea birds and seal populations^80^, thereby influencing nutrient and microbial inputs into lagoons and pools across the island. Cooperation between microbial community members may also have an important influence, as this has been observed for some Antarctic sea ice, lake and oceanic communities^20^,^81^, and our data suggests as-yet unstudied factors may influence the syntrophic interactions between *Cand*. P. limnetica and *P. nucleobacter* subsp. *asymbioticus*^30^, allowing *Cand*. P limnetica to become the dominant *Actinobacteria* in Atlas Pool but not Seal Pool. In future work, it will be important to augment understanding of the food web relationships by also including the potentially important roles of viruses^20^.

### Perspective

The range of environmental parameters, community structures, and putative functions observed across the five study sites suggests a significant reservoir of microbial biodiversity is found on the island. Are these snapshots of a stable community structure; communities which periodically oscillate between several stable states; a temporary response to perturbation; or, environments in genuine and ongoing flux due to climate change? To answer these questions, temporal studies covering days, seasons and years need to be performed^20^,^82^. Obtaining sufficient replicates will be necessary for distinguishing random fluctuations from genuine trends. It will be no small challenge to achieve all this while recognizing Heard Island’s World-Heritage status and preserving and protecting its unique and valuable natural resources. However, it is important to perform this research in view of the rapid changes occurring on the island, as the findings will heighten awareness amongst policy makers of how urgently we need international action to mitigate the effects of anthropocentric climate change.

## Methods

### Study sites and sampling

Five study sites were selected around Heard Island (Fig. 1 and Table 2). Seal Pool (name designated in this study) is a small shallow pool formed by elephant seals within wet mixed herbfield vegetation (Tussock grass, Cushion plants and Kerguelen Cabbage). Its waters were visibly eutrophic (green) at the time of sampling. Nearby Atlas Pool (name designated in this study) is a moderately sized shallow pool located to the west and shorewards of Seal Pool. Both of these pools lie on the Azorella Peninsula and receive freshwater from snow melting rather than from glacial sources, as well as rain, with Seal Pool likely draining towards Atlas Pool. Large populations of elephant and fur seals, macaroni, rockhopper, gentoo and king penguins are prevalent on the peninsula and nearby Nullarbor Plain.

Brown Lagoon was first observed in 1963 when Brown Glacier retreated 100 m inland leaving a small lagoon separated from the ocean by a boulder beach^14^. By 1971 the glacier had retreated a further 350 m creating a proglacial lagoon, and the current size is ~900 m long along the coastal edge and ~600 m wide at the widest point (calculated from maps^3^,^83^). A gravel bar separates the lagoon from the ocean, and the wildlife present are fur seals and macaroni, rockhopper, gentoo and king penguins. Winston Lagoon formed prior to the commencement of glacial monitoring in 1947, is one of the largest glacial lagoons on the island, and is currently ~2.3 km long along the coastal edge with a maximum width of ~2.3 km (calculated from maps^83^). Winston Glacier still terminates at and calves ice into the lagoon, and the wildlife in this region are mostly elephant seals and gentoo or king penguins, with the closest fur seals recorded at Paddick Valley to the east. Coastal prograding has occurred and although the opening from Winston Lagoon to the sea first observed in 1947 appeared to be superficially blocked in the 1980s^2^, it was open to the ocean at the time of sampling.

The marine sample from the site designated Atlas Cove was collected while the research vessel *Aurora Australis* was moored near Atlas Cove between Lauren’s Peninsula and the Azorella Peninsula (Fig. 1), and shotgun metagenome data from this site was previously described (Sample ID GS394^84^). Here, DNA was used for pyrotag sequencing with the SSU rRNA gene data being representative of the marine environment adjacent to Heard Island and serving as a comparison to the pool and lagoon study sites.

Water samples were collected on the 16^th^ December (Atlas Lagoon, 2 L; Seal Pool, 1.75 L) or 17^th^ December 2008 (Brown Lagoon, 17.4 L; Winston Lagoon, 13.4 L; Atlas Cove, 400 L). Biomass was harvested concurrently with- or within a few hours of water collection by passing the water from each location through a 20 μm pre-filter, and serially size fractionating through 293 mm diameter polyethersulfone membrane filters (Pall, Port Washington, USA) with 3.0, 0.8 and 0.1 μm pore sizes, as described previously^17^,^18^,^85^. Water sample collection and processing at Atlas Lagoon and Seal Pool were terminated at relatively small volumes (1.75–2 L) due to biomass saturation of the filters. All filters were placed in storage buffer, frozen in liquid nitrogen and cryogenically maintained at −80 °C until processing could be performed at the University of New South Wales (Sydney, Australia), as described previously^17^,^18^.

### Water analysis

Analysis of water composition (DOC, nitrate, nitrite, ammonia, total nitrogen, total dissolved nitrogen, dissolved reactive phosphorous, total phosphorus, total dissolved phosphorus, total sulfur and total dissolved sulfur) was performed by Analytical Services, Tasmania according to American Public Health Association’s Standard Methods, as previously described^86^. Nutrients were measured from water collected after filtration through the on-site 20 μm pore size pre-filter (250 ml), with dissolved nutrients measured after filtration through a 0.1 μm pore size membrane filter (250 ml).

### DNA extraction

DNA extraction was performed on each entire filter using a phenol-chloroform method^85^ with modifications^87^. Briefly, filters were cut into fine strips (~3 mm × 10 mm) and incubated shaking in sucrose lysis buffer (final concentration 25 mM EDTA, 25 mM EGTA, 0.75 M sucrose, 5 mM Tris-HCl [pH 8.0]) with 2.5 mg mL^−1^ lysozyme for 30–60 min at 37 °C. Proteinase K was added to a final concentration of 200 μg mL^−1^ and three freeze-thaw cycles (−80 °C for 20–30 min, 55 °C for 20–30 min) were performed. An additional 200 μg mL^−1^ of Proteinase K, and SDS to a final concentration of 1% were added, and samples incubated in a shaking water bath at 55 °C for 2 h. This was followed by two extractions with phenol and one extraction with phenol/chloroform/iso-amyl alcohol (25:24:1). DNA was precipitated with sodium acetate and 1-propanol, and the pellet washed with 70% ethanol and dried before re-suspension in TRIS-EDTA buffer. All reagents were from Sigma-Aldrich (St. Louis, USA).

### 454 pyrotag sequencing and data processing

Amplification of SSU rRNA gene sequences and pyrosequencing was carried out at the Research and Testing Laboratory (Lubbock, TX, USA) using Roche 454 sequencing technologies and methodology. Samples were amplified for pyrosequencing using a forward and reverse fusion primer. The forward primer was constructed with (5′-3′) the Roche A linker (CCATCTCATCCCTGCGTGTCTCCGACTCAG), an 8 bp barcode to multiplex samples, and the 926wF primer (AAACTYAAAKGAATTGRCGG^88^). The reverse fusion primer was constructed with (5′-3′) a biotin molecule, the Roche B linker (CCTATCCCCTGTGTGCCTTGGCAGTCTCAG), and the 1392R primer (ACGGGCGGTGTGTRC^89^). These primers amplify the V6-V8 region of the SSU rRNA gene and were selected as they cover about 84%, 71% and 91% of 16S rRNA gene sequences from *Bacteria, Archaea* and *Eucarya*, respectively, based on interrogation of sequences spanning the V6-V8 region in the curated SILVA Ref NR SSU rRNA gene dataset. This coverage was determined using TestPrime^90^ (https://www.arb-silva.de/search/testprime/) with a stringency setting of 1 bp mismatch permitted but 0 bp mismatches allowed in the 5 bases at 3′ end, and phylogenetic coverage details for 926wF - 1392R are provided in Table S5 and S6.

Amplifications were performed in 25 μl reactions with Qiagen HotStar Taq master mix (Qiagen Inc, Valencia, California), 1 μl of each 5 μM primer, and 1 μl of template. Reactions were performed on ABI Veriti thermocyclers (Applied Biosytems, Carlsbad, California) under the following thermal profile: 95 °C for 5 min, then 35 cycles of 94 °C for 30 sec, 54 °C for 40 sec, 72 °C for 1 min, followed by one cycle of 72 °C for 10 min and 4 °C hold. Amplification products were visualized with eGels (Life Technologies, Grand Island, New York). Products were pooled with equimolar quantities and each pool was cleaned and size selected using Agencourt AMPure XP (BeckmanCoulter, Indianapolis, Indiana) following Roche 454 protocols (454 Life Sciences, Branford, Connecticut). Size selected pools were quantified and diluted to be used in emulsion-based clonal amplifications (emPCR), which were performed and subsequently enriched. Sequencing followed established manufacturer protocols (454 Life Sciences) using Roche reagents and the GS FLX platform (Roche, Branford, USA). Denoising (using USEARCH^91^), chimera checking (using the *de novo* method built into UCHIIME^92^), and removal of poor quality reads (criteria: failed sequence reads; sequences with low quality tags, primers or ends; sequences less than 250 bp in length) were performed by the sequencing facility. The raw data have been deposited in the Sequence Read Archive with BioProject number: PRJNA335685.

Data were processed using the Quantitative Insight Into Microbial Ecology (QIIME) pipeline v1.8.0 with default parameters^22^. Briefly, samples were de-multiplexed and filtered for quality (length range 200–1000 bp; number of ambiguous bases must not exceed 6; mean quality score must be 25 or greater; maximum homopolymer run of 6 bp; zero mismatches in primer), then *de novo* OTUs were picked and assigned taxonomy^93^ by reference with the Silva 111 dataset^94^ using UCLUST^91^ with clustering at 97% identity as this threshold has been shown to substantially reduce artificial inflation of diversity estimates due to pyrosequencing errors^95^. A full OTU table with relative abundances, Silva-based classification and best BLAST match of all OTUs is provided in Table S4. The number of 16S rRNA genes may vary between different species of *Bacteria* and *Archaea*, and *Eucarya* may possess large numbers of 18S rRNA genes and/or chloroplasts (including multiple copies of 16S rRNA genes within each chloroplast)^96^, thereby precluding accurate estimates of the true abundance of species from the relative abundances of SSU rRNA gene sequences. Nevertheless, no ‘correction’ was applied to adjust relative abundances as the likelihood of performing this accurately is low given the novelty of the systems studied and demonstration that a high proportion of OTUs had low identity to characterized bacterial, archaeal and algal species. Sequences were also aligned using PyNAST^97^ and used to construct a phylogenetic tree using FastTree^98^. Alpha-diversity (shannon, simpson, goods_coverage, PD_whole_tree, chao1, and observed_species) and Beta-diversity (weighted and unweighted unifrac^99^, principal coordinate analysis) metrics were calculated in QIIME and visualized using Emporer^100^. The relatedness of Heard Island taxa to their closest characterized relatives was assessed by BLAST against the NCBI nt database with environmental and metagenome sequences removed^101^. Relatedness to all known taxa was assessed by BLAST against the full NCBI nt database.

Abundance counts of taxa summarized at Level 2 (Kingdom – Phyla) and Level 6 (Kingdom – Phyla – Class – Order – Family – Genus) were converted into relative percentages for each of the 15 samples and a Bray-Curtis dissimilarity matrix^102^ was generated in PRIMER-E v6^103^ from square-root transformed data in preparation for statistical analysis. Water chemistry data were also transformed and normalized, and Principal Component Analysis (PCA) was performed. Taxonomic composition-based similarities between samples were investigated using Cluster (HCA), and the non-parametric permutation procedure ANOSIM was used to test the differences in composition of the communities detected in each location and at each filter size. Evidence of structure in an *a priori* unstructured set of samples was assessed using global SIMPROF analysis. nMDS was used to plot the position of each sample based on its distance from all other samples, and then used for BEST analysis. BEST analysis compares the 2-dimensional distribution of samples obtained based on biotic (taxonomic) factors and abiotic factors (water chemistry data) to identify which factors best explained the observed patterns. The contribution of particular genera to the observed dissimilarity between groups in the nMDS plots was calculated using SIMPER (similarity percentages procedure). The ANOSIM, BEST, Cluster, nMDS, PCA, SIMPER and SIMPROF statistical tests were all performed using the software PRIMER-E v6^103^ with recommended settings.

## Additional Information

**How to cite this article:** Allen, M. A. and Cavicchioli, R. Microbial communities of aquatic environments on Heard Island characterized by pyrotag sequencing and environmental data. *Sci. Rep.* **7**, 44480; doi: 10.1038/srep44480 (2017).

**Publisher's note:** Springer Nature remains neutral with regard to jurisdictional claims in published maps and institutional affiliations.

## Supplementary Material

Supplementary Information

Supplementary Table S4

Supplementary Table S5 and S6

## Figures and Tables

**Table 1:** Water composition analysis.

Analyte	Units	Seal Pool	Atlas Pool	Brown Lagoon	Winston Lagoon	Atlas Cove
Conductivity	μS cm^−1^	715	2000	6580	39800	48200
Ammonia	mg N L^−1^	24.9	7.18	0.01	0.011	0.036
Nitrate	mg N L^−1^	1.48	4.32	<0.002	0.713	0.391
Nitrite	mg N L^−1^	0.022	0.091	<0.002	<0.002	0.005
Nitrogen, total filtered^1^	mg N L^−1^	27	13	<0.04	0.89	0.63
Nitrogen, total^2^	mg N L^−1^	nd^3^	18	0.19	0.88	0.63
Phosphorus, dissolved reactive	mg P L^−1^	0.03	0.043	0.025	0.073	0.06
Phosphorus, total filtered^1^	mg P L^−1^	0.082	0.115	0.027	0.124	0.092
Phosphorus, total^2^	mg P L^−1^	nd^3^	1.7	0.052	0.125	0.124
S dissolved	μg S L^−1^	8870	20200	69000	671000	771000
S total^2^	μg S L^−1^	nd^3^	16500	74900	609000	851000
DOC	μg C L^−1^	170	12	27	14	34

Abbreviations: nd, not determined; DOC, dissolved organic carbon.

^1^0.1 μm filtrate recovered after biomass filtration.

^2^Sample water filtrate through 20 μm pre-filter.

^3^Sample volume insufficient to permit analysis.

**Table 2:** Diversity metrics for SSU rRNA gene sequences obtained by 454 pyrotag sequencing.

Sample Site	Seal Pool			Atlas Pool			Brown Lagoon			Winston Lagoon			Atlas Cove		
Location	−53.0183417°, 073.3922250°			−53.0195361°, 073.3895833°			−53.0690111°, 073.6648083°			−53.1503972°, 073.6760667°			−53.0125000°, 073.3706667°		
Sample ID	509	510	511	490	489	488	572	571	570	543	542	541	538	539	540
Filter size (μm)	0.1	0.8	3	0.1	0.8	3	0.1	0.8	3	0.1	0.8	3	0.1	0.8	3
No. of sequences	36132	5323	5118	13121	26968	18150	12472	20162	7678	8118	8647	6832	9518	11990	8480
No. of OTUs at 97% similarity	859	287	271	337	650	416	87	277	99	392	218	388	176	317	365
Observed species* (Av. ± std. dev)	391 ± 10	283 ± 1	271 ± 0	237 ± 10	367 ± 9	275 ± 5	63 ± 5	151 ± 8	90 ± 3	339 ± 4	173 ± 6	363 ± 5	148 ± 3	244 ± 5	321 ± 6
Chao1* (Confidence interval)	719 (615–872)	337 (314–377)	289 (280–309)	360 (312–438)	579 (507–689)	406 (355–489)	93 (74–145)	281 (221–390)	115 (100–154)	441 (404–498)	263 (223–335)	431 (405–474)	198 (173–248)	360 (313–441)	396 (365–447)
Faith’s Phylogenetic diversity*	35.5	25.1	22.2	25.1	27.2	20.5	8.9	15.7	9.8	32.8	17.2	28.1	12.5	20.5	21.6
Shannon H’ *	4.46	5.46	5.61	4.10	5.75	5.13	2.51	2.79	3.50	4.52	3.38	5.34	4.11	5.10	6.49
Effective species (e^H’)*	87	235	272	60	314	168	12	16	33	92	29	210	61	164	660
Simpson (1-D)*	0.829	0.943	0.944	0.867	0.954	0.935	0.748	0.693	0.862	0.855	0.787	0.890	0.868	0.926	0.979

^*^Alpha diversity calculations are based on rarefaction to 5118 sequences.
