# Supplementary material for: Microbial communities of aquatic environments on Heard Island characterized by pyrotag sequencing and environmental data
Source: Sci Rep. 2017 Mar 14;7:44480. doi: 10.1038/srep44480 (PMC5349573; doi:10.1038/srep44480)
Supplement: Supplementary Information [file srep44480-s1.pdf]

Microbial communities of aquatic environments on Heard Island characterized by pyrotag sequencing and environmental data

Michelle A. Allen and Ricardo Cavicchioli.

Supplementary Information

Supplementary Results
Figures S1 – S3
Tables S1 – S6 (note Table S4-S6 provided separately as excel files)

Supplementary Results

Clustering at the phylum level. To assess similarities and differences between the samples at phylum-level resolution, taxonomic data and sample profiles were analyzed by HCA and a heat map was constructed (Fig. S3). The Seal Pool and Atlas Pool 0.1 µm filters clustered together, as did the Atlas Cove and Brown Lagoon 0.1 µm filters, while the 0.8 µm and 3 µm filters from several locations clustered together (Seal Pool, Atlas Pool, Atlas Cove). The two-way crossed ANOSIM was unable to reject the null hypothesis of no difference between groups by location (R= 0.083, p=0.313) but the null hypothesis of no difference between groups by filter size was rejected at this level of taxonomic resolution (R= 0.8, p=0.009). Global SIMPROF (similarity profile) analysis, which tests for evidence of structure in an *a priori* unstructured set of samples, also strongly rejected the null hypothesis of no multivariate structure among the 15 samples (Pi = 3.957, p=0.001).

Figure S1. Diversity metrics for the Heard Island samples. Alpha diversity measured as a) Observed species, b) Shannon index, c) Faith's Phylogenetic Diversity, and d) Chao1 estimate.

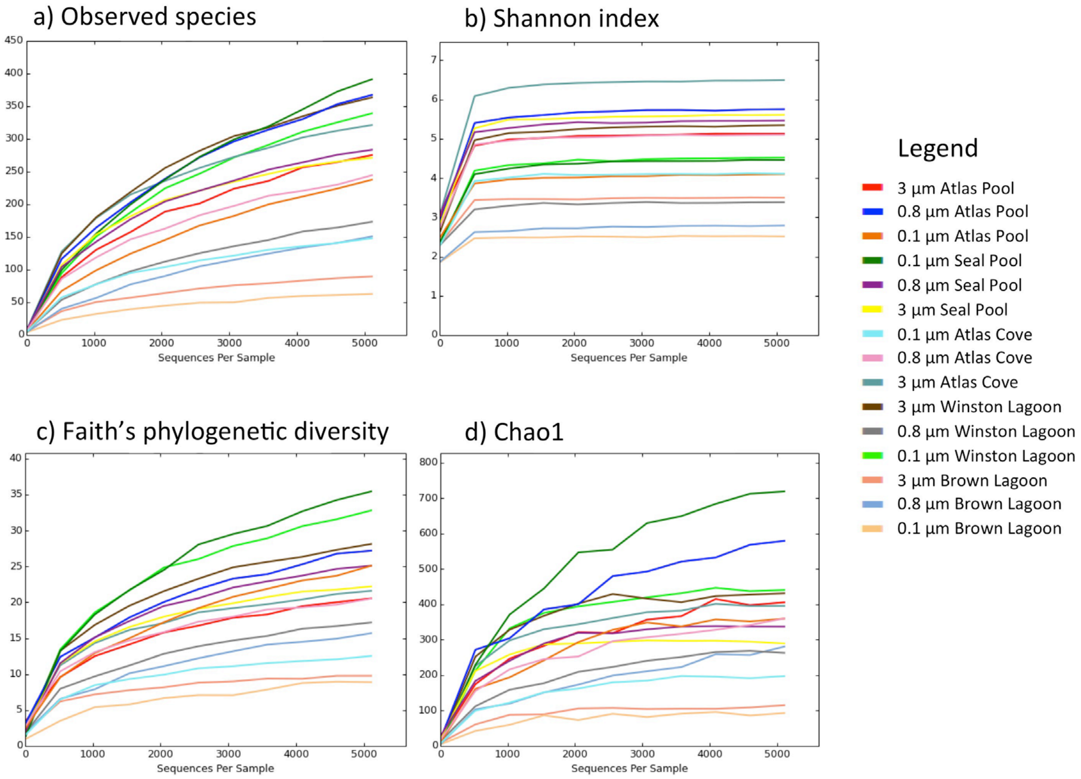

Figure S2. Phylogenetic tree of 16S rRNA genes belonging to the *Euryarchaeota* Marine Group II. Representative sequences of Heard Island archaeal OTUs (denoted 'denovo') were aligned with reference species from the *Thermoplasmatales* and Marine Group II using Clustal in MEGA6. A maximum-likelihood tree was inferred and the tree with the highest log likelihood is shown. *Halorubrum lacusprofundi* was used to root the tree and bootstrap values greater than 50 % are shown at the respective nodes.

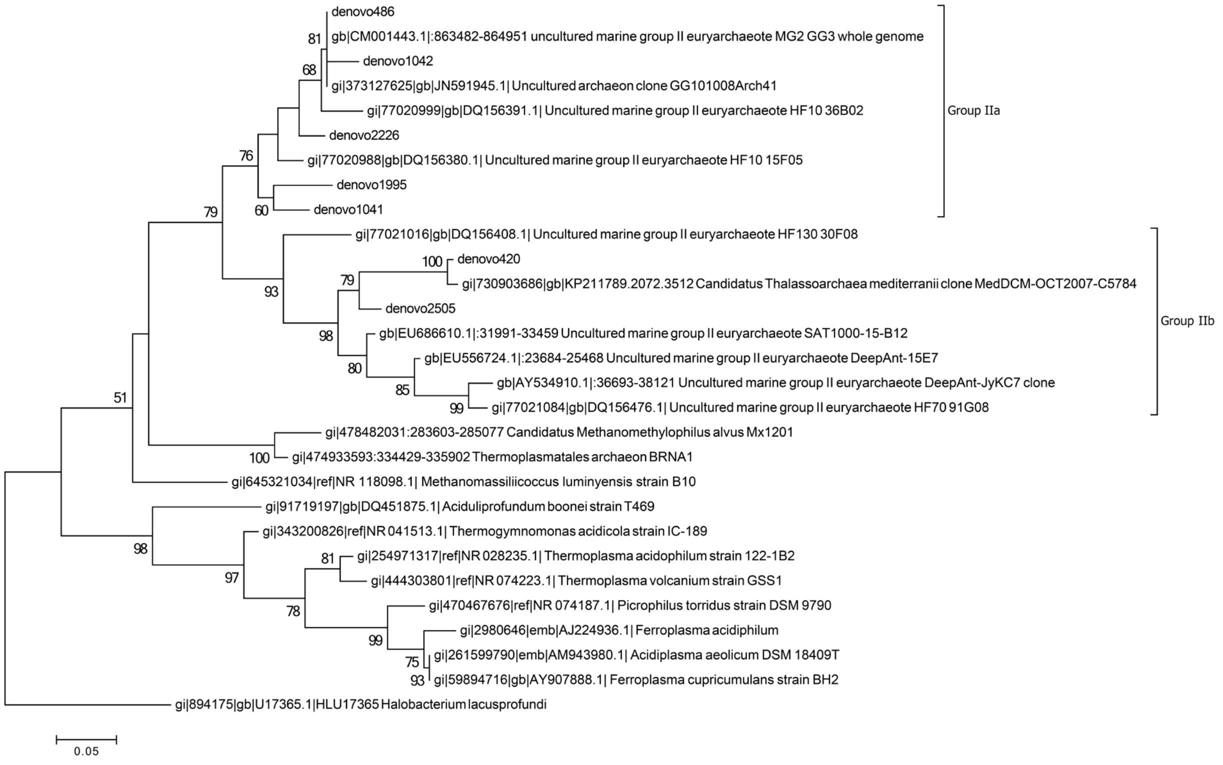

Figure S3. Comparisons of Heard Island samples when SSU rRNA gene sequences were classified to the level of phylum. Sequences that could not be assigned with > 85 % bootstrap confidence were designated "unassigned". SSU rRNA gene counts were normalized and square root transformed. a) Heatmap and biclustering plot with samples labeled according to filter size (0.1, 0.8 and 3.0 µm) and location. Heatmap colour scale is from blue (least abundant) to yellow to red (most abundant). b) Two-dimensional nMDS plot showing localization of Seal Pool (blue triangle), Atlas Pool (green triangle), Brown Lagoon (pink circle), Winston Lagoon (red diamond) and Atlas Cove (aqua square) samples. Clusters with similarity greater than 40 % (green line), 60 % (blue dashed line) and 80 % (aqua line) are marked.

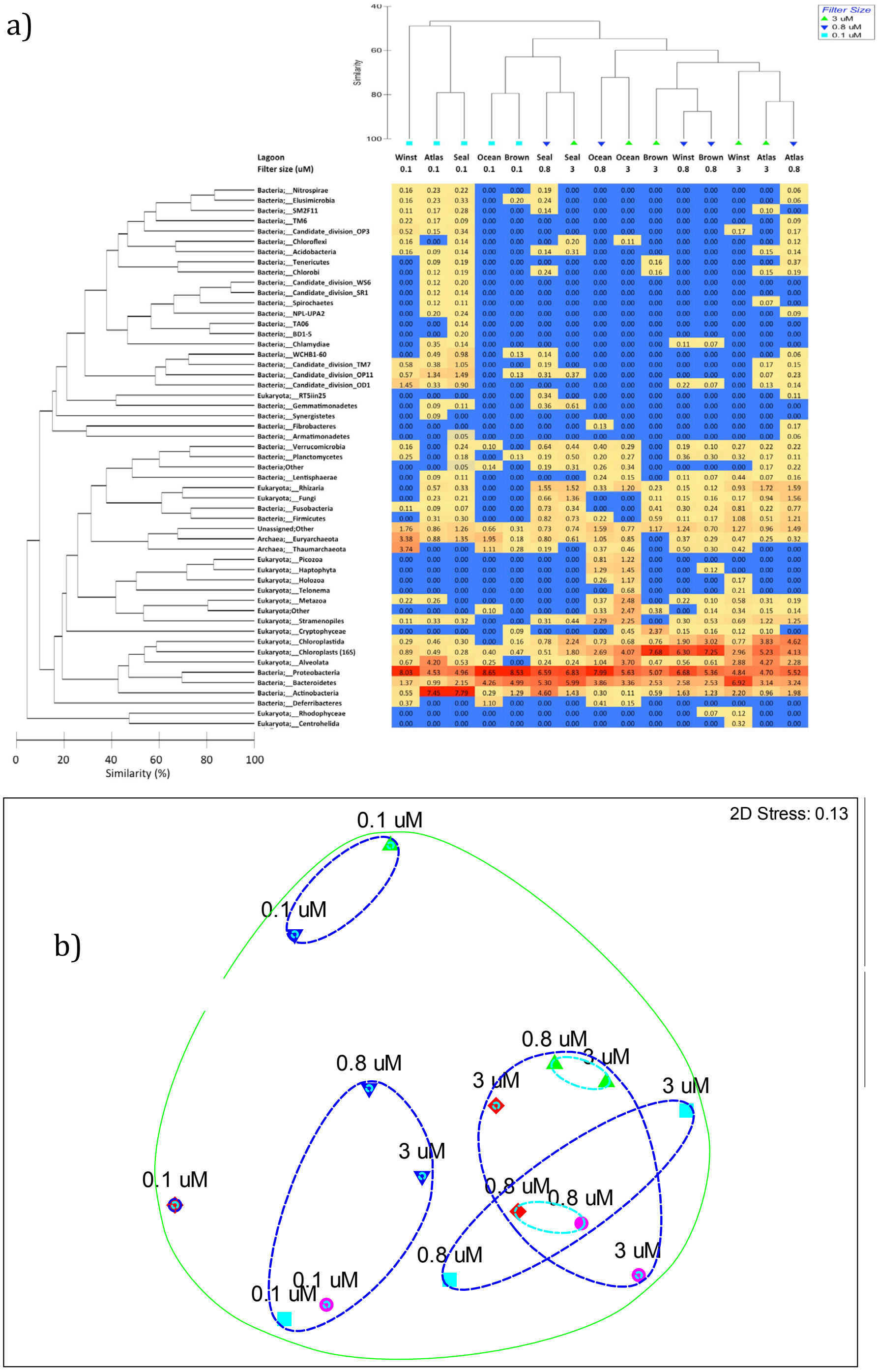

Table S1. OTUs present in all locations.

OTU ID	Total seq	Seal Pool	Atlas Pool	Brown Lagoon	Winston Lagoon	Atlas Cove	Taxon
denovo1428	5360	181	341	4125	712	1	*Betaproteobacteria*
denovo2631	2888	3	2752	1	30	102	Chloroplast
denovo1904	1905	75	216	1368	181	65	*Betaproteobacteria*
denovo1322	692	4	2	487	197	2	*Actinobacteria*
denovo179	95	17	2	29	44	3	*Gammaproteobacteria*
denovo1094	46	16	17	11	1	1	*Actinobacteria*

Table S2. Difference in community composition as detected by pairwise comparisons using ANOSIM and SIMPER. ANOSIM generates a test statistic R indicative of the difference between groups where a value of 0 indicates the null hypothesis (no difference between groups) and 1 indicates all similarities within groups are less than any similarity between groups. R values > 0.50 indicate well separated samples, R > 0.30 are considered overlapping and R > 0.2 as barely separable at all, with p-values considered significant if p = < 0.01.

Pairwise tests	ANOSIM R Statistic	SIMPER av. dissimilarity (%)
Atlas Pool, Seal Pool	0.111	60
Atlas Pool, Atlas Cove	1	92
Atlas Pool, Winston Lagoon	0.963	85
Atlas Pool, Brown Lagoon	1	82
Seal Pool, Atlas Cove	1	92
Seal Pool, Winston Lagoon	1	85
Seal Pool, Brown Lagoon	1	83
Atlas Cove, Winston Lagoon	0.519	69
Atlas Cove, Brown Lagoon	1	84
Winston Lagoon, Brown Lagoon	0.222	66

Table S3. SIMPER one-way analysis data sets.

SIMPER
Similarity Percentages - species contributions

One-Way Analysis

Data worksheet
Name: Square Root Transform
Data type: Abundance
Sample selection: All
Variable selection: All

Parameters
Resemblance: S17 Bray Curtis similarity
Cut off for low contributions: 80.00%

Group Atlas Pool

Average similarity: 46.56

Species	Av.Abund	Av.Sim	Sim/SD	Contrib%	Cum.%
Eukaryota;__Alveolata;__Ciliophora;__Spirotrichea;__Hypotrichia;Other	3.49	4.5	1.94	9.66	9.66
Bacteria;__Cyanobacteria;__Chloroplast;__Chloroplast;__Chloroplast;__Chloroplast	3.27	2.42	0.85	5.2	14.86
Bacteria;__Proteobacteria;__Betaproteobacteria;__Burkholderiales;__Comamonadaceae;Other	2.66	2.31	1.18	4.96	19.83
Bacteria;__Bacteroidetes;__Flavobacteria;__Flavobacteriales;__Flavobacteriaceae;__Flavobacterium	2.2	2.31	1.68	4.96	24.78
Eukaryota;__Chloroplastida;__Chlorophyceae;__Chlamydomonas;__;__	2.25	1.83	0.9	3.92	28.7
Unassigned;Other;Other;Other;Other;Other	1.1	1.38	13.38	2.95	31.66
Bacteria;__Actinobacteria;__Actinobacteria;__Micrococcales;__Microbacteriaceae;Other	1.99	1.35	1.94	2.89	34.55
Eukaryota;__Chloroplastida;__Chlorophyceae;Other;Other;Other	1.85	1.25	0.78	2.68	37.23
Bacteria;__Proteobacteria;__Betaproteobacteria;__Burkholderiales;__Alcaligenaceae;__Achromobacter	1.09	1.2	1.62	2.57	39.8
Bacteria;__Proteobacteria;__Betaproteobacteria;__Burkholderiales;__Comamonadaceae;__Polaromonas	1.23	1.15	1.34	2.46	42.27
Bacteria;__Actinobacteria;__Actinobacteria;__Frankiales;__Sporichthyaceae;__hgcI_clade	2.32	1.08	3.11	2.31	44.58

Bacteria;__Proteobacteria;__Betaproteobacteria;__Burkholderiales;__Burkholderiaceae;__Polynucleobacter	1.75	1.05	2.39	2.27	46.84
Eukaryota;__Rhizaria;__Cercozoa;__Endomyxa;__Novel Clade 10;__	1.09	1.03	1.8	2.21	49.06
Bacteria;__Proteobacteria;__Alphaproteobacteria;__Rhodobacterales;__Rhodobacteraceae;Other	1.03	0.98	1.38	2.11	51.17
Bacteria;__Proteobacteria;__Betaproteobacteria;__Burkholderiales;__Comamonadaceae;__Simplicispira	0.91	0.8	1.25	1.71	52.88
Eukaryota;__Fungi;__Chytridiomycota;__;__;__	0.86	0.66	1.35	1.42	54.3
Bacteria;__Proteobacteria;__Betaproteobacteria;Other;Other;Other	0.64	0.64	2.63	1.38	55.68
Bacteria;__Proteobacteria;__Betaproteobacteria;__Burkholderiales;__Alcaligenaceae;Other	0.57	0.59	1.54	1.26	56.94
Eukaryota;__Rhizaria;__Cercozoa;__Endomyxa;__Phytomyxea;__Spongospora	0.59	0.58	13.24	1.25	58.2
Bacteria;__Actinobacteria;__Actinobacteria;__PeM15;__f;__g	0.79	0.54	1.33	1.17	59.36
Bacteria;__Proteobacteria;__Betaproteobacteria;__Burkholderiales;__Comamonadaceae;__Paucibacter	0.61	0.54	1.07	1.15	60.52
Bacteria;__Actinobacteria;__Actinobacteria;__Frankiales;__Sporichthyaceae;Other	1.02	0.5	3.96	1.08	61.59
Bacteria;__Proteobacteria;__Deltaproteobacteria;__Myxococcales;__0319-6G20;__g	0.41	0.48	4.95	1.04	62.63
Bacteria;__Actinobacteria;__Actinobacteria;__Micrococcales;__Microbacteriaceae;__Candidatus_Aquiluna	0.53	0.45	2.56	0.96	63.59
Bacteria;__Proteobacteria;__Betaproteobacteria;__Burkholderiales;__Comamonadaceae;__Albidiferax	0.3	0.44	10.77	0.94	64.53
Eukaryota;__Chloroplastida;__Trebouxiophyceae;__;__;__	0.4	0.39	1.21	0.83	65.36
Eukaryota;__Stramenopiles;__Chrysophyceae;__Chromulinales;__Phaeoplaca;__	0.26	0.38	8.06	0.82	66.18
Eukaryota;__Stramenopiles;__Diatomea;__Bacillariophytina;__Mediophyceae;Other	0.58	0.37	0.58	0.8	66.98
Bacteria;__Proteobacteria;__Gammaproteobacteria;__Xanthomonadales;__Xanthomonadaceae;__Arenimonas	0.38	0.36	1.8	0.78	67.76
Bacteria;__Proteobacteria;__Gammaproteobacteria;__Pseudomonadales;__Moraxellaceae;__Psychrobacter	0.5	0.35	0.98	0.75	68.51
Eukaryota;__Stramenopiles;__T53A1;__;__;__	0.37	0.34	1.41	0.73	69.24
Bacteria;__Actinobacteria;__Actinobacteria;__Micrococcales;__Microbacteriaceae;__Candidatus_Limnoluna	0.45	0.32	2.89	0.69	69.94
Eukaryota;__Metazoa;__Arthropoda;__Maxillopoda;__;__	0.24	0.32	4.46	0.69	70.63
Eukaryota;__Stramenopiles;__Bicosoecida;__Siluaniidae;__Siluania;__	0.36	0.32	1.05	0.68	71.31
Bacteria;__Proteobacteria;__Alphaproteobacteria;__Rickettsiales;__mitochondria;__g	0.37	0.31	1.07	0.67	71.97
Eukaryota;__Alveolata;__Ciliophora;__Conthreep;__Prostomatea;__Cryptocaryon	0.26	0.31	2.96	0.66	72.63
Bacteria;__Proteobacteria;__Betaproteobacteria;__Burkholderiales;__Oxalobacteraceae;__Undibacterium	0.36	0.28	1.18	0.6	73.23
Bacteria;__Bacteroidetes;__Sphingobacteriia;__Sphingobacteriales;__NS11-12_marine_group;__g	0.41	0.26	1.26	0.57	73.8
Bacteria;__Proteobacteria;__Betaproteobacteria;__Rhodocyclales;__Rhodocyclaceae;__Dechloromonas	0.23	0.26	10.26	0.55	74.35
Bacteria;__Proteobacteria;__Gammaproteobacteria;__Pseudomonadales;__Moraxellaceae;__Perlucidibaca	0.27	0.26	1.3	0.55	74.9
Bacteria;__Proteobacteria;__Betaproteobacteria;__Burkholderiales;__Comamonadaceae;__g	0.45	0.25	0.58	0.54	75.45
Bacteria;__Proteobacteria;__Deltaproteobacteria;__Syntrophobacterales;__Syntrophaceae;__Smithella	0.17	0.24	7.38	0.52	75.97

Bacteria;__Firmicutes;__Clostridia;__Clostridiales;__Ruminococcaceae;__g	0.35	0.24	3.35	0.52	76.49
Bacteria;__Candidate_division_TM7;__c;__o;__f;__g	0.23	0.24	5.6	0.52	77.01
Bacteria;__Proteobacteria;__Gammaproteobacteria;__Xanthomonadales;__Sinobacteraceae;__g	0.17	0.23	8.06	0.5	77.51
Bacteria;__Proteobacteria;__Alphaproteobacteria;__Rhodobacterales;__Rhodobacteraceae;__Rhodobacter	0.33	0.23	0.58	0.48	77.99
Bacteria;__Proteobacteria;__Deltaproteobacteria;__Bdellovibrionales;__Bacteriovoracaceae;__Bacteriovorax	0.24	0.22	2.21	0.48	78.47
Bacteria;__Candidate_division_OD1;__c;__o;__f;__g	0.2	0.2	8.72	0.44	78.91
Bacteria;__Fusobacteria;__Fusobacteria;__Fusobacteriales;__Fusobacteriaceae;__Fusobacterium	0.36	0.2	2.06	0.43	79.33
Archaea;__Euryarchaeota;__Methanomicrobia;__Methanomicrobiales;__Methanoregulaceae;__Methanoregula	0.19	0.2	1.4	0.42	79.76
Eukaryota;__Alveolata;__Protalveolata;__Colpodellida;Other;Other	0.33	0.2	0.58	0.42	80.18

Group Seal Pool

Average similarity: 45.67

Species	Av.Abund	Av.Sim	Sim/SD	Contrib%	Cum.%
Bacteria;__Actinobacteria;__Actinobacteria;__PeM15;__f;__g	3.86	3.07	1.21	6.71	6.71
Bacteria;__Proteobacteria;__Betaproteobacteria;__Burkholderiales;__Comamonadaceae;Other	2.78	2.8	1.63	6.13	12.85
Bacteria;__Bacteroidetes;__Flavobacteria;__Flavobacteriales;__Flavobacteriaceae;__Flavobacterium	3.15	2.72	1.29	5.96	18.81
Bacteria;__Proteobacteria;__Betaproteobacteria;__Burkholderiales;__Comamonadaceae;__Polaromonas	2	1.82	2.2	3.98	22.79
Bacteria;__Bacteroidetes;__Sphingobacteriia;__Sphingobacteriales;__Chitinophagaceae;__Sediminibacterium	1.15	1.16	10.95	2.54	25.33
Bacteria;__Proteobacteria;__Betaproteobacteria;__Burkholderiales;__Burkholderiaceae;__Polynucleobacter	2.03	1.15	1.52	2.51	27.84
Bacteria;__Proteobacteria;__Alphaproteobacteria;__Rhodobacterales;__Rhodobacteraceae;Other	1.79	1.05	0.7	2.29	30.13
Unassigned;Other;Other;Other;Other;Other	0.91	1.04	22.06	2.27	32.4
Bacteria;__Bacteroidetes;__Sphingobacteriia;__Sphingobacteriales;__Sphingobacteriaceae;__Pedobacter	1.06	0.97	1.25	2.13	34.53
Bacteria;__Bacteroidetes;__Flavobacteria;__Flavobacteriales;__Cryomorphaceae;__Fluviicola	0.9	0.96	2.13	2.11	36.64
Bacteria;__Proteobacteria;__Betaproteobacteria;__Burkholderiales;__Comamonadaceae;__Simplicispira	1.25	0.95	0.99	2.08	38.72
Eukaryota;__Rhizaria;__Cercozoa;__Thecofilosea;Other;Other	1.1	0.93	0.98	2.04	40.77
Archaea;__Euryarchaeota;__Methanomicrobia;__Methanomicrobiales;__Methanoregulaceae;__Methanoregula	0.61	0.83	22.02	1.82	42.59

Bacteria;__Actinobacteria;__Actinobacteria;__Micrococcales;__Microbacteriaceae;Other	1.18	0.78	1.34	1.71	44.29
Bacteria;__Proteobacteria;__Gammaproteobacteria;__Pseudomonadales;__Moraxellaceae;__Perlucidibaca	0.76	0.76	6.69	1.67	45.96
Bacteria;__Bacteroidetes;__Sphingobacteriia;__Sphingobacteriales;__NS11-12_marine_group;__g	1.14	0.72	0.98	1.57	47.54
Bacteria;__Proteobacteria;__Betaproteobacteria;__Burkholderiales;__Comamonadaceae;__g	1.15	0.71	1.1	1.56	49.1
Bacteria;__Proteobacteria;__Betaproteobacteria;Other;Other;Other	0.54	0.69	25.44	1.52	50.62
Bacteria;__Proteobacteria;__Betaproteobacteria;__Burkholderiales;__Alcaligenaceae;__Achromobacter	0.67	0.69	1.96	1.5	52.12
Bacteria;__Actinobacteria;Other;Other;Other;Other	0.78	0.67	5.3	1.46	53.58
Bacteria;__Proteobacteria;__Betaproteobacteria;__Burkholderiales;__Alcaligenaceae;Other	0.64	0.64	4.36	1.41	54.99
Bacteria;__Proteobacteria;__Deltaproteobacteria;__Bdellovibrionales;__Bacteriovoracaceae;__Bacteriovorax	0.5	0.63	5.36	1.37	56.36
Bacteria;__Proteobacteria;__Alphaproteobacteria;__Sphingomonadales;__Sphingomonadaceae;__Novosphingobium	0.68	0.61	2.57	1.33	57.69
Bacteria;__Bacteroidetes;__Sphingobacteriia;__Sphingobacteriales;__Chitinophagaceae;__Ferruginibacter	0.86	0.6	0.9	1.32	59
Bacteria;__Actinobacteria;__Actinobacteria;__Frankiales;__Sporichthyaceae;__hgcI_clade	1.22	0.58	1.11	1.28	60.28
Bacteria;__Proteobacteria;__Betaproteobacteria;__Burkholderiales;__Comamonadaceae;__Albidiferax	0.77	0.57	0.71	1.25	61.54
Bacteria;__Proteobacteria;__Betaproteobacteria;__Burkholderiales;Other;Other	0.53	0.54	1.82	1.17	62.71
Bacteria;__Proteobacteria;__Betaproteobacteria;__Burkholderiales;__Comamonadaceae;__Paucibacter	0.97	0.51	0.91	1.13	63.83
Bacteria;__Actinobacteria;__Actinobacteria;__Micrococcales;__Microbacteriaceae;__Candidatus_Aquiluna	0.62	0.49	1.92	1.07	64.91
Eukaryota;__Chloroplastida;__Chlorophyceae;__Desmodesmus;__;__	1.02	0.48	1.05	1.05	65.96
Bacteria;__Candidate_division_OP11;__c;__o;__f;__g	0.72	0.47	7.02	1.02	66.98
Bacteria;__Cyanobacteria;__Chloroplast;__Chloroplast;__Chloroplast;__Chloroplast	0.84	0.47	2.3	1.02	68
Bacteria;__Actinobacteria;__Actinobacteria;__Frankiales;__Sporichthyaceae;Other	1.02	0.47	1	1.02	69.02
Bacteria;__Proteobacteria;__Gammaproteobacteria;__Pseudomonadales;__Moraxellaceae;__Psychrobacter	0.53	0.46	1.21	1.01	70.02
Bacteria;__Proteobacteria;__Alphaproteobacteria;__Rhodospirillales;__Acetobacteraceae;__Roseococcus	0.71	0.46	0.69	1	71.02
Bacteria;__Actinobacteria;__Acidimicrobiia;__Acidimicrobiales;__uncultured;__g	0.41	0.43	7.03	0.94	71.96
Bacteria;__Bacteroidetes;__Sphingobacteriia;__Sphingobacteriales;__Chitinophagaceae;__g	0.71	0.4	1.09	0.87	72.83
Bacteria;__Proteobacteria;__Gammaproteobacteria;__Oceanospirillales;__oc58;__g	0.39	0.36	1.44	0.79	73.62
Bacteria;__Bacteroidetes;__vadinHA17;__o;__f;__g	0.34	0.33	4.27	0.72	74.34
Eukaryota;__Fungi;__Chytridiomycota;__;__;__	0.63	0.32	0.76	0.69	75.03
Bacteria;__Proteobacteria;__Betaproteobacteria;__Burkholderiales;__Oxalobacteraceae;__Undibacterium	0.28	0.3	3.96	0.65	75.68
Bacteria;__Proteobacteria;__Deltaproteobacteria;__Myxococcales;__0319-6G20;__g	0.36	0.28	2	0.61	76.28
Bacteria;__Proteobacteria;Other;Other;Other;Other	0.24	0.27	40.49	0.59	76.88
Bacteria;__Proteobacteria;__Betaproteobacteria;__Burkholderiales;__Comamonadaceae;__Rhizobacter	0.28	0.27	2.93	0.59	77.47

Bacteria;__Gemmatimonadetes;__Gemmatimonadetes;__Gemmatimonadales;__Gemmatimonadaceae;__Gemmatimonas	0.35	0.27	1.35	0.58	78.05
Eukaryota;__Fungi;__LKM11;__;__;__	0.26	0.26	2.6	0.57	78.62
Bacteria;__Bacteroidetes;__Sphingobacteriia;__Sphingobacteriales;__Saprospiraceae;__g	0.76	0.26	0.99	0.57	79.19
Eukaryota;__Stramenopiles;__Chrysophyceae;Other;Other;Other	0.22	0.25	2.99	0.54	79.72
Bacteria;__Verrucomicrobia;__Opitutae;__Opitutales;__Opitutaceae;__Opitutus	0.36	0.24	0.84	0.53	80.25

Group Atlas Cove

Average similarity: 53.06

Species	Av.Abund	Av.Sim	Sim/SD	Contrib%	Cum.%
Bacteria;__Proteobacteria;__Gammaproteobacteria;Other;Other;Other	3.15	3.59	6.96	6.76	6.76
Bacteria;__Proteobacteria;__Gammaproteobacteria;__Oceanospirillales;__ZD0405;__g	3.19	3.37	2.33	6.35	13.12
Bacteria;__Bacteroidetes;__Flavobacteria;__Flavobacteriales;__Flavobacteriaceae;__NS2b_marine_group	1.92	2.58	4.38	4.86	17.98
Bacteria;__Proteobacteria;__Alphaproteobacteria;__SAR11_clade;__Surface_1;__g	2.82	2.35	4.38	4.43	22.4
Bacteria;__Proteobacteria;__Gammaproteobacteria;__Oceanospirillales;__SAR86_clade;__g	1.99	2.3	2.34	4.34	26.74
Bacteria;__Proteobacteria;__Gammaproteobacteria;__Alteromonadales;__Alteromonadaceae;__SAR92_clade	2.09	2.25	7.47	4.24	30.97
Bacteria;__Bacteroidetes;__Flavobacteria;__Flavobacteriales;__Flavobacteriaceae;__Polaribacter	1.7	2.16	9.39	4.07	35.04
Bacteria;__Bacteroidetes;__Flavobacteria;__Flavobacteriales;__NS9_marine_group;__g	1.54	2.11	14.82	3.98	39.02
Bacteria;__Proteobacteria;__Alphaproteobacteria;__Rhodobacterales;__Rhodobacteraceae;__Roseobacter_clade_DC5-80-3_lineage	1.59	1.92	2.09	3.62	42.64
Bacteria;__Cyanobacteria;__Chloroplast;__Chloroplast;__Chloroplast;__Chloroplast	2.38	1.59	0.95	3	45.64
Archaea;__Euryarchaeota;__Thermoplasmata;__Thermoplasmatales;__Marine_Group_II;__g	1.28	1.38	4.02	2.6	48.23
Bacteria;__Bacteroidetes;__Flavobacteria;__Flavobacteriales;__Flavobacteriaceae;__NS4_marine_group	1.31	1.34	1.74	2.53	50.76
Unassigned;Other;Other;Other;Other;Other	1.01	1.04	14.51	1.95	52.72
Bacteria;__Bacteroidetes;__Flavobacteria;__Flavobacteriales;__Flavobacteriaceae;Other	1.01	0.99	6.13	1.87	54.59
Bacteria;__Proteobacteria;__Alphaproteobacteria;__SAR11_clade;Other;Other	0.9	0.87	4.48	1.64	56.23
Bacteria;__Proteobacteria;__Alphaproteobacteria;__Rhodobacterales;__Rhodobacteraceae;__g	0.85	0.86	4.05	1.63	57.86
Bacteria;__Proteobacteria;__Gammaproteobacteria;__Oceanospirillales;__Oceanospirillaceae;Other	0.68	0.85	14.94	1.6	59.45
Bacteria;__Proteobacteria;__Gammaproteobacteria;__Alteromonadales;__Pseudoalteromonadaceae;__Pseudoalteromonas	1.18	0.8	0.81	1.5	60.96
Bacteria;__Bacteroidetes;__Flavobacteria;__Flavobacteriales;Other;Other	0.84	0.74	3.1	1.4	62.36

Bacteria;__Proteobacteria;__Alphaproteobacteria;__Rickettsiales;__SAR116_clade;__g	0.57	0.71	1.8	1.33	63.69
Bacteria;__Proteobacteria;__Gammaproteobacteria;__Alteromonadales;__Alteromonadaceae;__Alteromonas	1.1	0.69	0.58	1.3	64.98
Eukaryota;__Stramenopiles;__MAST-1;__MAST-1 B;__;__	1	0.61	0.58	1.16	66.14
Bacteria;__Proteobacteria;__Gammaproteobacteria;__Oceanospirillales;__Alcanivoracaceae;__Alcanivorax	0.67	0.6	2.06	1.12	67.26
Archaea;__Thaumarchaeota;__Marine_Group_I;__o;__f;__g	0.65	0.59	5.73	1.12	68.38
Bacteria;__Proteobacteria;__Gammaproteobacteria;__Oceanospirillales;__OM182_clade;__g	0.55	0.54	8.73	1.02	69.4
Bacteria;__Bacteroidetes;__Flavobacteria;__Flavobacteriales;__Flavobacteriaceae;__NS3a_marine_group	0.38	0.54	7.23	1.02	70.42
Bacteria;__Bacteroidetes;__Flavobacteria;__Flavobacteriales;__Cryomorphaceae;__Owenweeksia	0.47	0.54	2.25	1.01	71.44
Eukaryota;__Haptophyta;__Prymnesiophyceae;Other;Other;Other	0.86	0.53	0.58	1	72.44
Bacteria;__Proteobacteria;__Betaproteobacteria;Other;Other;Other	0.45	0.53	4.15	0.99	73.43
Bacteria;__Proteobacteria;__Gammaproteobacteria;__Alteromonadales;__Alteromonadaceae;Other	0.45	0.49	4.32	0.93	74.36
Bacteria;__Proteobacteria;__Alphaproteobacteria;__SAR11_clade;__Surface_4;__g	0.49	0.47	5.28	0.89	75.25
Bacteria;__Bacteroidetes;__Flavobacteria;__Flavobacteriales;__Flavobacteriaceae;__NS5_marine_group	0.6	0.46	2.84	0.88	76.13
Bacteria;__Proteobacteria;__Alphaproteobacteria;__Rhodobacterales;__Rhodobacteraceae;__Sulfitobacter	0.73	0.46	0.58	0.87	77
Bacteria;__Bacteroidetes;__Flavobacteria;__Flavobacteriales;__Flavobacteriaceae;__g	0.39	0.45	9.7	0.85	77.84
Bacteria;__Proteobacteria;__Alphaproteobacteria;Other;Other;Other	0.42	0.41	3.03	0.77	78.61
Bacteria;__Proteobacteria;__Gammaproteobacteria;__Alteromonadales;__Colwelliaceae;__Colwellia	0.61	0.38	1.46	0.71	79.33
Eukaryota;__Stramenopiles;__MAST-3;__;__;__	0.61	0.37	0.58	0.7	80.03

Group Winston Lagoon

Average similarity: 31.66

Species	Av.Abund	Av.Sim	Sim/SD	Contrib%	Cum.%
Bacteria;__Cyanobacteria;__Chloroplast;__Chloroplast;__Chloroplast;__Chloroplast	3.37	2.45	1.36	7.75	7.75
Unassigned;Other;Other;Other;Other;Other	1.43	2.02	9.2	6.38	14.14
Bacteria;__Proteobacteria;__Alphaproteobacteria;__Rhodobacterales;__Rhodobacteraceae;Other	1.87	1.62	0.85	5.11	19.24
Bacteria;__Bacteroidetes;__Flavobacteria;__Flavobacteriales;__Flavobacteriaceae;Other	2.34	0.96	5.25	3.04	22.29
Bacteria;__Proteobacteria;__Gammaproteobacteria;__Alteromonadales;__Pseudoalteromonadaceae;__g	1.15	0.89	1.17	2.8	25.09
Bacteria;__Proteobacteria;__Gammaproteobacteria;__Alteromonadales;__Colwelliaceae;__Colwellia	0.95	0.77	1.38	2.43	27.51
Bacteria;__Proteobacteria;__Gammaproteobacteria;__Oceanospirillales;__SAR86_clade;__g	1.39	0.75	1.86	2.36	29.87

Bacteria;__Proteobacteria;__Alphaproteobacteria;__Rhodobacterales;__Rhodobacteraceae;__Roseobacter_clade_DC5-80-3_lineage	0.55	0.73	2.31	2.3	32.17
Archaea;__Thaumarchaeota;__Marine_Group_I;__o;__f;__g	1.55	0.73	4.32	2.3	34.47
Bacteria;__Proteobacteria;__Gammaproteobacteria;__Oceanospirillales;__ZD0405;__g	1.34	0.71	1.77	2.25	36.72
Bacteria;__Bacteroidetes;__Flavobacteria;__Flavobacteriales;__Flavobacteriaceae;__NS2b_marine_group	0.42	0.66	9.36	2.09	38.81
Bacteria;__Proteobacteria;__Alphaproteobacteria;__Rhodobacterales;__Rhodobacteraceae;__g	0.89	0.57	1.05	1.81	40.62
Bacteria;__Bacteroidetes;__Flavobacteria;__Flavobacteriales;__Flavobacteriaceae;__Polaribacter	0.91	0.52	1.48	1.64	42.25
Bacteria;__Proteobacteria;__Deltaproteobacteria;__Bdellovibrionales;__Bacteriovoracaceae;__Peredibacter	0.59	0.51	1.94	1.62	43.88
Bacteria;__Proteobacteria;__Gammaproteobacteria;Other;Other;Other	0.38	0.48	1.68	1.52	45.39
Bacteria;__Bacteroidetes;__Flavobacteria;__Flavobacteriales;__Flavobacteriaceae;__NS4_marine_group	0.36	0.47	2.57	1.48	46.87
Archaea;__Euryarchaeota;__Thermoplasmata;__Thermoplasmatales;__Marine_Group_II;__g	0.37	0.46	9.68	1.47	48.34
Bacteria;__Proteobacteria;__Alphaproteobacteria;__SAR11_clade;__Surface_1;__g	2.12	0.46	1.8	1.46	49.81
Bacteria;__Proteobacteria;__Gammaproteobacteria;__Alteromonadales;__Alteromonadaceae;__SAR92_clade	0.35	0.46	2.85	1.44	51.25
Bacteria;__Proteobacteria;__Betaproteobacteria;Other;Other;Other	0.68	0.44	0.99	1.37	52.62
Bacteria;__Actinobacteria;__Actinobacteria;__Corynebacteriales;__Nocardiaceae;__Rhodococcus	0.96	0.43	1.01	1.35	53.97
Bacteria;__Proteobacteria;__Alphaproteobacteria;__Sphingomonadales;__Sphingomonadaceae;__Sphingopyxis	1.75	0.42	1.47	1.34	55.32
Bacteria;__Proteobacteria;__Gammaproteobacteria;__Oceanospirillales;__Oceanospirillaceae;Other	0.36	0.42	3.99	1.32	56.64
Eukaryota;__Chloroplastida;__Mamiellophyceae;__Micromonas;__;__	0.8	0.41	5.31	1.29	57.93
Archaea;__Euryarchaeota;__Halobacteria;__Halobacteriales;__Deep_Sea_Hydrothermal_Vent_Gp_6(DHVEG-6);__Candidatus_Parvarchaeum	1.11	0.4	11.4	1.27	59.2
Bacteria;__Bacteroidetes;__Flavobacteria;__Flavobacteriales;__Cryomorphaceae;__Owenweeksia	0.39	0.4	1.58	1.27	60.47
Bacteria;__Bacteroidetes;__Flavobacteria;__Flavobacteriales;__Flavobacteriaceae;__NS3a_marine_group	0.9	0.4	6.15	1.25	61.72
Bacteria;__Proteobacteria;__Gammaproteobacteria;__Pseudomonadales;__Pseudomonadaceae;__Pseudomonas	0.3	0.36	11.4	1.14	62.85
Bacteria;__Proteobacteria;__Gammaproteobacteria;__Alteromonadales;__Shewanellaceae;__Shewanella	0.28	0.35	2.25	1.09	63.95
Bacteria;__Proteobacteria;__Gammaproteobacteria;__Enterobacteriales;__Enterobacteriaceae;Other	0.37	0.33	2.57	1.05	65
Bacteria;__Bacteroidetes;__Flavobacteria;__Flavobacteriales;__NS9_marine_group;__g	0.4	0.32	3	1	66
Bacteria;__Bacteroidetes;__Cytophagia;__Cytophagales;__Flammeovirgaceae;__Reichenbachiella	0.29	0.3	9.42	0.96	66.96
Bacteria;__Proteobacteria;__Gammaproteobacteria;__Alteromonadales;__Alteromonadaceae;Other	0.25	0.3	9.42	0.96	67.92
Eukaryota;__Alveolata;__Ciliophora;__Spirotrichea;__Oligotrichia;__uncultured	0.26	0.3	9.42	0.96	68.87
Bacteria;__Fusobacteria;__Fusobacteria;__Fusobacteriales;__Fusobacteriaceae;__Fusobacterium	0.36	0.28	1.68	0.88	69.75
Eukaryota;__Alveolata;__Ciliophora;__Spirotrichea;__Hypotrichia;Other	0.3	0.27	1.68	0.87	70.62
Eukaryota;__Alveolata;__Ciliophora;__Spirotrichea;__Oligotrichia;__Strombidium	0.27	0.27	1.68	0.87	71.49

Bacteria;__Proteobacteria;__Alphaproteobacteria;__Rhodobacterales;__Rhodobacteraceae;__Loktanella	0.36	0.27	0.58	0.85	72.34
Eukaryota;__Alveolata;__Ciliophora;__Conthreep;__Phyllopharyngea;__AT1-2	0.75	0.26	9.66	0.84	73.17
Bacteria;__Bacteroidetes;__Flavobacteria;__Flavobacteriales;__Flavobacteriaceae;__NS5_marine_group	0.32	0.25	2.07	0.8	73.97
Bacteria;__Proteobacteria;__Gammaproteobacteria;__Alteromonadales;Other;Other	0.24	0.25	2.23	0.78	74.75
Bacteria;__Bacteroidetes;__Flavobacteria;__Flavobacteriales;__Cryomorphaceae;__Fluviicola	0.31	0.23	2.67	0.73	75.48
Bacteria;__Bacteroidetes;__Sphingobacteriia;__Sphingobacteriales;__Saprospiraceae;__Lewinella	0.48	0.23	2.67	0.73	76.21
Bacteria;__Proteobacteria;__Alphaproteobacteria;__Kordiimonadales;__Kordiimonadaceae;__Kordiimonas	0.28	0.23	2.67	0.73	76.95
Eukaryota;__Alveolata;__Ciliophora;__Spirotrichea;__Hypotrichia;__	0.31	0.23	2.67	0.72	77.67
Eukaryota;__Metazoa;__Arthropoda;__Maxillopoda;__;__	0.25	0.23	2.67	0.72	78.4
Bacteria;__Planctomycetes;__BD7-11;__o;__f;__g	0.21	0.23	2.79	0.72	79.12
Eukaryota;__Alveolata;__Ciliophora;__Spirotrichea;__Euplotia;__Aspidisca	0.26	0.21	3.51	0.68	79.8
Bacteria;__Verrucomicrobia;__Opitutae;__Puniceicoccales;__Puniceicoccaceae;__Lentimonas	0.15	0.2	5.95	0.62	80.42

Group Brown Lagoon

Average similarity: 44.47

Species	Av.Abund	Av.Sim	Sim/SD	Contrib%	Cum.%
Bacteria;__Proteobacteria;__Alphaproteobacteria;__Sphingomonadales;__Sphingomonadaceae;__Sphingopyxis	4.06	7.05	4.12	15.85	15.85
Bacteria;__Cyanobacteria;__Chloroplast;__Chloroplast;__Chloroplast;__Chloroplast	5.14	6.63	0.71	14.91	30.76
Bacteria;__Proteobacteria;__Betaproteobacteria;__Burkholderiales;__Comamonadaceae;Other	3.08	5.98	3.82	13.45	44.21
Bacteria;__Bacteroidetes;__Flavobacteria;__Flavobacteriales;__Flavobacteriaceae;__NS3a_marine_group	3.07	5.48	11.81	12.31	56.53
Bacteria;__Proteobacteria;__Alphaproteobacteria;__Rhodobacterales;__Rhodobacteraceae;Other	2.06	2.39	1.54	5.37	61.9
Bacteria;__Actinobacteria;__Acidimicrobiia;__Acidimicrobiales;__Acidimicrobiaceae;__g	0.99	1.98	2.46	4.45	66.35
Bacteria;__Bacteroidetes;__Flavobacteria;__Flavobacteriales;__Flavobacteriaceae;__Polaribacter	0.68	1.51	10.31	3.39	69.74
Bacteria;__Proteobacteria;__Alphaproteobacteria;__Rhodobacterales;__Rhodobacteraceae;__Loktanella	0.49	1.18	4.82	2.65	72.38
Unassigned;Other;Other;Other;Other;Other	0.73	1.11	2.27	2.49	74.88
Bacteria;__Proteobacteria;__Alphaproteobacteria;__Sphingomonadales;__Erythrobacteraceae;__Altererythrobacter	0.78	1.08	7.11	2.43	77.3
Bacteria;__Proteobacteria;__Betaproteobacteria;Other;Other;Other	1.33	0.84	4.36	1.88	79.18
Eukaryota;__Chloroplastida;__Mamiellophyceae;__Micromonas;__;__	1.27	0.79	1.21	1.78	80.96

Groups Atlas Pool & Seal Pool

Average dissimilarity = 60.35

Species	Atlas Pool Av.Abund	Seal Pool Av.Abund	Av.Diss	Diss/SD	Contrib%	Cum.%
Eukaryota;__Alveolata;__Ciliophora;__Spirotrichea;__Hypotrichia;Other	3.49	0.14	2.55	2.8	4.22	4.22
Bacteria;__Actinobacteria;__Actinobacteria;__PeM15;__f;__g	0.79	3.86	2.36	1.41	3.91	8.13
Bacteria;__Cyanobacteria;__Chloroplast;__Chloroplast;__Chloroplast;__Chloroplast	3.27	0.84	1.96	1.46	3.25	11.38
Bacteria;__Actinobacteria;__Actinobacteria;__Frankiales;__Sporichthyaceae;__hgcI_clade	2.32	1.22	1.58	1	2.61	13.99
Eukaryota;__Chloroplastida;__Chlorophyceae;__Chlamydomonas;__;__	2.25	0.21	1.45	1.52	2.4	16.4
Bacteria;__Bacteroidetes;__Flavobacteria;__Flavobacteriales;__Flavobacteriaceae;__Flavobacterium	2.2	3.15	1.32	1.48	2.18	18.58
Bacteria;__Proteobacteria;__Betaproteobacteria;__Burkholderiales;__Burkholderiaceae;__Polynucleobacter	1.75	2.03	1.24	1.1	2.05	20.63
Eukaryota;__Chloroplastida;__Chlorophyceae;Other;Other;Other	1.85	0.11	1.22	1.38	2.02	22.65
Bacteria;__Actinobacteria;__Actinobacteria;__Micrococcales;__Microbacteriaceae;Other	1.99	1.18	1.12	1.14	1.86	24.51
Bacteria;__Proteobacteria;__Betaproteobacteria;__Burkholderiales;__Comamonadaceae;Other	2.66	2.78	1.04	1.11	1.72	26.23
Bacteria;__Proteobacteria;__Alphaproteobacteria;__Rhodobacterales;__Rhodobacteraceae;Other	1.03	1.79	1.02	1.55	1.69	27.92
Bacteria;__Proteobacteria;__Betaproteobacteria;__Burkholderiales;__Comamonadaceae;__Polaromonas	1.23	2	0.82	1.36	1.36	29.29
Bacteria;__Bacteroidetes;__Sphingobacteriia;__Sphingobacteriales;__Chitinophagaceae;__Sediminibacterium	0.11	1.15	0.77	2.16	1.27	30.56
Bacteria;__Actinobacteria;__Actinobacteria;__Frankiales;__Sporichthyaceae;Other	1.02	1.02	0.75	1.08	1.24	31.8
Eukaryota;__Rhizaria;__Cercozoa;__Thecofilosea;Other;Other	0.08	1.1	0.75	1.59	1.24	33.04
Eukaryota;__Rhizaria;__Cercozoa;__Endomyxa;__Novel Clade 10;__	1.09	0.12	0.69	1.84	1.15	34.2
Bacteria;__Bacteroidetes;__Sphingobacteriia;__Sphingobacteriales;__Sphingobacteriaceae;__Pedobacter	0.17	1.06	0.69	1.58	1.14	35.33
Bacteria;__Proteobacteria;__Betaproteobacteria;__Burkholderiales;__Comamonadaceae;__g	0.45	1.15	0.67	1.26	1.11	36.44
Bacteria;__Bacteroidetes;__Sphingobacteriia;__Sphingobacteriales;__NS11-12_marine_group;__g	0.41	1.14	0.66	1.29	1.09	37.53
Eukaryota;__Chloroplastida;__Chlorophyceae;__Desmodesmus;__;__	0.27	1.02	0.65	1.04	1.07	38.6
Bacteria;__Proteobacteria;__Betaproteobacteria;__Burkholderiales;__Comamonadaceae;__Simplicispira	0.91	1.25	0.58	1.34	0.96	39.56
Bacteria;__Bacteroidetes;__Flavobacteria;__Flavobacteriales;__Cryomorphaceae;__Fluviicola	0.14	0.9	0.57	2.17	0.94	40.5
Bacteria;__Bacteroidetes;__Sphingobacteriia;__Sphingobacteriales;__Chitinophagaceae;__Ferruginibacter	0.17	0.86	0.54	1.41	0.9	41.4
Bacteria;__Bacteroidetes;__Sphingobacteriia;__Sphingobacteriales;__Saprospiraceae;__g	0.07	0.76	0.53	0.93	0.88	42.28
Bacteria;__Proteobacteria;__Betaproteobacteria;__Burkholderiales;__Comamonadaceae;__Paucibacter	0.61	0.97	0.51	1.11	0.85	43.13
Bacteria;__Proteobacteria;__Alphaproteobacteria;__Rhodospirillales;__Acetobacteraceae;__Roseococcus	0.08	0.71	0.48	1.37	0.8	43.93
Bacteria;__Actinobacteria;Other;Other;Other;Other	0.15	0.78	0.48	1.33	0.79	44.72

Bacteria;__Candidate_division_OP11;__c;__o;__f;__g	0.55	0.72	0.47	1.14	0.78	45.5
Bacteria;__Proteobacteria;__Alphaproteobacteria;__Sphingomonadales;__Sphingomonadaceae;__Novosphingobium	0.06	0.68	0.46	1.83	0.76	46.26
Bacteria;__Proteobacteria;__Betaproteobacteria;__Burkholderiales;__Comamonadaceae;__Albidiferax	0.3	0.77	0.45	2.1	0.75	47.01
Eukaryota;__Fungi;__Chytridiomycota;__;__;__	0.86	0.63	0.45	1.46	0.75	47.76
Bacteria;__Proteobacteria;__Betaproteobacteria;__Burkholderiales;__Alcaligenaceae;__Achromobacter	1.09	0.67	0.43	1.76	0.71	48.46
Bacteria;__Bacteroidetes;__Sphingobacteriia;__Sphingobacteriales;__Chitinophagaceae;__g	0.28	0.71	0.42	1.15	0.7	49.16
Eukaryota;__Rhizaria;__Cercozoa;__Endomyxa;__Phytomyxea;__Spongospora	0.59	0	0.42	2.48	0.7	49.86
Eukaryota;__Stramenopiles;__Diatomea;__Bacillariophytina;__Mediophyceae;Other	0.58	0.04	0.4	1.43	0.66	50.52
Bacteria;__Actinobacteria;__Acidimicrobiia;__Acidimicrobiales;__Acidimicrobiaceae;__CL500-29_marine_group	0.03	0.49	0.37	0.98	0.61	51.13
Bacteria;__Proteobacteria;__Gammaproteobacteria;__Pseudomonadales;__Moraxellaceae;__Perlucidibaca	0.27	0.76	0.36	1.48	0.6	51.73
Bacteria;__Bacteroidetes;__Cytophagia;__Cytophagales;__Cytophagaceae;__Leadbetterella	0.14	0.5	0.33	1.36	0.55	52.29
Archaea;__Euryarchaeota;__Methanomicrobia;__Methanomicrobiales;__Methanoregulaceae;__Methanoregula	0.19	0.61	0.32	4.51	0.52	52.81
Archaea;__Euryarchaeota;__Halobacteria;__Halobacteriales;__Deep_Sea_Hydrothermal_Vent_Gp_6(DHVEG-6);__g	0.29	0.41	0.31	1.26	0.51	53.32
Bacteria;__Actinobacteria;__Acidimicrobiia;__Acidimicrobiales;__uncultured;__g	0	0.41	0.31	2.8	0.51	53.83
Bacteria;__Actinobacteria;__Actinobacteria;__Micrococcales;__Microbacteriaceae;__Candidatus_Limnoluna	0.45	0.12	0.3	1.17	0.5	54.33
Bacteria;__WCHB1-60;__c;__o;__f;__g	0.18	0.37	0.3	1.08	0.49	54.82
Bacteria;__Candidate_division_TM7;__c;__o;__f;__g	0.23	0.41	0.29	1.08	0.48	55.31
Bacteria;__Bacteroidetes;__Sphingobacteriia;__Sphingobacteriales;__Chitinophagaceae;__Filimonas	0.26	0.42	0.29	1.16	0.47	55.78
Bacteria;__Actinobacteria;__Actinobacteria;__Micrococcales;__Microbacteriaceae;__Candidatus_Aquiluna	0.53	0.62	0.28	1.22	0.46	56.24
Bacteria;__Candidate_division_OD1;__c;__o;__f;__g	0.2	0.3	0.28	1.33	0.46	56.71
Bacteria;__Proteobacteria;__Betaproteobacteria;__Burkholderiales;Other;Other	0.38	0.53	0.27	1.62	0.45	57.15
Bacteria;__Bacteroidetes;__Sphingobacteriia;__Sphingobacteriales;__WCHB1-69;__g	0.24	0.44	0.27	1.15	0.44	57.59
Eukaryota;__Stramenopiles;__Bicosoecida;__Siluaniidae;__Siluania;__	0.36	0	0.26	1.8	0.43	58.02
Bacteria;__Gemmatimonadetes;__Gemmatimonadetes;__Gemmatimonadales;__Gemmatimonadaceae;__Gemmatimonas	0	0.35	0.26	1.69	0.42	58.45
Bacteria;__Proteobacteria;__Gammaproteobacteria;__Pseudomonadales;__Moraxellaceae;__Psychrobacter	0.5	0.53	0.25	1.3	0.42	58.87
Bacteria;__Proteobacteria;__Alphaproteobacteria;__Rickettsiales;__mitochondria;__g	0.37	0.02	0.25	1.63	0.42	59.29
Unassigned;Other;Other;Other;Other;Other	1.1	0.91	0.25	1.6	0.41	59.7
Bacteria;__Proteobacteria;__Betaproteobacteria;__Burkholderiales;__Comamonadaceae;__Acidovorax	0.13	0.38	0.25	2.12	0.41	60.11

Eukaryota;__Alveolata;__Ciliophora;__Spirotrichea;__Hypotrichia;__Gonostomum	0.29	0	0.24	0.67	0.4	60.51
Eukaryota;__Alveolata;__Protalveolata;__Colpodellida;Other;Other	0.33	0	0.23	1.26	0.39	60.89
Bacteria;__Fusobacteria;__Fusobacteria;__Fusobacteriales;__Fusobacteriaceae;__Fusobacterium	0.36	0.38	0.23	1.23	0.38	61.27
Bacteria;__Verrucomicrobia;__Opitutae;__Opitutales;__Opitutaceae;__Opitutus	0.1	0.36	0.23	1.45	0.37	61.65
Bacteria;__Proteobacteria;__Betaproteobacteria;__Burkholderiales;__Alcaligenaceae;Other	0.57	0.64	0.22	1.65	0.37	62.01
Bacteria;__Bacteroidetes;__Bacteroidia;__Bacteroidales;__Bacteroidaceae;__Bacteroides	0.31	0.21	0.21	1.19	0.35	62.36
Eukaryota;__Chloroplastida;__Trebouxiophyceae;__;__;__	0.4	0.13	0.21	1.57	0.35	62.71
Archaea;__Euryarchaeota;__Halobacteria;__Halobacteriales;__Deep_Sea_Hydrothermal_Vent_Gp_6(DHVEG-6);__Candidatus_Parvarchaeum	0.19	0.3	0.21	1.3	0.35	63.06
Bacteria;__Proteobacteria;__Alphaproteobacteria;__Rhodobacterales;__Rhodobacteraceae;__Rhodobacter	0.33	0.32	0.21	1.21	0.34	63.4
Bacteria;__Proteobacteria;__Betaproteobacteria;Other;Other;Other	0.64	0.54	0.2	1.32	0.33	63.74
Bacteria;__Proteobacteria;__Betaproteobacteria;__Neisseriales;__Neisseriaceae;__g	0	0.27	0.2	2.08	0.33	64.06
Bacteria;__Actinobacteria;__Actinobacteria;__Micrococcales;__Microbacteriaceae;__Candidatus_Planktoluna	0.28	0.04	0.19	1	0.32	64.38
Eukaryota;__Fungi;__LKM11;__;__;__	0	0.26	0.19	2.61	0.32	64.7
Bacteria;__Proteobacteria;__Deltaproteobacteria;__Bdellovibrionales;__Bacteriovoracaceae;__Bacteriovorax	0.24	0.5	0.19	1.72	0.32	65.02
Bacteria;__Proteobacteria;__Alphaproteobacteria;__Sphingomonadales;__Sphingomonadaceae;__Sandarakinorhabdus	0.04	0.27	0.19	1.32	0.32	65.34
Bacteria;__Proteobacteria;__Betaproteobacteria;__Burkholderiales;__Alcaligenaceae;__Pusillimonas	0.2	0.14	0.19	1.11	0.32	65.65
Eukaryota;__Stramenopiles;__Chrysophyceae;__Chromulinales;__Phaeoplaca;__	0.26	0	0.19	8.34	0.32	65.97
Bacteria;__Proteobacteria;__Gammaproteobacteria;__Xanthomonadales;__Xanthomonadaceae;__Arenimonas	0.38	0.15	0.18	1.5	0.31	66.28
Bacteria;__Proteobacteria;__Gammaproteobacteria;__Oceanospirillales;__oc58;__g	0.27	0.39	0.18	1.25	0.3	66.58
Eukaryota;__Metazoa;__Arthropoda;__Maxillopoda;__;__	0.24	0	0.18	4.11	0.3	66.88
Bacteria;__Firmicutes;__Clostridia;__Clostridiales;__Ruminococcaceae;__g	0.35	0.24	0.18	1.34	0.3	67.18
Bacteria;__Proteobacteria;__Betaproteobacteria;__Burkholderiales;__Comamonadaceae;__Hydrogenophaga	0.26	0.06	0.17	1.2	0.29	67.46
Bacteria;__Actinobacteria;__Actinobacteria;__Micrococcales;__Microbacteriaceae;__Alpinimonas	0.19	0.08	0.17	0.87	0.28	67.75
Bacteria;__Proteobacteria;__Betaproteobacteria;__Burkholderiales;__Comamonadaceae;__Rhizobacter	0.06	0.28	0.17	1.72	0.28	68.03
Bacteria;__Proteobacteria;__Betaproteobacteria;__Rhodocyclales;__Rhodocyclaceae;__Dechloromonas	0.23	0	0.17	2.96	0.28	68.3
Bacteria;__Bacteroidetes;__Bacteroidia;__Bacteroidales;__Prevotellaceae;__Prevotella	0.19	0.24	0.16	1.23	0.27	68.57
Bacteria;__Proteobacteria;__Deltaproteobacteria;__Syntrophobacterales;__Syntrophaceae;__Smithella	0.17	0.16	0.16	2.52	0.27	68.85
Eukaryota;__Alveolata;__Ciliophora;__Conthreep;__Oligohymenophorea;Other	0.27	0.17	0.16	1.26	0.26	69.11
Bacteria;__Proteobacteria;__Betaproteobacteria;__Burkholderiales;__Oxalobacteraceae;__Undibacterium	0.36	0.28	0.16	1.74	0.26	69.36

Bacteria;__Proteobacteria;__Gammaproteobacteria;__Alteromonadales;__Alteromonadaceae;__BD1-7_clade	0.18	0.29	0.16	1.3	0.26	69.62
Eukaryota;__Stramenopiles;__T53A1;__;__;__	0.37	0.24	0.15	1.33	0.26	69.88
Bacteria;__Proteobacteria;__Alphaproteobacteria;__Rhodobacterales;__Rhodobacteraceae;__Paracoccus	0.11	0.18	0.15	1.1	0.25	70.13
Bacteria;__Proteobacteria;__Deltaproteobacteria;__Myxococcales;__0319-6G20;__g	0.41	0.36	0.15	1.41	0.25	70.38
Eukaryota;__Chloroplastida;__Chlorophyceae;__;__;__	0.22	0.02	0.15	1.29	0.25	70.63
Bacteria;__Proteobacteria;__Gammaproteobacteria;__Xanthomonadales;__Xanthomonadaceae;Other	0.21	0	0.15	1.14	0.25	70.87
Eukaryota;__Alveolata;__Ciliophora;__Conthreep;__Prostomatea;__Cryptocaryon	0.26	0.08	0.15	2	0.24	71.12
Bacteria;__Proteobacteria;__Betaproteobacteria;__Burkholderiales;__Burkholderiaceae;__Ralstonia	0.23	0.13	0.15	1.24	0.24	71.36
Eukaryota;__Stramenopiles;__Chrysophyceae;Other;Other;Other	0.02	0.22	0.14	2.55	0.24	71.59
Bacteria;__Firmicutes;__Bacilli;__Bacillales;__Staphylococcaceae;Other	0.2	0.23	0.14	1.39	0.23	71.83
Bacteria;__Actinobacteria;__Actinobacteria;Other;Other;Other	0.03	0.19	0.14	1.29	0.23	72.06
Bacteria;__Bacteroidetes;__vadinHA17;__o;__f;__g	0.16	0.34	0.14	1.36	0.23	72.29
Bacteria;__Proteobacteria;__Betaproteobacteria;__Nitrosomonadales;__Nitrosomonadaceae;__Nitrosomonas	0.14	0.21	0.14	1.2	0.23	72.51
Bacteria;__Proteobacteria;__Gammaproteobacteria;__Oceanospirillales;__Oceanospirillaceae;__Pseudospirillum	0	0.18	0.14	1.27	0.23	72.74
Bacteria;__Proteobacteria;__Betaproteobacteria;__Burkholderiales;__Comamonadaceae;__Comamonas	0.15	0.22	0.14	1.17	0.23	72.96
Eukaryota;__Fungi;__Blastocladiomycota;__;__;__	0.19	0	0.13	1.16	0.22	73.19
Eukaryota;__Rhizaria;__Cercozoa;Other;Other;Other	0.19	0.02	0.13	1.43	0.21	73.4
Bacteria;__Firmicutes;__Bacilli;__Lactobacillales;__Aerococcaceae;__Globicatella	0.14	0.13	0.13	1.14	0.21	73.61
Bacteria;__Proteobacteria;__Alphaproteobacteria;__Rhizobiales;__Hyphomicrobiaceae;__Devosia	0	0.17	0.13	1.31	0.21	73.82
Bacteria;__Bacteroidetes;__WCHB1-32;__o;__f;__g	0	0.17	0.12	1.21	0.2	74.02
Bacteria;__Bacteroidetes;Other;Other;Other;Other	0.09	0.19	0.12	1.76	0.2	74.23
Bacteria;__Actinobacteria;__Actinobacteria;__Corynebacteriales;__Mycobacteriaceae;__Mycobacterium	0.05	0.18	0.12	1.2	0.2	74.42
Eukaryota;__Fungi;Other;Other;Other;Other	0.17	0.23	0.12	1.43	0.2	74.62
Bacteria;__Bacteroidetes;__Flavobacteria;__Flavobacteriales;__NS9_marine_group;__g	0.09	0.21	0.12	1.51	0.2	74.82
Bacteria;__Proteobacteria;__Alphaproteobacteria;__Caulobacterales;__Caulobacteraceae;__Phenylobacterium	0	0.16	0.12	1.32	0.19	75.01
Bacteria;__Proteobacteria;__Alphaproteobacteria;__Caulobacterales;__Caulobacteraceae;__Brevundimonas	0.07	0.14	0.11	1.07	0.19	75.2
Bacteria;__Bacteroidetes;__Sphingobacteriia;__Sphingobacteriales;Other;Other	0.02	0.17	0.11	2.51	0.19	75.39
Eukaryota;__Stramenopiles;__Eustigmatales;__Nannochloropsis;__;__	0.16	0	0.11	1.32	0.19	75.58
Bacteria;__Bacteroidetes;__Sphingobacteriia;__Sphingobacteriales;__env.OPS_17;__g	0.13	0.25	0.11	1.51	0.19	75.76
Bacteria;__Candidate_division_OP3;__c;__o;__f;__g	0.11	0.11	0.11	1.41	0.19	75.95
Eukaryota;__Stramenopiles;__CCI40;__;__;__	0.16	0	0.11	1.3	0.19	76.14

Bacteria;__Cyanobacteria;__Cyanobacteria;__SubsectionIII;__FamilyI;__g	0.14	0.1	0.11	1.11	0.19	76.32
Bacteria;__Bacteroidetes;__Flavobacteria;__Flavobacteriales;__Flavobacteriaceae;__Chryseobacterium	0.18	0.08	0.11	1.16	0.19	76.51
Bacteria;__Firmicutes;__Bacilli;__Lactobacillales;__Carnobacteriaceae;__Atopostipes	0.16	0	0.11	1.27	0.18	76.69
Bacteria;__Proteobacteria;Other;Other;Other;Other	0.11	0.24	0.11	1.27	0.18	76.88
Bacteria;__Bacteroidetes;__Bacteroidia;__Bacteroidales;__Rikenellaceae;__Alistipes	0.17	0.09	0.11	1.32	0.18	77.06
Bacteria;__Proteobacteria;__Gammaproteobacteria;__Legionellales;__Legionellaceae;__Legionella	0.1	0.11	0.11	1.81	0.18	77.23
Bacteria;__Proteobacteria;__Deltaproteobacteria;__Desulfuromonadales;Other;Other	0.04	0.16	0.11	1.44	0.18	77.41
Bacteria;__Proteobacteria;__Betaproteobacteria;__Burkholderiales;__Comamonadaceae;__Pelomonas	0.17	0.12	0.11	1.27	0.18	77.59
Bacteria;__Proteobacteria;__Alphaproteobacteria;__Rhodospirillales;__Acetobacteraceae;__g	0	0.15	0.11	1.31	0.18	77.76
Eukaryota;__Alveolata;__Dinoflagellata;__Dinophyceae;__Gymnodiniphycidae;__FV18-2D9	0.16	0.05	0.11	1.45	0.17	77.94
Bacteria;__Bacteroidetes;__Cytophagia;__Cytophagales;__Cytophagaceae;__Emticicia	0.12	0.13	0.1	1.44	0.17	78.1
Bacteria;__Proteobacteria;__Deltaproteobacteria;__Bdellovibrionales;__Bdellovibrionaceae;__Bdellovibrio	0.13	0.09	0.1	4.78	0.17	78.27
Bacteria;__Proteobacteria;__Gammaproteobacteria;__Xanthomonadales;__Sinobacteraceae;__g	0.17	0.04	0.1	2.26	0.17	78.43
Bacteria;__Proteobacteria;__Gammaproteobacteria;__Legionellales;__Coxiellaceae;__Aquicella	0.07	0.14	0.1	1.24	0.16	78.6
Bacteria;__Proteobacteria;__Betaproteobacteria;__Neisseriales;__Neisseriaceae;Other	0.13	0.06	0.1	1.17	0.16	78.76
Bacteria;__Cyanobacteria;__Cyanobacteria;__SubsectionIII;__FamilyI;__Chamaesiphon	0.13	0	0.1	2.08	0.16	78.92
Bacteria;Other;Other;Other;Other;Other	0.13	0.19	0.1	1.28	0.16	79.08
Bacteria;__Firmicutes;__Clostridia;__Clostridiales;__Ruminococcaceae;__Incertae_Sedis	0.13	0.11	0.09	1.57	0.16	79.24
Bacteria;__Chlamydiae;__Chlamydiae;__Chlamydiales;__Parachlamydiaceae;__Neochlamydia	0.1	0.04	0.09	0.88	0.16	79.39
Bacteria;__Actinobacteria;__Actinobacteria;__Propionibacteriales;__Propionibacteriaceae;Other	0	0.12	0.09	0.66	0.15	79.55
Eukaryota;__Stramenopiles;__Chrysophyceae;__Ochromonadales;__Paraphysomonas;__	0.14	0.03	0.09	1.55	0.15	79.7
Bacteria;__Proteobacteria;__Gammaproteobacteria;Other;Other;Other	0.09	0.2	0.09	1.18	0.15	79.85
Bacteria;__Proteobacteria;__Gammaproteobacteria;__Pseudomonadales;__Moraxellaceae;__Alkanindiges	0.13	0	0.09	1.31	0.15	80
Eukaryota;__RT5iin25;__;__;__;__	0.04	0.11	0.09	0.83	0.15	80.14

Groups Atlas Pool & Atlas Cove

Average dissimilarity = 92.23

Species	Atlas Pool Av.Abund	Atlas Cove Av.Abund	Av.Diss	Diss/SD	Contrib%	Cum.%
Eukaryota;__Alveolata;__Ciliophora;__Spirotrichea;__Hypotrichia;Other	3.49	0	2.73	2.71	2.97	2.97
Bacteria;__Proteobacteria;__Gammaproteobacteria;__Oceanospirillales;__ZD0405;__g	0	3.19	2.55	1.93	2.77	5.73
Bacteria;__Proteobacteria;__Gammaproteobacteria;Other;Other;Other	0.09	3.15	2.35	2.63	2.55	8.29

Bacteria;__Proteobacteria;__Alphaproteobacteria;__SAR11_clade;__Surface_1;__g	0	2.82	2.28	1.36	2.47	10.76
Bacteria;__Proteobacteria;__Betaproteobacteria;__Burkholderiales;__Comamonadaceae;Other	2.66	0.03	1.92	1.87	2.09	12.85
Bacteria;__Actinobacteria;__Actinobacteria;__Frankiales;__Sporichthyaceae;__hgcI_clade	2.32	0	1.91	0.92	2.07	14.92
Bacteria;__Cyanobacteria;__Chloroplast;__Chloroplast;__Chloroplast;__Chloroplast	3.27	2.38	1.7	1.23	1.85	16.77
Eukaryota;__Chloroplastida;__Chlorophyceae;__Chlamydomonas;__;__	2.25	0	1.64	1.65	1.78	18.55
Bacteria;__Bacteroidetes;__Flavobacteria;__Flavobacteriales;__Flavobacteriaceae;__Flavobacterium	2.2	0.03	1.61	2.39	1.75	20.3
Bacteria;__Actinobacteria;__Actinobacteria;__Micrococcales;__Microbacteriaceae;Other	1.99	0	1.6	1.16	1.74	22.04
Bacteria;__Proteobacteria;__Gammaproteobacteria;__Alteromonadales;__Alteromonadaceae;__SAR92_clade	0	2.09	1.6	2.36	1.73	23.77
Bacteria;__Proteobacteria;__Gammaproteobacteria;__Oceanospirillales;__SAR86_clade;__g	0	1.99	1.57	2.34	1.71	25.47
Bacteria;__Bacteroidetes;__Flavobacteria;__Flavobacteriales;__Flavobacteriaceae;__NS2b_marine_group	0	1.92	1.49	3.75	1.62	27.09
Bacteria;__Proteobacteria;__Betaproteobacteria;__Burkholderiales;__Burkholderiaceae;__Polynucleobacter	1.75	0	1.42	1.06	1.54	28.63
Eukaryota;__Chloroplastida;__Chlorophyceae;Other;Other;Other	1.85	0	1.34	1.47	1.45	30.09
Bacteria;__Bacteroidetes;__Flavobacteria;__Flavobacteriales;__Flavobacteriaceae;__Polaribacter	0	1.7	1.3	4.16	1.41	31.5
Bacteria;__Proteobacteria;__Alphaproteobacteria;__Rhodobacterales;__Rhodobacteraceae;__Roseobacter_clade_DC5-80-3_lineage	0	1.59	1.25	2.76	1.35	32.85
Bacteria;__Bacteroidetes;__Flavobacteria;__Flavobacteriales;__NS9_marine_group;__g	0.09	1.54	1.1	6.69	1.2	34.05
Bacteria;__Bacteroidetes;__Flavobacteria;__Flavobacteriales;__Flavobacteriaceae;__NS4_marine_group	0	1.31	1.05	1.84	1.14	35.19
Archaea;__Euryarchaeota;__Thermoplasmata;__Thermoplasmatales;__Marine_Group_II;__g	0	1.28	1.02	1.92	1.11	36.3
Bacteria;__Proteobacteria;__Betaproteobacteria;__Burkholderiales;__Comamonadaceae;__Polaromonas	1.23	0	0.91	1.99	0.99	37.29
Bacteria;__Proteobacteria;__Betaproteobacteria;__Burkholderiales;__Alcaligenaceae;__Achromobacter	1.09	0	0.84	2.17	0.91	38.2
Bacteria;__Actinobacteria;__Actinobacteria;__Frankiales;__Sporichthyaceae;Other	1.02	0	0.84	0.94	0.91	39.11
Eukaryota;__Rhizaria;__Cercozoa;__Endomyxa;__Novel Clade 10;__	1.09	0	0.81	2.07	0.88	39.99
Bacteria;__Proteobacteria;__Gammaproteobacteria;__Alteromonadales;__Pseudoalteromonadaceae;__Pseudoalteromonas	0.08	1.18	0.8	1.48	0.87	40.86
Bacteria;__Proteobacteria;__Gammaproteobacteria;__Alteromonadales;__Alteromonadaceae;__Alteromonas	0	1.1	0.78	1.32	0.85	41.71
Bacteria;__Bacteroidetes;__Flavobacteria;__Flavobacteriales;__Flavobacteriaceae;Other	0.03	1.01	0.78	1.64	0.85	42.56
Bacteria;__Proteobacteria;__Alphaproteobacteria;__SAR11_clade;Other;Other	0	0.9	0.72	1.66	0.78	43.34
Eukaryota;__Stramenopiles;__MAST-1;__MAST-1 B;__;__	0	1	0.72	1.29	0.78	44.11
Bacteria;__Proteobacteria;__Betaproteobacteria;__Burkholderiales;__Comamonadaceae;__Simplicispira	0.91	0	0.66	1.95	0.72	44.83
Bacteria;__Proteobacteria;__Alphaproteobacteria;__Rhodobacterales;__Rhodobacteraceae;__g	0.03	0.85	0.63	2.06	0.68	45.51
Eukaryota;__Fungi;__Chytridiomycota;__;__;__	0.86	0	0.62	1.76	0.67	46.19
Bacteria;__Bacteroidetes;__Flavobacteria;__Flavobacteriales;Other;Other	0	0.84	0.62	2.34	0.67	46.85
Eukaryota;Other;Other;Other;Other;Other	0.09	0.97	0.61	0.81	0.67	47.52

Eukaryota;__Haptophyta;__Prymnesiophyceae;Other;Other;Other	0	0.86	0.61	1.32	0.66	48.18
Bacteria;__Proteobacteria;__Alphaproteobacteria;__Rhodobacterales;__Rhodobacteraceae;Other	1.03	0.29	0.57	1.59	0.62	48.8
Bacteria;__Actinobacteria;__Actinobacteria;__PeM15;__f;__g	0.79	0.08	0.57	1.11	0.62	49.41
Eukaryota;__Alveolata;__Dinoflagellata;__Dinophyceae;__Gymnodiniphycidae;__Gyrodinium	0	0.77	0.52	0.75	0.57	49.98
Archaea;__Thaumarchaeota;__Marine_Group_I;__o;__f;__g	0	0.65	0.52	1.5	0.56	50.55
Bacteria;__Proteobacteria;__Alphaproteobacteria;__Rhodobacterales;__Rhodobacteraceae;__Sulfitobacter	0	0.73	0.52	1.3	0.56	51.11
Bacteria;__Proteobacteria;__Gammaproteobacteria;__Oceanospirillales;__Oceanospirillaceae;Other	0	0.68	0.52	4.49	0.56	51.67
Eukaryota;__Metazoa;__Arthropoda;__Maxillopoda;__;__	0.24	0.78	0.49	0.88	0.54	52.21
Bacteria;__Bacteroidetes;__Flavobacteria;__Flavobacteriales;__Flavobacteriaceae;__NS5_marine_group	0	0.6	0.49	1.26	0.53	52.74
Bacteria;__Proteobacteria;__Gammaproteobacteria;__Oceanospirillales;__Alcanivoracaceae;__Alcanivorax	0	0.67	0.49	2.39	0.53	53.27
Eukaryota;__Picozoa;__;__;__;__	0	0.68	0.48	1.28	0.52	53.78
Eukaryota;__Alveolata;__Ciliophora;__Spirotrichea;__Oligotrichia;__uncultured	0	0.66	0.47	1.17	0.51	54.29
Bacteria;__Deferribacteres;__Deferribacteres;__Deferribacterales;__SAR406_clade(Marine_group_A);__g	0	0.55	0.46	1.17	0.5	54.79
Bacteria;__Candidate_division_OP11;__c;__o;__f;__g	0.55	0	0.45	0.86	0.49	55.28
Bacteria;__Proteobacteria;__Alphaproteobacteria;__Rickettsiales;__SAR116_clade;__g	0	0.57	0.45	2.57	0.49	55.77
Bacteria;__Proteobacteria;__Betaproteobacteria;__Burkholderiales;__Comamonadaceae;__Paucibacter	0.61	0	0.45	1.78	0.49	56.26
Eukaryota;__Stramenopiles;__MAST-7;__;__;__	0	0.62	0.45	1.25	0.49	56.75
Bacteria;__Proteobacteria;__Betaproteobacteria;__Burkholderiales;__Alcaligenaceae;Other	0.57	0	0.44	2.18	0.47	57.22
Eukaryota;__Stramenopiles;__MAST-3;__;__;__	0	0.61	0.44	1.29	0.47	57.7
Eukaryota;__Rhizaria;__Cercozoa;__Endomyxa;__Phytomyxea;__Spongospora	0.59	0	0.43	2.42	0.47	58.16
Bacteria;__Actinobacteria;__Actinobacteria;__Micrococcales;__Microbacteriaceae;__Candidatus_Aquiluna	0.53	0	0.42	1.41	0.46	58.62
Bacteria;__Proteobacteria;__Gammaproteobacteria;__Oceanospirillales;__OM182_clade;__g	0	0.55	0.42	2.23	0.45	59.08
Bacteria;__Proteobacteria;__Gammaproteobacteria;__Alteromonadales;__Colwelliaceae;__Colwellia	0.05	0.61	0.4	1.33	0.44	59.51
Eukaryota;__Stramenopiles;__Diatomea;__Bacillariophytina;__Mediophyceae;Other	0.58	0.11	0.4	1.41	0.43	59.94
Bacteria;__Proteobacteria;__Alphaproteobacteria;__SAR11_clade;__Surface_4;__g	0	0.49	0.39	1.64	0.43	60.37
Bacteria;__Actinobacteria;__Actinobacteria;__Micrococcales;__Microbacteriaceae;__Candidatus_Limnoluna	0.45	0	0.36	1.19	0.39	60.76
Bacteria;__Proteobacteria;__Gammaproteobacteria;__Pseudomonadales;__Moraxellaceae;__Psychrobacter	0.5	0	0.36	1.56	0.39	61.15
Eukaryota;__Alveolata;__Protalveolata;__Syndiniales;__Syndiniales Group I;__	0	0.48	0.34	1.3	0.37	61.52
Bacteria;__Proteobacteria;__Gammaproteobacteria;__Alteromonadales;__Alteromonadaceae;Other	0	0.45	0.33	3.42	0.36	61.89
Bacteria;__Proteobacteria;__Betaproteobacteria;__Burkholderiales;__Comamonadaceae;__g	0.45	0	0.33	1.2	0.36	62.25
Bacteria;__Proteobacteria;__Alphaproteobacteria;Other;Other;Other	0	0.42	0.33	1.76	0.36	62.61
Bacteria;__Bacteroidetes;__Flavobacteria;__Flavobacteriales;__Cryomorphaceae;__Owenweeksia	0.07	0.47	0.32	1.87	0.35	62.96

Eukaryota;__Alveolata;__Ciliophora;__Spirotrichea;__Oligotrichia;__Strombidium	0	0.47	0.32	0.83	0.35	63.31
Unassigned;Other;Other;Other;Other;Other	1.1	1.01	0.32	1.41	0.35	63.65
Bacteria;__Proteobacteria;__Deltaproteobacteria;__Myxococcales;__0319-6G20;__g	0.41	0	0.32	2.46	0.34	64
Bacteria;__Proteobacteria;__Betaproteobacteria;__Burkholderiales;Other;Other	0.38	0	0.31	0.98	0.34	64.33
Eukaryota;__Holozoa;__Choanomonada;__Acanthoecida;__Diaphanoeca;__	0	0.45	0.31	0.93	0.34	64.67
Eukaryota;__Chloroplastida;__Trebouxiophyceae;__;__;__	0.4	0	0.3	1.99	0.32	65
Bacteria;__Bacteroidetes;__Flavobacteria;__Flavobacteriales;__Flavobacteriaceae;__NS3a_marine_group	0	0.38	0.29	6.05	0.32	65.31
Eukaryota;__Alveolata;__Protalveolata;__Syndiniales;__Syndiniales Group II;__	0	0.41	0.29	1.27	0.32	65.63
Bacteria;__Bacteroidetes;__Sphingobacteriia;__Sphingobacteriales;__NS11-12_marine_group;__g	0.41	0	0.29	1.47	0.32	65.95
Bacteria;__Proteobacteria;__Deltaproteobacteria;__SAR324_clade(Marine_group_B);__f;__g	0	0.35	0.29	1.3	0.31	66.26
Bacteria;__Proteobacteria;__Gammaproteobacteria;__Xanthomonadales;__Xanthomonadaceae;__Arenimonas	0.38	0	0.29	2.09	0.31	66.57
Eukaryota;__Stramenopiles;__MAST-1;__MAST-1 A;__;__	0	0.4	0.28	1.31	0.3	66.88
Eukaryota;__Stramenopiles;__T53A1;__;__;__	0.37	0	0.28	1.96	0.3	67.18
Bacteria;__Bacteroidetes;__Cytophagia;__Cytophagales;__Flammeovirgaceae;__Marinoscillum	0	0.33	0.27	0.89	0.3	67.47
Bacteria;__Bacteroidetes;__Flavobacteria;__Flavobacteriales;__Flavobacteriaceae;__g	0.03	0.39	0.27	3.59	0.29	67.77
Eukaryota;__Alveolata;__Dinoflagellata;__Dinophyceae;__Gymnodiniphycidae;__Gymnodinium	0	0.39	0.27	0.94	0.29	68.06
Eukaryota;__Stramenopiles;__Bicosoecida;__Siluaniidae;__Siluania;__	0.36	0	0.27	1.77	0.29	68.35
Bacteria;__Proteobacteria;__Betaproteobacteria;__Burkholderiales;__Oxalobacteraceae;__Undibacterium	0.36	0	0.26	1.73	0.29	68.64
Bacteria;__Proteobacteria;__Alphaproteobacteria;__SAR11_clade;__f;__g	0	0.32	0.26	1.15	0.28	68.92
Bacteria;__Fusobacteria;__Fusobacteria;__Fusobacteriales;__Fusobacteriaceae;__Fusobacterium	0.36	0	0.26	1.24	0.28	69.2
Bacteria;__Firmicutes;__Clostridia;__Clostridiales;__Ruminococcaceae;__g	0.35	0	0.25	1.47	0.28	69.47
Eukaryota;__Alveolata;__Ciliophora;__Spirotrichea;__Choreotrichia;__uncultured	0	0.37	0.25	0.85	0.28	69.75
Eukaryota;__Alveolata;__Dinoflagellata;__Dinophyceae;__Gymnodiniphycidae;Other	0.03	0.38	0.25	0.77	0.27	70.02
Eukaryota;__Alveolata;__Ciliophora;__Spirotrichea;__Hypotrichia;__Gonostomum	0.29	0	0.25	0.66	0.27	70.29
Bacteria;__Proteobacteria;__Alphaproteobacteria;__Rickettsiales;__mitochondria;__g	0.37	0.05	0.25	1.61	0.27	70.56
Bacteria;__Proteobacteria;__Gammaproteobacteria;__Pseudomonadales;__Moraxellaceae;__Perlucidibaca	0.27	0.49	0.25	1.11	0.27	70.82
Eukaryota;__Chloroplastida;__Mamiellophyceae;__Micromonas;__;__	0	0.33	0.24	1.14	0.26	71.09
Bacteria;__Proteobacteria;__Gammaproteobacteria;__Oceanospirillales;Other;Other	0	0.3	0.24	2.22	0.26	71.35
Bacteria;__Proteobacteria;__Alphaproteobacteria;__Rhodobacterales;__Rhodobacteraceae;__Rhodobacter	0.33	0	0.24	1.3	0.26	71.61
Eukaryota;__Alveolata;__Protalveolata;__Colpodellida;Other;Other	0.33	0	0.24	1.24	0.26	71.87
Archaea;__Euryarchaeota;__Halobacteria;__Halobacteriales;__Deep_Sea_Hydrothermal_Vent_Gp_6(DHVEG-6);__g	0.29	0	0.24	0.9	0.26	72.13
Eukaryota;__Rhizaria;__Cercozoa;__Thecofilosea;__Cryomonadida;__Cryothecomonas	0	0.34	0.23	0.88	0.25	72.38

Bacteria;__Proteobacteria;__Betaproteobacteria;__Burkholderiales;__Comamonadaceae;__Albidiferax	0.3	0	0.23	6.38	0.25	72.63
Bacteria;__Actinobacteria;__Actinobacteria;__Micrococcales;__Microbacteriaceae;__Candidatus_Planktoluna	0.28	0	0.23	1.06	0.24	72.87
Bacteria;__Proteobacteria;__Betaproteobacteria;Other;Other;Other	0.64	0.45	0.23	1.14	0.24	73.12
Eukaryota;__Metazoa;__Cnidaria;__Hydrozoa;__;__	0	0.33	0.22	0.66	0.24	73.36
Eukaryota;__Stramenopiles;__MAST-8;__;__;__	0	0.29	0.21	1.22	0.23	73.59
Bacteria;__Bacteroidetes;__Cytophagia;__Cytophagales;__Flammeovirgaceae;Other	0	0.27	0.21	1.89	0.23	73.81
Bacteria;__Bacteroidetes;__Sphingobacteriia;__Sphingobacteriales;__Chitinophagaceae;__g	0.28	0	0.2	1.17	0.22	74.04
Bacteria;__Bacteroidetes;__Bacteroidia;__Bacteroidales;__Bacteroidaceae;__Bacteroides	0.31	0.04	0.2	1.01	0.22	74.25
Eukaryota;__Stramenopiles;__Chrysophyceae;__Chromulinales;__Phaeoplaca;__	0.26	0	0.2	6.07	0.21	74.47
Bacteria;__Proteobacteria;__Gammaproteobacteria;__Oceanospirillales;__oc58;__g	0.27	0	0.2	1.31	0.21	74.68
Bacteria;__Proteobacteria;__Alphaproteobacteria;__Sphingomonadales;__Sphingomonadaceae;__Sphingopyxis	0	0.26	0.2	3.59	0.21	74.89
Eukaryota;__Haptophyta;__Prymnesiophyceae;__Prymnesiales;Other;Other	0	0.27	0.2	1.32	0.21	75.11
Bacteria;__Proteobacteria;__Gammaproteobacteria;__Oceanospirillales;__Oceanospirillaceae;__Balneatrix	0	0.25	0.19	1.6	0.21	75.32
Bacteria;__Proteobacteria;__Alphaproteobacteria;__Rhodospirillales;__Rhodospirillaceae;__Defluviicoccus	0	0.25	0.19	2.64	0.21	75.53
Bacteria;__Proteobacteria;__Deltaproteobacteria;__Bdellovibrionales;__Bacteriovoracaceae;__Bacteriovorax	0.24	0	0.19	1.58	0.21	75.73
Bacteria;__Proteobacteria;__Betaproteobacteria;__Burkholderiales;__Comamonadaceae;__Hydrogenophaga	0.26	0	0.19	1.1	0.21	75.94
Bacteria;__Bacteroidetes;__Sphingobacteriia;__Sphingobacteriales;__Chitinophagaceae;__Filimonas	0.26	0	0.19	1.2	0.21	76.14
Eukaryota;__Chloroplastida;__Chlorophyceae;__Desmodesmus;__;__	0.27	0.03	0.19	1.44	0.2	76.35
Eukaryota;__Alveolata;__Ciliophora;__Conthreep;__Oligohymenophorea;Other	0.27	0.06	0.19	1.16	0.2	76.55
Bacteria;__Candidate_division_TM7;__c;__o;__f;__g	0.23	0	0.18	1.69	0.2	76.75
Eukaryota;__Metazoa;__Annelida;__;__;__	0	0.27	0.18	1	0.2	76.95
Bacteria;__Proteobacteria;__Betaproteobacteria;__Burkholderiales;__Burkholderiaceae;__Ralstonia	0.23	0	0.18	1.09	0.2	77.15
Bacteria;__Verrucomicrobia;__Opitutae;__MB11C04_marine_group;__f;__g	0	0.24	0.18	2.37	0.2	77.34
Bacteria;__Bacteroidetes;__Sphingobacteriia;__Sphingobacteriales;__WCHB1-69;__g	0.24	0	0.17	1.18	0.19	77.53
Bacteria;__Proteobacteria;__Betaproteobacteria;__Rhodocyclales;__Rhodocyclaceae;__Dechloromonas	0.23	0	0.17	2.81	0.19	77.72
Bacteria;__Proteobacteria;__Betaproteobacteria;__Burkholderiales;__Alcaligenaceae;__Pusillimonas	0.2	0	0.17	0.66	0.19	77.9
Eukaryota;__Stramenopiles;__MAST-1;__MAST-1 C;__;__	0	0.24	0.16	0.92	0.18	78.08
Bacteria;__Actinobacteria;__Actinobacteria;__Micrococcales;__Microbacteriaceae;__Alpinimonas	0.19	0	0.16	0.66	0.18	78.26
Eukaryota;__Chloroplastida;__Chlorophyceae;__;__;__	0.22	0	0.16	1.16	0.17	78.43

Archaea;__Euryarchaeota;__Halobacteria;__Halobacteriales;__Deep_Sea_Hydrothermal_Vent_Gp_6(DHVEG-6);__Candidatus_Parvarchaeum	0.19	0	0.16	0.87	0.17	78.6
Bacteria;__Candidate_division_OD1;__c;__o;__f;__g	0.2	0	0.16	1.66	0.17	78.77
Eukaryota;__Alveolata;__Ciliophora;__Conthreep;__Prostomatea;__Cryptocaryon	0.26	0.09	0.16	1.69	0.17	78.94
Eukaryota;__Alveolata;__Ciliophora;__Spirotrichea;__Choreotrichia;Other	0	0.22	0.15	0.94	0.17	79.11
Bacteria;__Proteobacteria;__Alphaproteobacteria;__Rhodospirillales;__Rhodospirillaceae;__g	0	0.2	0.15	6.14	0.17	79.27
Bacteria;__WCHB1-60;__c;__o;__f;__g	0.18	0	0.15	0.76	0.17	79.44
Eukaryota;__Telonema;__Incertae Sedis;__;__;__	0	0.23	0.15	0.66	0.17	79.61
Bacteria;__Proteobacteria;__Gammaproteobacteria;__Xanthomonadales;__Xanthomonadaceae;Other	0.21	0	0.15	1.13	0.17	79.77
Eukaryota;__Chloroplastida;__Prasinophytae;__Pyramimonas;__;__	0	0.22	0.15	0.85	0.17	79.94
Bacteria;__Proteobacteria;__Gammaproteobacteria;__Order_Incertae_Sedis;__Family_Incertae_Sedis;__Marinicella	0	0.18	0.15	0.86	0.16	80.1

Groups Seal Pool & Atlas Cove

Average dissimilarity = 92.34

Species	Seal Pool Av.Abund	Atlas Cove Av.Abund	Av.Diss	Diss/SD	Contrib%	Cum.%
Bacteria;__Actinobacteria;__Actinobacteria;__PeM15;__f;__g	3.86	0.08	2.79	1.61	3.02	3.02
Bacteria;__Proteobacteria;__Gammaproteobacteria;__Oceanospirillales;__ZD0405;__g	0.08	3.19	2.37	1.94	2.57	5.59
Bacteria;__Bacteroidetes;__Flavobacteria;__Flavobacteriales;__Flavobacteriaceae;__Flavobacterium	3.15	0.03	2.25	1.9	2.44	8.03
Bacteria;__Proteobacteria;__Gammaproteobacteria;Other;Other;Other	0.2	3.15	2.15	2.67	2.33	10.36
Bacteria;__Proteobacteria;__Alphaproteobacteria;__SAR11_clade;__Surface_1;__g	0.06	2.82	2.12	1.36	2.3	12.65
Bacteria;__Proteobacteria;__Betaproteobacteria;__Burkholderiales;__Comamonadaceae;Other	2.78	0.03	1.99	2.4	2.15	14.8
Bacteria;__Proteobacteria;__Betaproteobacteria;__Burkholderiales;__Burkholderiaceae;__Polynucleobacter	2.03	0	1.52	1.17	1.64	16.45
Bacteria;__Proteobacteria;__Gammaproteobacteria;__Alteromonadales;__Alteromonadaceae;__SAR92_clade	0.07	2.09	1.47	2.33	1.6	18.04
Bacteria;__Proteobacteria;__Gammaproteobacteria;__Oceanospirillales;__SAR86_clade;__g	0.06	1.99	1.45	2.36	1.57	19.61
Bacteria;__Proteobacteria;__Betaproteobacteria;__Burkholderiales;__Comamonadaceae;__Polaromonas	2	0	1.45	2.22	1.56	21.18
Bacteria;__Bacteroidetes;__Flavobacteria;__Flavobacteriales;__Flavobacteriaceae;__NS2b_marine_group	0.09	1.92	1.35	3.9	1.46	22.64
Bacteria;__Cyanobacteria;__Chloroplast;__Chloroplast;__Chloroplast;__Chloroplast	0.84	2.38	1.29	1.48	1.4	24.04
Bacteria;__Bacteroidetes;__Flavobacteria;__Flavobacteriales;__Flavobacteriaceae;__Polaribacter	0.06	1.7	1.19	4.15	1.29	25.33
Bacteria;__Proteobacteria;__Alphaproteobacteria;__Rhodobacterales;__Rhodobacteraceae;Other	1.79	0.29	1.16	1.29	1.26	26.59

Bacteria;__Proteobacteria;__Alphaproteobacteria;__Rhodobacterales;__Rhodobacteraceae;__Roseobacter_clade_DC5-80-3_lineage	0.07	1.59	1.14	2.76	1.23	27.82
Bacteria;__Bacteroidetes;__Flavobacteria;__Flavobacteriales;__Flavobacteriaceae;__NS4_marine_group	0.05	1.31	0.97	1.83	1.05	28.87
Bacteria;__Bacteroidetes;__Flavobacteria;__Flavobacteriales;__NS9_marine_group;__g	0.21	1.54	0.96	7.17	1.04	29.92
Archaea;__Euryarchaeota;__Thermoplasmata;__Thermoplasmatales;__Marine_Group_II;__g	0.05	1.28	0.94	1.91	1.02	30.94
Bacteria;__Actinobacteria;__Actinobacteria;__Frankiales;__Sporichthyaceae;__hgcI_clade	1.22	0	0.91	1.07	0.99	31.92
Bacteria;__Proteobacteria;__Betaproteobacteria;__Burkholderiales;__Comamonadaceae;__Simplicispira	1.25	0	0.9	1.65	0.97	32.89
Bacteria;__Actinobacteria;__Actinobacteria;__Micrococcales;__Microbacteriaceae;Other	1.18	0	0.88	1.34	0.95	33.85
Bacteria;__Bacteroidetes;__Sphingobacteriia;__Sphingobacteriales;__Chitinophagaceae;__Sediminibacterium	1.15	0	0.83	2.48	0.9	34.75
Bacteria;__Proteobacteria;__Betaproteobacteria;__Burkholderiales;__Comamonadaceae;__g	1.15	0	0.83	1.37	0.9	35.65
Bacteria;__Bacteroidetes;__Sphingobacteriia;__Sphingobacteriales;__NS11-12_marine_group;__g	1.14	0	0.82	1.42	0.88	36.53
Bacteria;__Bacteroidetes;__Sphingobacteriia;__Sphingobacteriales;__Sphingobacteriaceae;__Pedobacter	1.06	0	0.78	1.93	0.84	37.37
Bacteria;__Proteobacteria;__Gammaproteobacteria;__Alteromonadales;__Pseudoalteromonadaceae;__Pseudoalteromonas	0.06	1.18	0.77	1.49	0.83	38.2
Bacteria;__Actinobacteria;__Actinobacteria;__Frankiales;__Sporichthyaceae;Other	1.02	0	0.76	1.05	0.82	39.03
Bacteria;__Proteobacteria;__Gammaproteobacteria;__Alteromonadales;__Alteromonadaceae;__Alteromonas	0	1.1	0.75	1.33	0.81	39.84
Bacteria;__Bacteroidetes;__Flavobacteria;__Flavobacteriales;__Flavobacteriaceae;Other	0.05	1.01	0.73	1.64	0.79	40.63
Eukaryota;__Chloroplastida;__Chlorophyceae;__Desmodesmus;__;__	1.02	0.03	0.72	1.08	0.78	41.41
Bacteria;__Proteobacteria;__Betaproteobacteria;__Burkholderiales;__Comamonadaceae;__Paucibacter	0.97	0	0.7	1.22	0.75	42.16
Eukaryota;__Stramenopiles;__MAST-1;__MAST-1 B;__;__	0	1	0.69	1.31	0.74	42.9
Eukaryota;__Rhizaria;__Cercozoa;__Thecofilosea;Other;Other	1.1	0.19	0.68	1.49	0.74	43.64
Bacteria;__Proteobacteria;__Alphaproteobacteria;__SAR11_clade;Other;Other	0.05	0.9	0.65	1.62	0.71	44.35
Eukaryota;Other;Other;Other;Other;Other	0	0.97	0.64	0.89	0.69	45.04
Bacteria;__Proteobacteria;__Alphaproteobacteria;__Rhodobacterales;__Rhodobacteraceae;__g	0	0.85	0.62	2.21	0.67	45.71
Bacteria;__Bacteroidetes;__Sphingobacteriia;__Sphingobacteriales;__Chitinophagaceae;__Ferruginibacter	0.86	0	0.62	1.51	0.67	46.38
Bacteria;__Bacteroidetes;__Flavobacteria;__Flavobacteriales;Other;Other	0	0.84	0.59	2.36	0.64	47.02
Eukaryota;__Haptophyta;__Prymnesiophyceae;Other;Other;Other	0	0.86	0.58	1.33	0.63	47.65
Bacteria;__Actinobacteria;Other;Other;Other;Other	0.78	0	0.58	1.66	0.63	48.28
Bacteria;__Proteobacteria;__Betaproteobacteria;__Burkholderiales;__Comamonadaceae;__Albidiferax	0.77	0	0.55	1.46	0.6	48.87
Bacteria;__Bacteroidetes;__Sphingobacteriia;__Sphingobacteriales;__Saprospiraceae;__g	0.76	0	0.55	0.95	0.59	49.46
Bacteria;__Candidate_division_OP11;__c;__o;__f;__g	0.72	0	0.54	1.19	0.59	50.05
Bacteria;__Bacteroidetes;__Sphingobacteriia;__Sphingobacteriales;__Chitinophagaceae;__g	0.71	0	0.52	1.25	0.56	50.61
Bacteria;__Proteobacteria;__Alphaproteobacteria;__Rhodospirillales;__Acetobacteraceae;__Roseococcus	0.71	0	0.51	1.37	0.55	51.16
Eukaryota;__Metazoa;__Arthropoda;__Maxillopoda;__;__	0	0.78	0.51	0.81	0.55	51.71

Bacteria;__Bacteroidetes;__Flavobacteria;__Flavobacteriales;__Cryomorphaceae;__Fluviicola	0.9	0.2	0.51	2.21	0.55	52.26
Eukaryota;__Alveolata;__Dinoflagellata;__Dinophyceae;__Gymnodiniphycidae;__Gyrodinium	0	0.77	0.5	0.75	0.54	52.81
Bacteria;__Proteobacteria;__Alphaproteobacteria;__Rhodobacterales;__Rhodobacteraceae;__Sulfitobacter	0	0.73	0.5	1.32	0.54	53.34
Bacteria;__Proteobacteria;__Betaproteobacteria;__Burkholderiales;__Alcaligenaceae;__Achromobacter	0.67	0	0.5	2.41	0.54	53.88
Archaea;__Thaumarchaeota;__Marine_Group_I;__o;__f;__g	0	0.65	0.49	1.54	0.53	54.42
Bacteria;__Proteobacteria;__Alphaproteobacteria;__Sphingomonadales;__Sphingomonadaceae;__Novosphingobium	0.68	0	0.49	2.07	0.53	54.95
Bacteria;__Proteobacteria;__Betaproteobacteria;__Burkholderiales;__Alcaligenaceae;Other	0.64	0	0.47	2.6	0.5	55.46
Bacteria;__Bacteroidetes;__Flavobacteria;__Flavobacteriales;__Flavobacteriaceae;__NS5_marine_group	0	0.6	0.46	1.28	0.5	55.96
Bacteria;__Proteobacteria;__Gammaproteobacteria;__Oceanospirillales;__Oceanospirillaceae;Other	0.05	0.68	0.46	3.98	0.5	56.46
Eukaryota;__Picozoa;__;__;__;__	0	0.68	0.46	1.29	0.49	56.95
Bacteria;__Actinobacteria;__Actinobacteria;__Micrococcales;__Microbacteriaceae;__Candidatus_Aquiluna	0.62	0	0.46	1.61	0.49	57.45
Eukaryota;__Fungi;__Chytridiomycota;__;__;__	0.63	0	0.45	1.19	0.49	57.93
Eukaryota;__Alveolata;__Ciliophora;__Spirotrichea;__Oligotrichia;__uncultured	0	0.66	0.45	1.17	0.49	58.42
Archaea;__Euryarchaeota;__Methanomicrobia;__Methanomicrobiales;__Methanoregulaceae;__Methanoregula	0.61	0	0.45	8.52	0.48	58.91
Bacteria;__Proteobacteria;__Gammaproteobacteria;__Oceanospirillales;__Alcanivoracaceae;__Alcanivorax	0.02	0.67	0.45	2.29	0.48	59.39
Bacteria;__Deferribacteres;__Deferribacteres;__Deferribacterales;__SAR406_clade(Marine_group_A);__g	0	0.55	0.43	1.19	0.47	59.86
Bacteria;__Proteobacteria;__Alphaproteobacteria;__Rickettsiales;__SAR116_clade;__g	0	0.57	0.43	2.7	0.46	60.32
Eukaryota;__Stramenopiles;__MAST-7;__;__;__	0	0.62	0.43	1.26	0.46	60.79
Eukaryota;__Stramenopiles;__MAST-3;__;__;__	0	0.61	0.42	1.31	0.45	61.24
Bacteria;__Proteobacteria;__Gammaproteobacteria;__Alteromonadales;__Colwelliaceae;__Colwellia	0	0.61	0.42	1.5	0.45	61.69
Bacteria;__Proteobacteria;__Gammaproteobacteria;__Pseudomonadales;__Moraxellaceae;__Psychrobacter	0.53	0	0.38	1.92	0.42	62.1
Bacteria;__Proteobacteria;__Betaproteobacteria;__Burkholderiales;Other;Other	0.53	0	0.38	2.58	0.41	62.52
Bacteria;__Proteobacteria;__Alphaproteobacteria;__SAR11_clade;__Surface_4;__g	0	0.49	0.37	1.68	0.4	62.92
Bacteria;__Proteobacteria;__Deltaproteobacteria;__Bdellovibrionales;__Bacteriovoracaceae;__Bacteriovorax	0.5	0	0.37	4.64	0.4	63.32
Bacteria;__Actinobacteria;__Acidimicrobiia;__Acidimicrobiales;__Acidimicrobiaceae;__CL500-29_marine_group	0.49	0	0.37	0.97	0.4	63.72
Bacteria;__Proteobacteria;__Gammaproteobacteria;__Oceanospirillales;__OM182_clade;__g	0.05	0.55	0.37	1.99	0.4	64.11
Bacteria;__Bacteroidetes;__Cytophagia;__Cytophagales;__Cytophagaceae;__Leadbetterella	0.5	0	0.36	1.12	0.39	64.5
Bacteria;__Bacteroidetes;__Flavobacteria;__Flavobacteriales;__Cryomorphaceae;__Owenweeksia	0	0.47	0.36	2.34	0.39	64.89

Bacteria;__Proteobacteria;__Gammaproteobacteria;__Pseudomonadales;__Moraxellaceae;__Perlucidibaca	0.76	0.49	0.35	1.56	0.38	65.27
Eukaryota;__Alveolata;__Protalveolata;__Syndiniales;__Syndiniales Group I;__	0	0.48	0.33	1.31	0.35	65.62
Bacteria;__Bacteroidetes;__Sphingobacteriia;__Sphingobacteriales;__WCHB1-69;__g	0.44	0	0.32	1.13	0.34	65.96
Bacteria;__Candidate_division_TM7;__c;__o;__f;__g	0.41	0	0.31	0.84	0.34	66.3
Archaea;__Euryarchaeota;__Halobacteria;__Halobacteriales;__Deep_Sea_Hydrothermal_Vent_Gp_6(DHVEG-6);__g	0.41	0	0.31	0.93	0.34	66.63
Eukaryota;__Alveolata;__Ciliophora;__Spirotrichea;__Oligotrichia;__Strombidium	0	0.47	0.31	0.84	0.33	66.97
Bacteria;__Bacteroidetes;__Sphingobacteriia;__Sphingobacteriales;__Chitinophagaceae;__Filimonas	0.42	0	0.3	0.94	0.33	67.29
Bacteria;__Actinobacteria;__Acidimicrobiia;__Acidimicrobiales;__uncultured;__g	0.41	0	0.3	2.77	0.33	67.62
Bacteria;__Proteobacteria;__Gammaproteobacteria;__Alteromonadales;__Alteromonadaceae;Other	0.02	0.45	0.3	3.27	0.33	67.95
Eukaryota;__Holozoa;__Choanomonada;__Acanthoecida;__Diaphanoeca;__	0	0.45	0.3	0.94	0.32	68.27
Bacteria;__Proteobacteria;__Alphaproteobacteria;Other;Other;Other	0.02	0.42	0.3	1.72	0.32	68.59
Bacteria;__Proteobacteria;__Gammaproteobacteria;__Oceanospirillales;__oc58;__g	0.39	0	0.28	2.09	0.31	68.9
Eukaryota;__Alveolata;__Protalveolata;__Syndiniales;__Syndiniales Group II;__	0	0.41	0.28	1.29	0.3	69.2
Bacteria;__WCHB1-60;__c;__o;__f;__g	0.37	0	0.28	0.79	0.3	69.5
Bacteria;__Bacteroidetes;__Flavobacteria;__Flavobacteriales;__Flavobacteriaceae;__NS3a_marine_group	0	0.38	0.28	7.63	0.3	69.81
Bacteria;__Bacteroidetes;__Flavobacteria;__Flavobacteriales;__Flavobacteriaceae;__g	0	0.39	0.28	5.57	0.3	70.11
Unassigned;Other;Other;Other;Other;Other	0.91	1.01	0.27	1.09	0.3	70.4
Bacteria;__Fusobacteria;__Fusobacteria;__Fusobacteriales;__Fusobacteriaceae;__Fusobacterium	0.38	0	0.27	1.35	0.3	70.7
Bacteria;__Proteobacteria;__Deltaproteobacteria;__SAR324_clade(Marine_group_B);__f;__g	0	0.35	0.27	1.32	0.29	70.99
Bacteria;__Proteobacteria;__Betaproteobacteria;__Burkholderiales;__Comamonadaceae;__Acidovorax	0.38	0	0.27	1.31	0.29	71.29
Eukaryota;__Stramenopiles;__MAST-1;__MAST-1 A;__;__	0	0.4	0.27	1.32	0.29	71.58
Bacteria;__Proteobacteria;__Deltaproteobacteria;__Myxococcales;__0319-6G20;__g	0.36	0	0.26	1.57	0.29	71.86
Bacteria;__Bacteroidetes;__Cytophagia;__Cytophagales;__Flammeovirgaceae;__Marinoscillum	0	0.33	0.26	0.9	0.28	72.15
Bacteria;__Verrucomicrobia;__Opitutae;__Opitutales;__Opitutaceae;__Opitutus	0.36	0	0.26	1.47	0.28	72.43
Eukaryota;__Alveolata;__Dinoflagellata;__Dinophyceae;__Gymnodiniphycidae;__Gymnodinium	0	0.39	0.26	0.94	0.28	72.71
Bacteria;__Gemmatimonadetes;__Gemmatimonadetes;__Gemmatimonadales;__Gemmatimonadaceae;__Gemmatimonas	0.35	0	0.25	1.68	0.27	72.98
Bacteria;__Proteobacteria;__Alphaproteobacteria;__SAR11_clade;__f;__g	0	0.32	0.25	1.17	0.27	73.25
Eukaryota;__Alveolata;__Dinoflagellata;__Dinophyceae;__Gymnodiniphycidae;Other	0	0.38	0.25	0.77	0.27	73.51
Bacteria;__Bacteroidetes;__vadinHA17;__o;__f;__g	0.34	0	0.24	2.46	0.26	73.78
Eukaryota;__Alveolata;__Ciliophora;__Spirotrichea;__Choreotrichia;__uncultured	0	0.37	0.24	0.86	0.26	74.04
Eukaryota;__Chloroplastida;__Mamiellophyceae;__Micromonas;__;__	0	0.33	0.23	1.16	0.25	74.29
Bacteria;__Proteobacteria;__Gammaproteobacteria;__Oceanospirillales;Other;Other	0	0.3	0.23	2.32	0.25	74.54
Bacteria;__Candidate_division_OD1;__c;__o;__f;__g	0.3	0	0.23	0.66	0.25	74.79

Bacteria;__Proteobacteria;__Alphaproteobacteria;__Rhodobacterales;__Rhodobacteraceae;__Rhodobacter	0.32	0	0.23	1.17	0.25	75.03
Archaea;__Euryarchaeota;__Halobacteria;__Halobacteriales;__Deep_Sea_Hydrothermal_Vent_Gp_6(DHVEG-6);__Candidatus_Parvarchaeum	0.3	0	0.22	1.06	0.24	75.27
Eukaryota;__Rhizaria;__Cercozoa;__Thecofilosea;__Cryomonadida;__Cryothecomonas	0	0.34	0.22	0.88	0.24	75.51
Eukaryota;__Metazoa;__Cnidaria;__Hydrozoa;__;__	0	0.33	0.21	0.67	0.23	75.74
Bacteria;__Proteobacteria;__Gammaproteobacteria;__Alteromonadales;__Alteromonadaceae;__BD1-7_clade	0.29	0	0.21	1.5	0.22	75.97
Bacteria;__Proteobacteria;__Betaproteobacteria;__Burkholderiales;__Comamonadaceae;__Rhizobacter	0.28	0	0.2	2.37	0.22	76.19
Bacteria;__Proteobacteria;__Betaproteobacteria;__Burkholderiales;__Oxalobacteraceae;__Undibacterium	0.28	0	0.2	3.12	0.22	76.41
Eukaryota;__Stramenopiles;__MAST-8;__;__;__	0	0.29	0.2	1.24	0.22	76.63
Bacteria;__Bacteroidetes;__Cytophagia;__Cytophagales;__Flammeovirgaceae;Other	0	0.27	0.2	1.94	0.22	76.84
Bacteria;__Proteobacteria;__Alphaproteobacteria;__Sphingomonadales;__Sphingomonadaceae;__Sandarakinorhabdus	0.27	0	0.2	1.25	0.21	77.06
Bacteria;__Proteobacteria;__Betaproteobacteria;__Neisseriales;__Neisseriaceae;__g	0.27	0	0.19	2.07	0.21	77.27
Eukaryota;__Fungi;__LKM11;__;__;__	0.26	0	0.19	2.58	0.21	77.47
Eukaryota;__Haptophyta;__Prymnesiophyceae;__Prymnesiales;Other;Other	0	0.27	0.19	1.33	0.2	77.67
Bacteria;__Proteobacteria;__Gammaproteobacteria;__Oceanospirillales;__Oceanospirillaceae;__Balneatrix	0	0.25	0.19	1.62	0.2	77.87
Bacteria;__Proteobacteria;__Alphaproteobacteria;__Rhodospirillales;__Rhodospirillaceae;__Defluviicoccus	0	0.25	0.18	2.79	0.2	78.07
Bacteria;__Bacteroidetes;__Sphingobacteriia;__Sphingobacteriales;__env.OPS_17;__g	0.25	0	0.18	1.81	0.19	78.26
Eukaryota;__Stramenopiles;__T53A1;__;__;__	0.24	0	0.18	1.77	0.19	78.46
Eukaryota;__Metazoa;__Annelida;__;__;__	0	0.27	0.18	1.01	0.19	78.65
Bacteria;__Firmicutes;__Clostridia;__Clostridiales;__Ruminococcaceae;__g	0.24	0	0.17	1.48	0.19	78.84
Bacteria;__Bacteroidetes;__Bacteroidia;__Bacteroidales;__Prevotellaceae;__Prevotella	0.24	0	0.17	1.04	0.19	79.02
Bacteria;__Verrucomicrobia;__Opitutae;__MB11C04_marine_group;__f;__g	0	0.24	0.17	2.42	0.19	79.21
Bacteria;__Proteobacteria;__Alphaproteobacteria;__Sphingomonadales;__Sphingomonadaceae;__Sphingopyxis	0.02	0.26	0.17	2.97	0.18	79.39
Bacteria;__Firmicutes;__Bacilli;__Bacillales;__Staphylococcaceae;Other	0.23	0	0.17	1.33	0.18	79.57
Eukaryota;__Fungi;Other;Other;Other;Other	0.23	0	0.17	1.78	0.18	79.75
Bacteria;__Proteobacteria;__Betaproteobacteria;__Burkholderiales;__Comamonadaceae;__Comamonas	0.22	0	0.16	1.08	0.17	79.92
Eukaryota;__Stramenopiles;__Chrysophyceae;Other;Other;Other	0.22	0	0.16	3.25	0.17	80.1

Groups Atlas Pool & Winston Lagoon

Average dissimilarity = 84.87

Species	Atlas Pool Av.Abund	Winston Lagoon Av.Abund	Av.Diss	Diss/SD	Contrib%	Cum.%
Eukaryota;__Alveolata;__Ciliophora;__Spirotrichea;__Hypotrichia;Other	3.49	0.3	2.61	2.5	3.07	3.07
Bacteria;__Cyanobacteria;__Chloroplast;__Chloroplast;__Chloroplast;__Chloroplast	3.27	3.37	2.09	1.27	2.46	5.54
Bacteria;__Actinobacteria;__Actinobacteria;__Frankiales;__Sporichthyaceae;__hgcI_clade	2.32	0	1.99	0.92	2.34	7.88
Bacteria;__Proteobacteria;__Alphaproteobacteria;__SAR11_clade;__Surface_1;__g	0	2.12	1.75	0.77	2.06	9.94
Eukaryota;__Chloroplastida;__Chlorophyceae;__Chlamydomonas;__;__	2.25	0	1.7	1.66	2	11.94
Bacteria;__Bacteroidetes;__Flavobacteria;__Flavobacteriales;__Flavobacteriaceae;__Flavobacterium	2.2	0	1.7	2.47	2	13.94
Bacteria;__Bacteroidetes;__Flavobacteria;__Flavobacteriales;__Flavobacteriaceae;Other	0.03	2.34	1.68	0.96	1.98	15.92
Bacteria;__Actinobacteria;__Actinobacteria;__Micrococcales;__Microbacteriaceae;Other	1.99	0	1.66	1.17	1.96	17.88
Bacteria;__Proteobacteria;__Betaproteobacteria;__Burkholderiales;__Comamonadaceae;Other	2.66	1.03	1.62	1.53	1.91	19.79
Bacteria;__Proteobacteria;__Alphaproteobacteria;__Sphingomonadales;__Sphingomonadaceae;__Sphingopyxis	0	1.75	1.5	0.78	1.77	21.56
Bacteria;__Proteobacteria;__Betaproteobacteria;__Burkholderiales;__Burkholderiaceae;__Polynucleobacter	1.75	0	1.48	1.06	1.74	23.3
Eukaryota;__Chloroplastida;__Chlorophyceae;Other;Other;Other	1.85	0.04	1.36	1.45	1.6	24.9
Archaea;__Thaumarchaeota;__Marine_Group_I;__o;__f;__g	0	1.55	1.27	0.93	1.5	26.39
Bacteria;__Proteobacteria;__Gammaproteobacteria;__Oceanospirillales;__SAR86_clade;__g	0	1.39	1.14	1.02	1.34	27.74
Bacteria;__Proteobacteria;__Gammaproteobacteria;__Oceanospirillales;__ZD0405;__g	0	1.34	1.1	1.01	1.3	29.03
Bacteria;__Proteobacteria;__Alphaproteobacteria;__Rhodobacterales;__Rhodobacteraceae;Other	1.03	1.87	1.04	1.69	1.23	30.26
Bacteria;__Proteobacteria;__Gammaproteobacteria;__Alteromonadales;__Pseudoalteromonadaceae;__g	0	1.15	0.88	1.65	1.03	31.3
Bacteria;__Proteobacteria;__Betaproteobacteria;__Burkholderiales;__Alcaligenaceae;__Achromobacter	1.09	0	0.87	2.19	1.03	32.32
Bacteria;__Actinobacteria;__Actinobacteria;__Frankiales;__Sporichthyaceae;Other	1.02	0	0.87	0.94	1.03	33.35
Bacteria;__Proteobacteria;__Betaproteobacteria;__Burkholderiales;__Comamonadaceae;__Polaromonas	1.23	0.11	0.86	1.78	1.02	34.37
Eukaryota;__Rhizaria;__Cercozoa;__Endomyxa;__Novel Clade 10;__	1.09	0	0.84	2.1	0.99	35.36
Archaea;__Euryarchaeota;__Halobacteria;__Halobacteriales;__Deep_Sea_Hydrothermal_Vent_Gp_6(DHVEG-6);__Candidatus_Parvarchaeum	0.19	1.11	0.82	0.82	0.97	36.33
Bacteria;__Bacteroidetes;__Flavobacteria;__Flavobacteriales;__Flavobacteriaceae;__NS3a_marine_group	0	0.9	0.76	0.89	0.9	37.23
Bacteria;__Actinobacteria;__Actinobacteria;__Corynebacteriales;__Nocardiaceae;__Rhodococcus	0	0.96	0.71	1.1	0.84	38.07
Bacteria;__Proteobacteria;__Gammaproteobacteria;__Alteromonadales;__Colwelliaceae;__Colwellia	0.05	0.95	0.7	1.61	0.82	38.89
Bacteria;__Proteobacteria;__Betaproteobacteria;__Burkholderiales;__Comamonadaceae;__Simplicispira	0.91	0	0.69	1.98	0.81	39.7
Bacteria;__Bacteroidetes;__Flavobacteria;__Flavobacteriales;__Flavobacteriaceae;__Polaribacter	0	0.91	0.68	1.26	0.8	40.5
Eukaryota;__Chloroplastida;__Mamiellophyceae;__Micromonas;__;__	0	0.8	0.68	0.93	0.8	41.3

Bacteria;__Proteobacteria;__Alphaproteobacteria;__Rhodobacterales;__Rhodobacteraceae;__g	0.03	0.89	0.65	1.34	0.76	42.06
Bacteria;__Actinobacteria;__Actinobacteria;__PeM15;__f;__g	0.79	0.04	0.63	1.16	0.74	42.8
Eukaryota;__Fungi;__Chytridiomycota;__;__;__	0.86	0.11	0.56	1.48	0.66	43.46
Archaea;__Euryarchaeota;__Halobacteria;__Halobacteriales;__Deep_Sea_Hydrothermal_Vent_Gp_6(DHVEG-6);__g	0.29	0.68	0.56	1.03	0.66	44.12
Eukaryota;__Alveolata;__Ciliophora;__Conthreep;__Phyllopharyngea;__AT1-2	0	0.75	0.55	0.9	0.64	44.76
Bacteria;__Proteobacteria;__Alphaproteobacteria;__SAR11_clade;Other;Other	0	0.61	0.51	0.78	0.6	45.36
Bacteria;__Bacteroidetes;__Flavobacteria;__Flavobacteriales;__Flavobacteriaceae;__g	0.03	0.68	0.49	0.9	0.58	45.94
Bacteria;__Candidate_division_OP11;__c;__o;__f;__g	0.55	0.19	0.46	1.01	0.54	46.48
Bacteria;__Actinobacteria;__Acidimicrobiia;__Acidimicrobiales;__Acidimicrobiaceae;__g	0.04	0.54	0.46	0.73	0.54	47.02
Bacteria;__Proteobacteria;__Alphaproteobacteria;__Rhodobacterales;__Rhodobacteraceae;__Roseobacter_clade_DC5-80-3_lineage	0	0.55	0.45	2.79	0.53	47.55
Eukaryota;__Rhizaria;__Cercozoa;__Endomyxa;__Phytomyxea;__Spongospora	0.59	0	0.45	2.47	0.53	48.08
Bacteria;__Actinobacteria;__Actinobacteria;__Micrococcales;__Microbacteriaceae;__Candidatus_Aquiluna	0.53	0	0.44	1.41	0.52	48.6
Bacteria;__Proteobacteria;__Betaproteobacteria;__Burkholderiales;__Comamonadaceae;__Paucibacter	0.61	0.04	0.44	1.66	0.51	49.11
Eukaryota;__Stramenopiles;__Diatomea;__Bacillariophytina;__Mediophyceae;Other	0.58	0	0.43	1.32	0.51	49.62
Eukaryota;__Alveolata;__Ciliophora;__Conthreep;__Phyllopharyngea;__Dysteria	0	0.59	0.42	0.78	0.5	50.12
Bacteria;__Proteobacteria;__Deltaproteobacteria;__Bdellovibrionales;__Bacteriovoracaceae;__Peredibacter	0.05	0.59	0.42	1.47	0.5	50.62
Bacteria;__Candidate_division_OD1;__c;__o;__f;__g	0.2	0.56	0.42	0.9	0.49	51.11
Bacteria;__Proteobacteria;__Betaproteobacteria;Other;Other;Other	0.64	0.68	0.41	1.49	0.48	51.59
Bacteria;__Proteobacteria;__Betaproteobacteria;__Burkholderiales;__Alcaligenaceae;Other	0.57	0.07	0.39	1.84	0.46	52.05
Bacteria;__Actinobacteria;__Actinobacteria;__Micrococcales;__Microbacteriaceae;__Candidatus_Limnoluna	0.45	0	0.38	1.19	0.44	52.49
Bacteria;__Bacteroidetes;__Sphingobacteriia;__Sphingobacteriales;__Saprospiraceae;__Lewinella	0	0.48	0.36	1.08	0.42	52.91
Unassigned;Other;Other;Other;Other;Other	1.1	1.43	0.35	1.48	0.42	53.33
Bacteria;__Proteobacteria;__Betaproteobacteria;__Burkholderiales;__Comamonadaceae;__g	0.45	0.04	0.34	1.31	0.4	53.73
Bacteria;__Bacteroidetes;__Flavobacteria;__Flavobacteriales;__Flavobacteriaceae;__NS2b_marine_group	0	0.42	0.34	7.52	0.4	54.12
Bacteria;__Proteobacteria;__Betaproteobacteria;__Burkholderiales;Other;Other	0.38	0	0.32	0.98	0.38	54.5
Eukaryota;__Chloroplastida;__Trebouxiophyceae;__;__;__	0.4	0	0.31	2.01	0.36	54.87
Bacteria;__Proteobacteria;__Gammaproteobacteria;__Pseudomonadales;__Moraxellaceae;__Psychrobacter	0.5	0.16	0.31	1.53	0.36	55.23
Archaea;__Euryarchaeota;__Thermoplasmata;__Thermoplasmatales;__Marine_Group_II;__g	0	0.37	0.3	2.66	0.35	55.58
Bacteria;__Proteobacteria;__Gammaproteobacteria;__Xanthomonadales;__Xanthomonadaceae;__Arenimonas	0.38	0	0.3	2.12	0.35	55.93
Bacteria;__Proteobacteria;__Alphaproteobacteria;__SAR11_clade;__Surface_4;__g	0	0.36	0.3	0.66	0.35	56.28

Bacteria;__Bacteroidetes;__Flavobacteria;__Flavobacteriales;__Flavobacteriaceae;__NS4_marine_group	0	0.36	0.29	2.95	0.34	56.62
Bacteria;__Proteobacteria;__Gammaproteobacteria;__Alteromonadales;__Alteromonadaceae;__SAR92_clade	0	0.35	0.29	2.7	0.34	56.96
Bacteria;__Bacteroidetes;__Sphingobacteriia;__Sphingobacteriales;__NS11-12_marine_group;__g	0.41	0.35	0.29	1.4	0.34	57.3
Eukaryota;__Stramenopiles;__T53A1;__;__;__	0.37	0	0.29	1.98	0.34	57.64
Bacteria;__Proteobacteria;__Gammaproteobacteria;__Oceanospirillales;__Oceanospirillaceae;Other	0	0.36	0.28	2.73	0.33	57.97
Bacteria;__Proteobacteria;__Alphaproteobacteria;__Rhodobacterales;__Rhodobacteraceae;__Loktanella	0	0.36	0.28	1.29	0.33	58.3
Eukaryota;__Stramenopiles;__Bicosoecida;__Siluaniidae;__Siluania;__	0.36	0	0.28	1.78	0.33	58.63
Bacteria;__Proteobacteria;__Betaproteobacteria;__Burkholderiales;__Oxalobacteraceae;__Undibacterium	0.36	0	0.27	1.75	0.32	58.95
Bacteria;__Proteobacteria;__Gammaproteobacteria;__Alteromonadales;__Alteromonadaceae;__Glaciecola	0	0.36	0.27	1.02	0.31	59.26
Bacteria;__Bacteroidetes;__Flavobacteria;__Flavobacteriales;__Flavobacteriaceae;__NS5_marine_group	0	0.32	0.26	1.33	0.31	59.57
Bacteria;__Bacteroidetes;__Sphingobacteriia;__Sphingobacteriales;__Saprospiraceae;__g	0.07	0.34	0.26	0.83	0.31	59.88
Eukaryota;__Alveolata;__Ciliophora;__Spirotrichea;__Hypotrichia;__Gonostomum	0.29	0	0.26	0.66	0.3	60.18
Eukaryota;__Alveolata;__Protalveolata;__Colpodellida;Other;Other	0.33	0	0.25	1.25	0.29	60.47
Bacteria;__Bacteroidetes;__Flavobacteria;__Flavobacteriales;__Cryomorphaceae;__Owenweeksia	0.07	0.39	0.25	1.7	0.29	60.76
Bacteria;__Proteobacteria;__Gammaproteobacteria;__Enterobacteriales;__Enterobacteriaceae;Other	0.07	0.37	0.25	1.21	0.29	61.05
Bacteria;__Proteobacteria;__Gammaproteobacteria;Other;Other;Other	0.09	0.38	0.24	1.71	0.29	61.34
Bacteria;__Bacteroidetes;__Bacteroidia;__Bacteroidales;__Bacteroidaceae;__Bacteroides	0.31	0.3	0.24	1.63	0.28	61.63
Bacteria;__Bacteroidetes;__Flavobacteria;__Flavobacteriales;__NS9_marine_group;__g	0.09	0.4	0.24	1.22	0.28	61.91
Bacteria;__Proteobacteria;__Betaproteobacteria;__Burkholderiales;__Comamonadaceae;__Albidiferax	0.3	0	0.24	6.99	0.28	62.19
Bacteria;__Actinobacteria;__Actinobacteria;__Micrococcales;__Microbacteriaceae;__Candidatus_Planktoluna	0.28	0	0.23	1.07	0.28	62.46
Bacteria;__Bacteroidetes;__Sphingobacteriia;__Sphingobacteriales;__Saprospiraceae;__Aureispira	0	0.32	0.23	0.82	0.27	62.74
Eukaryota;__Alveolata;__Ciliophora;__Spirotrichea;__Hypotrichia;__	0	0.31	0.23	1.57	0.27	63.01
Bacteria;__Proteobacteria;__Gammaproteobacteria;__BD7-8_marine_group;__f;__g	0	0.29	0.23	1.17	0.27	63.28
Eukaryota;__Chloroplastida;__Ulvophyceae;Other;Other;Other	0	0.31	0.23	1.11	0.27	63.55
Bacteria;__Fusobacteria;__Fusobacteria;__Fusobacteriales;__Fusobacteriaceae;__Fusobacterium	0.36	0.36	0.23	1.3	0.27	63.81
Bacteria;__Proteobacteria;__Alphaproteobacteria;__Rhodobacterales;__Rhodobacteraceae;__Rhodobacter	0.33	0.11	0.23	1.98	0.27	64.08
Bacteria;__Proteobacteria;__Alphaproteobacteria;__Rickettsiales;__mitochondria;__g	0.37	0.1	0.23	1.63	0.27	64.35
Bacteria;__Firmicutes;__Clostridia;__Clostridiales;__Ruminococcaceae;__g	0.35	0.21	0.22	1.35	0.26	64.61
Bacteria;__Bacteroidetes;__Cytophagia;__Cytophagales;__Flammeovirgaceae;__Reichenbachiella	0	0.29	0.22	2.28	0.26	64.87
Bacteria;__Proteobacteria;__Gammaproteobacteria;__Alteromonadales;__Shewanellaceae;__Shewanella	0	0.28	0.22	2.9	0.26	65.13
Bacteria;__Proteobacteria;__Deltaproteobacteria;__Myxococcales;__0319-6G20;__g	0.41	0.15	0.22	1.37	0.26	65.39
Bacteria;__Candidate_division_TM7;__c;__o;__f;__g	0.23	0.19	0.22	1.99	0.26	65.65

Bacteria;__Proteobacteria;__Alphaproteobacteria;__Kordiimonadales;__Kordiimonadaceae;__Kordiimonas	0	0.28	0.22	1.73	0.25	65.9
Eukaryota;__Alveolata;__Ciliophora;__Spirotrichea;__Oligotrichia;__Strombidium	0	0.27	0.21	2.29	0.25	66.15
Bacteria;__Proteobacteria;__Gammaproteobacteria;__Pseudomonadales;__Moraxellaceae;__Perlucidibaca	0.27	0	0.21	2.09	0.24	66.4
Eukaryota;__Stramenopiles;__Chrysophyceae;__Chromulinales;__Phaeoplaca;__	0.26	0	0.2	6.58	0.24	66.64
Bacteria;__Proteobacteria;__Gammaproteobacteria;__Oceanospirillales;__oc58;__g	0.27	0	0.2	1.32	0.24	66.88
Eukaryota;__Chloroplastida;__Chlorophyceae;__Desmodesmus;__;__	0.27	0	0.2	1.31	0.24	67.11
Eukaryota;__Alveolata;__Ciliophora;__Spirotrichea;__Oligotrichia;__uncultured	0	0.26	0.2	3.11	0.24	67.35
Bacteria;__Bacteroidetes;__Sphingobacteriia;__Sphingobacteriales;__Chitinophagaceae;__g	0.28	0.13	0.2	1.14	0.23	67.58
Bacteria;__Proteobacteria;__Deltaproteobacteria;__Bdellovibrionales;__Bacteriovoracaceae;__Bacteriovorax	0.24	0	0.2	1.59	0.23	67.82
Bacteria;__Proteobacteria;__Alphaproteobacteria;__Rickettsiales;__SAR116_clade;__g	0	0.24	0.2	1.22	0.23	68.05
Bacteria;__Proteobacteria;__Betaproteobacteria;__Burkholderiales;__Comamonadaceae;__Hydrogenophaga	0.26	0	0.2	1.1	0.23	68.28
Bacteria;__Bacteroidetes;__Sphingobacteriia;__Sphingobacteriales;__Chitinophagaceae;__Filimonas	0.26	0	0.2	1.21	0.23	68.51
Bacteria;__Proteobacteria;__Alphaproteobacteria;__SAR11_clade;__f;__g	0	0.24	0.2	0.85	0.23	68.74
Bacteria;__Proteobacteria;__Gammaproteobacteria;__Alteromonadales;Other;Other	0	0.24	0.19	1.87	0.23	68.97
Bacteria;__Bacteroidetes;__Flavobacteria;__Flavobacteriales;__Flavobacteriaceae;__Pibocella	0	0.26	0.19	0.96	0.23	69.2
Eukaryota;__Alveolata;__Ciliophora;__Conthreep;__Oligohymenophorea;Other	0.27	0.06	0.19	1.17	0.23	69.43
Bacteria;__Proteobacteria;__Gammaproteobacteria;__Alteromonadales;__Moritellaceae;__Moritella	0	0.26	0.19	1.13	0.23	69.66
Bacteria;__Proteobacteria;__Gammaproteobacteria;__Alteromonadales;__Alteromonadaceae;Other	0	0.25	0.19	3.72	0.23	69.88
Bacteria;__Bacteroidetes;__Cytophagia;__Cytophagales;__Cytophagaceae;__Leadbetterella	0.14	0.18	0.19	1.41	0.22	70.1
Bacteria;__Proteobacteria;__Betaproteobacteria;__Burkholderiales;__Burkholderiaceae;__Ralstonia	0.23	0	0.19	1.09	0.22	70.33
Bacteria;__Proteobacteria;__Gammaproteobacteria;__Thiotrichales;__Thiotrichaceae;__Thiothrix	0	0.23	0.19	0.66	0.22	70.55
Eukaryota;__Alveolata;__Protalveolata;__Perkinsidae;__NA2-2D10;__	0	0.23	0.18	1.16	0.22	70.76
Bacteria;__Proteobacteria;__Gammaproteobacteria;__Chromatiales;__Granulosicoccaceae;__Granulosicoccus	0	0.24	0.18	1.31	0.21	70.98
Bacteria;__Bacteroidetes;__Sphingobacteriia;__Sphingobacteriales;__WCHB1-69;__g	0.24	0	0.18	1.18	0.21	71.19
Bacteria;__Proteobacteria;__Betaproteobacteria;__Rhodocyclales;__Rhodocyclaceae;__Dechloromonas	0.23	0	0.18	2.87	0.21	71.4
Bacteria;__Proteobacteria;__Betaproteobacteria;__Burkholderiales;__Alcaligenaceae;__Pusillimonas	0.2	0	0.18	0.66	0.21	71.61
Bacteria;__Proteobacteria;__Alphaproteobacteria;Other;Other;Other	0	0.21	0.17	0.88	0.21	71.81
Eukaryota;__Alveolata;__Ciliophora;__Spirotrichea;__Euplotia;__Aspidisca	0.03	0.26	0.17	1.48	0.2	72.02
Bacteria;__Planctomycetes;__BD7-11;__o;__f;__g	0	0.21	0.17	2.02	0.2	72.22
Bacteria;__Bacteroidetes;__Flavobacteria;__Flavobacteriales;__Cryomorphaceae;__Fluviicola	0.14	0.31	0.17	1.16	0.2	72.42

Bacteria;__Proteobacteria;__Gammaproteobacteria;__Oceanospirillales;__Oceanospirillaceae;__Neptunomonas	0	0.23	0.17	0.89	0.2	72.62
Bacteria;__Proteobacteria;__Alphaproteobacteria;__Sphingomonadales;__Erythrobacteraceae;__Altererythrobacter	0	0.2	0.17	0.66	0.2	72.82
Bacteria;__Actinobacteria;__Actinobacteria;__Micrococcales;__Microbacteriaceae;__Alpinimonas	0.19	0	0.17	0.66	0.2	73.02
Eukaryota;__Rhizaria;__Cercozoa;__Thecofilosea;Other;Other	0.08	0.2	0.17	1.03	0.2	73.22
Bacteria;__Proteobacteria;__Alphaproteobacteria;__Rhodospirillales;__Rhodospirillaceae;__Defluviicoccus	0	0.2	0.17	0.66	0.2	73.42
Bacteria;__Proteobacteria;__Gammaproteobacteria;__Thiotrichales;__Thiotrichaceae;__Leucothrix	0	0.23	0.17	0.9	0.2	73.61
Eukaryota;__Chloroplastida;__Chlorophyceae;__;__;__	0.22	0	0.17	1.17	0.19	73.81
Eukaryota;__Alveolata;__Ciliophora;__Conthreep;__Prostomatea;__Cryptocaryon	0.26	0.08	0.16	1.79	0.19	74
Bacteria;__Candidate_division_OP3;__c;__o;__f;__g	0.11	0.23	0.16	1.04	0.19	74.19
Bacteria;__Proteobacteria;__Gammaproteobacteria;__Order_Incertae_Sedis;__Family_Incertae_Sedis;__Arenicella	0	0.21	0.16	1.15	0.19	74.38
Bacteria;__WCHB1-60;__c;__o;__f;__g	0.18	0	0.16	0.76	0.19	74.56
Bacteria;__Bacteroidetes;__Bacteroidia;__Bacteroidales;__Marinilabiaceae;__Marinifilum	0	0.22	0.16	0.91	0.19	74.75
Bacteria;__Proteobacteria;__Gammaproteobacteria;__Xanthomonadales;__Xanthomonadaceae;Other	0.21	0	0.16	1.13	0.19	74.93
Bacteria;__Proteobacteria;__Gammaproteobacteria;__Pseudomonadales;__Pseudomonadaceae;__Pseudomonas	0.12	0.3	0.16	1.26	0.18	75.12
Eukaryota;__Rhizaria;__Cercozoa;Other;Other;Other	0.19	0.21	0.16	1.51	0.18	75.3
Bacteria;__Proteobacteria;__Alphaproteobacteria;__Rhodobacterales;__Rhodobacteraceae;__Sulfitobacter	0	0.22	0.16	0.66	0.18	75.49
Bacteria;__Firmicutes;__Bacilli;__Bacillales;__Staphylococcaceae;Other	0.2	0	0.15	1.29	0.18	75.66
Archaea;__Euryarchaeota;__Methanomicrobia;__Methanomicrobiales;__Methanoregulaceae;__Methanoregula	0.19	0	0.15	2.05	0.17	75.84
Bacteria;__Proteobacteria;__Betaproteobacteria;__Burkholderiales;__Comamonadaceae;__Acidovorax	0.13	0.14	0.15	1.85	0.17	76.01
Bacteria;__Firmicutes;__Clostridia;__Clostridiales;__Ruminococcaceae;__Faecalibacterium	0.11	0.16	0.14	1.27	0.17	76.18
Bacteria;__Proteobacteria;__Alphaproteobacteria;__Rhodospirillales;__Rhodospirillaceae;__Thalassospira	0	0.17	0.14	1.27	0.17	76.35
Eukaryota;__Fungi;__Blastocladiomycota;__;__;__	0.19	0	0.14	1.16	0.17	76.52
Eukaryota;__Stramenopiles;__Diatomea;__Coscinodiscophytina;__Fragilariales;__	0	0.19	0.14	0.95	0.17	76.68
Bacteria;__Proteobacteria;__Gammaproteobacteria;__Oceanospirillales;__Oceanospirillaceae;__Marinomonas	0	0.17	0.14	1.07	0.16	76.84
Bacteria;__Bacteroidetes;__Bacteroidia;__Bacteroidales;__Prevotellaceae;__Prevotella	0.19	0.21	0.14	1.42	0.16	77
Bacteria;__Proteobacteria;__Gammaproteobacteria;__Xanthomonadales;__Sinobacteraceae;__g	0.17	0	0.14	4.55	0.16	77.16
Bacteria;__Bacteroidetes;__Flavobacteria;__Flavobacteriales;__Flavobacteriaceae;__Chryseobacterium	0.18	0	0.14	1.31	0.16	77.32
Bacteria;__Proteobacteria;__Deltaproteobacteria;__Syntrophobacterales;__Syntrophaceae;__Smithella	0.17	0	0.13	6.45	0.16	77.48

Bacteria;__Lentisphaerae;__Lentisphaeria;__Lentisphaerales;__Lentisphaeraceae;__Lentisphaera	0	0.18	0.13	0.97	0.16	77.64
Bacteria;__Actinobacteria;__Acidimicrobiia;__Acidimicrobiales;__OCS155_marine_group;__g	0	0.16	0.13	0.66	0.16	77.8
Bacteria;__Proteobacteria;__Betaproteobacteria;__Burkholderiales;__Comamonadaceae;__Pelomonas	0.17	0	0.13	1.06	0.16	77.95
Bacteria;__Proteobacteria;__Deltaproteobacteria;__Desulfobacterales;__Nitrospinaceae;__Nitrospina	0	0.16	0.13	1.15	0.15	78.11
Bacteria;__Proteobacteria;__Gammaproteobacteria;__Alteromonadales;__Alteromonadaceae;__BD1-7_clade	0.18	0	0.13	1.28	0.15	78.26
Eukaryota;__Alveolata;__Ciliophora;__Conthreep;__Phyllopharyngea;__Hartmannula	0	0.18	0.13	0.66	0.15	78.41
Bacteria;__Bacteroidetes;__Sphingobacteriia;__Sphingobacteriales;__Chitinophagaceae;__Ferruginibacter	0.17	0	0.13	1.28	0.15	78.56
Eukaryota;__Fungi;Other;Other;Other;Other	0.17	0	0.13	1.3	0.15	78.71
Bacteria;__Bacteroidetes;__Cytophagia;__Cytophagales;__Flammeovirgaceae;__Marinoscillum	0	0.16	0.12	1.3	0.15	78.86
Bacteria;__Proteobacteria;__Gammaproteobacteria;__Legionellales;__Coxiellaceae;__Coxiella	0	0.16	0.12	0.96	0.15	79.01
Eukaryota;__Alveolata;__Ciliophora;__Conthreep;__Nassophorea;__	0	0.17	0.12	0.99	0.15	79.15
Bacteria;__Bacteroidetes;__vadinHA17;__o;__f;__g	0.16	0	0.12	2.9	0.15	79.3
Bacteria;__Proteobacteria;__Deltaproteobacteria;__Sh765B-TzT-29;__f;__g	0	0.15	0.12	0.66	0.14	79.44
Eukaryota;__Alveolata;__Dinoflagellata;__Dinophyceae;__Gymnodiniphycidae;__FV18-2D9	0.16	0	0.12	1.29	0.14	79.59
Bacteria;__Actinobacteria;Other;Other;Other;Other	0.15	0	0.12	1.02	0.14	79.73
Eukaryota;__Stramenopiles;__Eustigmatales;__Nannochloropsis;__;__	0.16	0	0.12	1.31	0.14	79.87
Bacteria;__Bacteroidetes;__Sphingobacteriia;__Sphingobacteriales;__Sphingobacteriaceae;__Pedobacter	0.17	0	0.12	0.86	0.14	80.02

Groups Seal Pool & Winston Lagoon

Average dissimilarity = 85.12

Species	Seal Pool Av.Abund	Winston Lagoon Av.Abund	Av.Diss	Diss/SD	Contrib%	Cum.%
Bacteria;__Actinobacteria;__Actinobacteria;__PeM15;__f;__g	3.86	0.04	2.92	1.63	3.44	3.44
Bacteria;__Bacteroidetes;__Flavobacteria;__Flavobacteriales;__Flavobacteriaceae;__Flavobacterium	3.15	0	2.36	1.94	2.77	6.2
Bacteria;__Cyanobacteria;__Chloroplast;__Chloroplast;__Chloroplast;__Chloroplast	0.84	3.37	2.13	1.14	2.5	8.7
Bacteria;__Proteobacteria;__Alphaproteobacteria;__SAR11_clade;__Surface_1;__g	0.06	2.12	1.61	0.76	1.89	10.59
Bacteria;__Bacteroidetes;__Flavobacteria;__Flavobacteriales;__Flavobacteriaceae;Other	0.05	2.34	1.59	0.95	1.87	12.46
Bacteria;__Proteobacteria;__Betaproteobacteria;__Burkholderiales;__Burkholderiaceae;__Polynucleobacter	2.03	0	1.57	1.17	1.85	14.31
Bacteria;__Proteobacteria;__Betaproteobacteria;__Burkholderiales;__Comamonadaceae;Other	2.78	1.03	1.57	1.68	1.84	16.15
Bacteria;__Proteobacteria;__Betaproteobacteria;__Burkholderiales;__Comamonadaceae;__Polaromonas	2	0.11	1.42	2.1	1.67	17.82

Bacteria;__Proteobacteria;__Alphaproteobacteria;__Sphingomonadales;__Sphingomonadaceae;__Sphingopyxis	0.02	1.75	1.4	0.78	1.64	19.47
Archaea;__Thaumarchaeota;__Marine_Group_I;__o;__f;__g	0	1.55	1.21	0.93	1.42	20.88
Bacteria;__Proteobacteria;__Alphaproteobacteria;__Rhodobacterales;__Rhodobacteraceae;Other	1.79	1.87	1.04	1.25	1.22	22.1
Bacteria;__Proteobacteria;__Gammaproteobacteria;__Oceanospirillales;__SAR86_clade;__g	0.06	1.39	1.04	0.98	1.22	23.32
Bacteria;__Proteobacteria;__Gammaproteobacteria;__Oceanospirillales;__ZD0405;__g	0.08	1.34	0.99	0.96	1.16	24.48
Bacteria;__Actinobacteria;__Actinobacteria;__Frankiales;__Sporichthyaceae;__hgcI_clade	1.22	0	0.94	1.08	1.11	25.59
Bacteria;__Proteobacteria;__Betaproteobacteria;__Burkholderiales;__Comamonadaceae;__Simplicispira	1.25	0	0.93	1.67	1.09	26.68
Bacteria;__Actinobacteria;__Actinobacteria;__Micrococcales;__Microbacteriaceae;Other	1.18	0	0.91	1.34	1.07	27.75
Bacteria;__Bacteroidetes;__Sphingobacteriia;__Sphingobacteriales;__Chitinophagaceae;__Sediminibacterium	1.15	0	0.86	2.52	1.01	28.76
Bacteria;__Proteobacteria;__Gammaproteobacteria;__Alteromonadales;__Pseudoalteromonadaceae;__g	0	1.15	0.84	1.66	0.98	29.75
Bacteria;__Proteobacteria;__Betaproteobacteria;__Burkholderiales;__Comamonadaceae;__g	1.15	0.04	0.83	1.33	0.97	30.72
Bacteria;__Bacteroidetes;__Sphingobacteriia;__Sphingobacteriales;__Sphingobacteriaceae;__Pedobacter	1.06	0	0.8	1.94	0.95	31.66
Bacteria;__Actinobacteria;__Actinobacteria;__Frankiales;__Sporichthyaceae;Other	1.02	0	0.79	1.06	0.92	32.59
Eukaryota;__Chloroplastida;__Chlorophyceae;__Desmodesmus;__;__	1.02	0	0.77	1.12	0.9	33.49
Archaea;__Euryarchaeota;__Halobacteria;__Halobacteriales;__Deep_Sea_Hydrothermal_Vent_Gp_6(DHVEG-6);__Candidatus_Parvarchaeum	0.3	1.11	0.77	0.84	0.9	34.39
Eukaryota;__Rhizaria;__Cercozoa;__Thecofilosea;Other;Other	1.1	0.2	0.74	1.63	0.87	35.26
Bacteria;__Bacteroidetes;__Flavobacteria;__Flavobacteriales;__Flavobacteriaceae;__NS3a_marine_group	0	0.9	0.72	0.9	0.85	36.1
Bacteria;__Bacteroidetes;__Sphingobacteriia;__Sphingobacteriales;__NS11-12_marine_group;__g	1.14	0.35	0.7	1.26	0.82	36.92
Bacteria;__Proteobacteria;__Gammaproteobacteria;__Alteromonadales;__Colwelliaceae;__Colwellia	0	0.95	0.7	1.75	0.82	37.74
Bacteria;__Proteobacteria;__Betaproteobacteria;__Burkholderiales;__Comamonadaceae;__Paucibacter	0.97	0.04	0.69	1.18	0.81	38.55
Bacteria;__Actinobacteria;__Actinobacteria;__Corynebacteriales;__Nocardiaceae;__Rhodococcus	0	0.96	0.68	1.11	0.8	39.35
Bacteria;__Proteobacteria;__Alphaproteobacteria;__Rhodobacterales;__Rhodobacteraceae;__g	0	0.89	0.64	1.42	0.76	40.11
Eukaryota;__Chloroplastida;__Mamiellophyceae;__Micromonas;__;__	0	0.8	0.64	0.94	0.75	40.86
Bacteria;__Bacteroidetes;__Sphingobacteriia;__Sphingobacteriales;__Chitinophagaceae;__Ferruginibacter	0.86	0	0.64	1.52	0.75	41.61
Bacteria;__Bacteroidetes;__Flavobacteria;__Flavobacteriales;__Flavobacteriaceae;__Polaribacter	0.06	0.91	0.6	1.15	0.7	42.31
Bacteria;__Actinobacteria;Other;Other;Other;Other	0.78	0	0.6	1.66	0.7	43.02
Bacteria;__Bacteroidetes;__Sphingobacteriia;__Sphingobacteriales;__Saprospiraceae;__g	0.76	0.34	0.57	1.11	0.67	43.69
Bacteria;__Proteobacteria;__Betaproteobacteria;__Burkholderiales;__Comamonadaceae;__Albidiferax	0.77	0	0.57	1.47	0.67	44.36
Bacteria;__Proteobacteria;__Gammaproteobacteria;__Pseudomonadales;__Moraxellaceae;__Perlucidibaca	0.76	0	0.57	2.73	0.67	45.02
Archaea;__Euryarchaeota;__Halobacteria;__Halobacteriales;__Deep_Sea_Hydrothermal_Vent_Gp_6(DHVEG-6);__g	0.41	0.68	0.55	1.1	0.64	45.67
Bacteria;__Proteobacteria;__Alphaproteobacteria;__Rhodospirillales;__Acetobacteraceae;__Roseococcus	0.71	0	0.53	1.38	0.62	46.29

Eukaryota;__Alveolata;__Ciliophora;__Conthreep;__Phyllopharyngea;__AT1-2	0	0.75	0.52	0.9	0.61	46.9
Bacteria;__Proteobacteria;__Betaproteobacteria;__Burkholderiales;__Alcaligenaceae;__Achromobacter	0.67	0	0.51	2.44	0.6	47.5
Bacteria;__Proteobacteria;__Alphaproteobacteria;__Sphingomonadales;__Sphingomonadaceae;__Novosphingobium	0.68	0	0.51	2.09	0.6	48.1
Bacteria;__Candidate_division_OP11;__c;__o;__f;__g	0.72	0.19	0.49	1.16	0.58	48.68
Bacteria;__Bacteroidetes;__Sphingobacteriia;__Sphingobacteriales;__Chitinophagaceae;__g	0.71	0.13	0.49	1.18	0.57	49.25
Bacteria;__Bacteroidetes;__Flavobacteria;__Flavobacteriales;__Flavobacteriaceae;__g	0	0.68	0.48	0.9	0.56	49.81
Bacteria;__Actinobacteria;__Actinobacteria;__Micrococcales;__Microbacteriaceae;__Candidatus_Aquiluna	0.62	0	0.47	1.62	0.56	50.37
Bacteria;__Bacteroidetes;__Flavobacteria;__Flavobacteriales;__Cryomorphaceae;__Fluviicola	0.9	0.31	0.47	1.77	0.56	50.92
Bacteria;__Candidate_division_OD1;__c;__o;__f;__g	0.3	0.56	0.47	1.09	0.55	51.47
Bacteria;__Proteobacteria;__Alphaproteobacteria;__SAR11_clade;Other;Other	0.05	0.61	0.47	0.78	0.55	52.02
Archaea;__Euryarchaeota;__Methanomicrobia;__Methanomicrobiales;__Methanoregulaceae;__Methanoregula	0.61	0	0.46	10.09	0.54	52.57
Bacteria;__Actinobacteria;__Acidimicrobiia;__Acidimicrobiales;__Acidimicrobiaceae;__g	0.13	0.54	0.43	0.8	0.51	53.07
Bacteria;__Proteobacteria;__Betaproteobacteria;__Burkholderiales;__Alcaligenaceae;Other	0.64	0.07	0.42	2.22	0.5	53.57
Eukaryota;__Fungi;__Chytridiomycota;__;__;__	0.63	0.11	0.42	1.2	0.5	54.07
Bacteria;__Proteobacteria;__Deltaproteobacteria;__Bdellovibrionales;__Bacteriovoracaceae;__Peredibacter	0.04	0.59	0.41	1.51	0.48	54.55
Eukaryota;__Alveolata;__Ciliophora;__Conthreep;__Phyllopharyngea;__Dysteria	0	0.59	0.41	0.78	0.48	55.03
Bacteria;__Proteobacteria;__Betaproteobacteria;__Burkholderiales;Other;Other	0.53	0	0.39	2.62	0.46	55.49
Unassigned;Other;Other;Other;Other;Other	0.91	1.43	0.39	1.43	0.46	55.95
Bacteria;__Proteobacteria;__Deltaproteobacteria;__Bdellovibrionales;__Bacteriovoracaceae;__Bacteriovorax	0.5	0	0.38	4.85	0.45	56.4
Bacteria;__Actinobacteria;__Acidimicrobiia;__Acidimicrobiales;__Acidimicrobiaceae;__CL500-29_marine_group	0.49	0	0.38	0.97	0.45	56.85
Bacteria;__Proteobacteria;__Alphaproteobacteria;__Rhodobacterales;__Rhodobacteraceae;__Roseobacter_clade_DC5-80-3_lineage	0.07	0.55	0.38	2.35	0.44	57.29
Bacteria;__Bacteroidetes;__Sphingobacteriia;__Sphingobacteriales;__Saprospiraceae;__Lewinella	0	0.48	0.34	1.08	0.4	57.69
Bacteria;__Bacteroidetes;__Cytophagia;__Cytophagales;__Cytophagaceae;__Leadbetterella	0.5	0.18	0.34	1.19	0.4	58.08
Bacteria;__Candidate_division_TM7;__c;__o;__f;__g	0.41	0.19	0.34	1.07	0.4	58.48
Bacteria;__Proteobacteria;__Betaproteobacteria;Other;Other;Other	0.54	0.68	0.33	1.2	0.39	58.87
Bacteria;__Bacteroidetes;__Sphingobacteriia;__Sphingobacteriales;__WCHB1-69;__g	0.44	0	0.33	1.13	0.38	59.26
Bacteria;__Bacteroidetes;__Sphingobacteriia;__Sphingobacteriales;__Chitinophagaceae;__Filimonas	0.42	0	0.31	0.94	0.37	59.62
Bacteria;__Proteobacteria;__Gammaproteobacteria;__Pseudomonadales;__Moraxellaceae;__Psychrobacter	0.53	0.16	0.31	1.53	0.36	59.98
Bacteria;__Proteobacteria;__Gammaproteobacteria;__Oceanospirillales;__oc58;__g	0.39	0	0.29	2.12	0.35	60.33

Bacteria;__WCHB1-60;__c;__o;__f;__g	0.37	0	0.29	0.79	0.34	60.67
Bacteria;__Bacteroidetes;__Flavobacteria;__Flavobacteriales;__Cryomorphaceae;__Owenweeksia	0	0.39	0.29	2.35	0.34	61.01
Bacteria;__Proteobacteria;__Alphaproteobacteria;__SAR11_clade;__Surface_4;__g	0	0.36	0.28	0.67	0.33	61.34
Bacteria;__Verrucomicrobia;__Opitutae;__Opitutales;__Opitutaceae;__Opitutus	0.36	0	0.27	1.48	0.32	61.65
Bacteria;__Proteobacteria;__Alphaproteobacteria;__Rhodobacterales;__Rhodobacteraceae;__Loktanella	0	0.36	0.26	1.31	0.31	61.96
Bacteria;__Actinobacteria;__Acidimicrobiia;__Acidimicrobiales;__uncultured;__g	0.41	0.06	0.26	2.01	0.31	62.27
Bacteria;__Gemmatimonadetes;__Gemmatimonadetes;__Gemmatimonadales;__Gemmatimonadaceae;__Gemmatimonas	0.35	0	0.26	1.7	0.31	62.58
Bacteria;__Proteobacteria;__Gammaproteobacteria;__Alteromonadales;__Alteromonadaceae;__Glaciecola	0	0.36	0.25	1.02	0.3	62.87
Bacteria;__Bacteroidetes;__vadinHA17;__o;__f;__g	0.34	0	0.25	2.49	0.3	63.17
Bacteria;__Bacteroidetes;__Flavobacteria;__Flavobacteriales;__Flavobacteriaceae;__NS2b_marine_group	0.09	0.42	0.25	3.85	0.29	63.47
Archaea;__Euryarchaeota;__Thermoplasmata;__Thermoplasmatales;__Marine_Group_II;__g	0.05	0.37	0.25	2.15	0.29	63.76
Bacteria;__Bacteroidetes;__Flavobacteria;__Flavobacteriales;__Flavobacteriaceae;__NS5_marine_group	0	0.32	0.25	1.35	0.29	64.05
Bacteria;__Proteobacteria;__Betaproteobacteria;__Burkholderiales;__Comamonadaceae;__Acidovorax	0.38	0.14	0.25	1.33	0.29	64.34
Bacteria;__Bacteroidetes;__Flavobacteria;__Flavobacteriales;__Flavobacteriaceae;__NS4_marine_group	0.05	0.36	0.24	2.35	0.28	64.62
Bacteria;__Proteobacteria;__Gammaproteobacteria;__Oceanospirillales;__Oceanospirillaceae;Other	0.05	0.36	0.24	2.14	0.28	64.9
Bacteria;__Proteobacteria;__Gammaproteobacteria;__Alteromonadales;__Alteromonadaceae;__SAR92_clade	0.07	0.35	0.22	1.88	0.26	65.16
Bacteria;__Bacteroidetes;__Sphingobacteriia;__Sphingobacteriales;__Saprospiraceae;__Aureispira	0	0.32	0.22	0.82	0.26	65.42
Eukaryota;__Alveolata;__Ciliophora;__Spirotrichea;__Hypotrichia;__	0	0.31	0.22	1.58	0.26	65.68
Bacteria;__Proteobacteria;__Gammaproteobacteria;__BD7-8_marine_group;__f;__g	0	0.29	0.22	1.19	0.26	65.94
Eukaryota;__Chloroplastida;__Ulvophyceae;Other;Other;Other	0	0.31	0.22	1.12	0.25	66.19
Bacteria;__Bacteroidetes;__Cytophagia;__Cytophagales;__Flammeovirgaceae;__Reichenbachiella	0	0.29	0.21	2.3	0.25	66.44
Bacteria;__Proteobacteria;__Gammaproteobacteria;__Alteromonadales;__Alteromonadaceae;__BD1-7_clade	0.29	0	0.21	1.51	0.25	66.69
Bacteria;__Proteobacteria;__Betaproteobacteria;__Burkholderiales;__Comamonadaceae;__Rhizobacter	0.28	0	0.21	2.4	0.25	66.94
Bacteria;__Fusobacteria;__Fusobacteria;__Fusobacteriales;__Fusobacteriaceae;__Fusobacterium	0.38	0.36	0.21	1.29	0.25	67.19
Bacteria;__Proteobacteria;__Betaproteobacteria;__Burkholderiales;__Oxalobacteraceae;__Undibacterium	0.28	0	0.21	3.2	0.25	67.43
Bacteria;__Proteobacteria;__Gammaproteobacteria;__Alteromonadales;__Shewanellaceae;__Shewanella	0	0.28	0.21	3.04	0.25	67.68
Bacteria;__Bacteroidetes;__Bacteroidia;__Bacteroidales;__Bacteroidaceae;__Bacteroides	0.21	0.3	0.21	1.33	0.25	67.93
Bacteria;__Proteobacteria;__Alphaproteobacteria;__Rhodobacterales;__Rhodobacteraceae;__Rhodobacter	0.32	0.11	0.21	1.49	0.24	68.17
Bacteria;__Proteobacteria;__Alphaproteobacteria;__Kordiimonadales;__Kordiimonadaceae;__Kordiimonas	0	0.28	0.21	1.74	0.24	68.41
Eukaryota;__Alveolata;__Ciliophora;__Spirotrichea;__Oligotrichia;__Strombidium	0	0.27	0.2	2.34	0.24	68.65

Bacteria;__Proteobacteria;__Betaproteobacteria;__Neisseriales;__Neisseriaceae;__g	0.27	0	0.2	2.09	0.23	68.88
Bacteria;__Proteobacteria;__Deltaproteobacteria;__Myxococcales;__0319-6G20;__g	0.36	0.15	0.2	1.19	0.23	69.12
Eukaryota;__Fungi;__LKM11;__;__;__	0.26	0	0.2	2.62	0.23	69.35
Bacteria;__Proteobacteria;__Gammaproteobacteria;__Enterobacteriales;__Enterobacteriaceae;Other	0.13	0.37	0.2	1.03	0.23	69.58
Bacteria;__Proteobacteria;__Alphaproteobacteria;__Sphingomonadales;__Sphingomonadaceae;__Sandarakinorhabdus	0.27	0.04	0.19	1.41	0.23	69.8
Eukaryota;__Alveolata;__Ciliophora;__Spirotrichea;__Oligotrichia;__uncultured	0	0.26	0.19	3.2	0.22	70.03
Bacteria;__Proteobacteria;__Alphaproteobacteria;__Rickettsiales;__SAR116_clade;__g	0	0.24	0.19	1.23	0.22	70.25
Bacteria;__Proteobacteria;__Alphaproteobacteria;__SAR11_clade;__f;__g	0	0.24	0.19	0.86	0.22	70.47
Bacteria;__Bacteroidetes;__Flavobacteria;__Flavobacteriales;__Flavobacteriaceae;__Pibocella	0	0.26	0.19	0.96	0.22	70.68
Bacteria;__Proteobacteria;__Gammaproteobacteria;__Alteromonadales;Other;Other	0	0.24	0.18	1.93	0.22	70.9
Eukaryota;__Alveolata;__Ciliophora;__Spirotrichea;__Euplotia;__Aspidisca	0	0.26	0.18	1.81	0.22	71.12
Bacteria;__Bacteroidetes;__Sphingobacteriia;__Sphingobacteriales;__env.OPS_17;__g	0.25	0	0.18	1.83	0.22	71.33
Eukaryota;__Stramenopiles;__T53A1;__;__;__	0.24	0	0.18	1.79	0.22	71.55
Bacteria;__Proteobacteria;__Gammaproteobacteria;__Alteromonadales;__Moritellaceae;__Moritella	0	0.26	0.18	1.13	0.21	71.76
Bacteria;__Bacteroidetes;__Flavobacteria;__Flavobacteriales;__NS9_marine_group;__g	0.21	0.4	0.18	1.12	0.21	71.98
Eukaryota;__Metazoa;__Arthropoda;__Maxillopoda;__;__	0	0.25	0.18	2.15	0.21	72.19
Bacteria;__Proteobacteria;__Gammaproteobacteria;__Thiotrichales;__Thiotrichaceae;__Thiothrix	0	0.23	0.18	0.67	0.21	72.4
Bacteria;__Candidate_division_OP3;__c;__o;__f;__g	0.11	0.23	0.18	1.15	0.21	72.6
Eukaryota;__Alveolata;__Protalveolata;__Perkinsidae;__NA2-2D10;__	0	0.23	0.17	1.18	0.2	72.81
Bacteria;__Proteobacteria;__Gammaproteobacteria;__Chromatiales;__Granulosicoccaceae;__Granulosicoccus	0	0.24	0.17	1.33	0.2	73.01
Bacteria;__Firmicutes;__Clostridia;__Clostridiales;__Ruminococcaceae;__g	0.24	0.21	0.17	1.5	0.2	73.22
Bacteria;__Firmicutes;__Bacilli;__Bacillales;__Staphylococcaceae;Other	0.23	0	0.17	1.33	0.2	73.42
Eukaryota;__Fungi;Other;Other;Other;Other	0.23	0	0.17	1.79	0.2	73.62
Bacteria;__Proteobacteria;__Betaproteobacteria;__Burkholderiales;__Comamonadaceae;__Comamonas	0.22	0	0.17	1.09	0.2	73.81
Bacteria;__Planctomycetes;__BD7-11;__o;__f;__g	0	0.21	0.16	2.08	0.19	74.01
Bacteria;__Bacteroidetes;__Bacteroidia;__Bacteroidales;__Prevotellaceae;__Prevotella	0.24	0.21	0.16	1.46	0.19	74.2
Eukaryota;__Stramenopiles;__Chrysophyceae;Other;Other;Other	0.22	0	0.16	3.32	0.19	74.39
Bacteria;__Proteobacteria;__Gammaproteobacteria;__Alteromonadales;__Alteromonadaceae;Other	0.02	0.25	0.16	3.02	0.19	74.58
Bacteria;__Proteobacteria;__Gammaproteobacteria;__Oceanospirillales;__Oceanospirillaceae;__Neptunomonas	0	0.23	0.16	0.89	0.19	74.77
Bacteria;__Proteobacteria;__Alphaproteobacteria;__Sphingomonadales;__Erythrobacteraceae;__Altererythrobacter	0	0.2	0.16	0.67	0.19	74.96
Bacteria;__Proteobacteria;__Gammaproteobacteria;__Thiotrichales;__Thiotrichaceae;__Leucothrix	0	0.23	0.16	0.9	0.19	75.15

Bacteria;__Proteobacteria;__Alphaproteobacteria;__Rhodospirillales;__Rhodospirillaceae;__Defluviicoccus	0	0.2	0.16	0.67	0.19	75.33
Eukaryota;__Chloroplastida;__Chlorophyceae;__Chlamydomonas;__;__	0.21	0	0.16	1.02	0.19	75.52
Bacteria;__Proteobacteria;__Alphaproteobacteria;Other;Other;Other	0.02	0.21	0.16	0.89	0.18	75.7
Bacteria;__Proteobacteria;__Betaproteobacteria;__Nitrosomonadales;__Nitrosomonadaceae;__Nitrosomonas	0.21	0.06	0.15	1.04	0.18	75.88
Bacteria;__Proteobacteria;__Gammaproteobacteria;__Order_Incertae_Sedis;__Family_Incertae_Sedis;__Arenicella	0	0.21	0.15	1.15	0.18	76.06
Eukaryota;__Alveolata;__Ciliophora;__Spirotrichea;__Hypotrichia;Other	0.14	0.3	0.15	1.54	0.18	76.24
Bacteria;__Bacteroidetes;__Bacteroidia;__Bacteroidales;__Marinilabiaceae;__Marinifilum	0	0.22	0.15	0.91	0.18	76.42
Bacteria;__Proteobacteria;__Gammaproteobacteria;Other;Other;Other	0.2	0.38	0.15	1.57	0.17	76.59
Bacteria;__Actinobacteria;__Actinobacteria;Other;Other;Other	0.19	0	0.15	1.23	0.17	76.77
Bacteria;__Proteobacteria;__Alphaproteobacteria;__Rhodobacterales;__Rhodobacteraceae;__Sulfitobacter	0	0.22	0.15	0.67	0.17	76.94
Bacteria;__Proteobacteria;__Alphaproteobacteria;__Rhodobacterales;__Rhodobacteraceae;__Paracoccus	0.18	0.07	0.15	0.86	0.17	77.11
Eukaryota;__Rhizaria;__Cercozoa;Other;Other;Other	0.02	0.21	0.14	1.11	0.17	77.28
Bacteria;Other;Other;Other;Other;Other	0.19	0	0.14	1.64	0.16	77.44
Bacteria;__Proteobacteria;__Alphaproteobacteria;__Rhodospirillales;__Rhodospirillaceae;__Thalassospira	0	0.17	0.14	1.29	0.16	77.6
Eukaryota;__Stramenopiles;__Diatomea;__Coscinodiscophytina;__Fragilariales;__	0	0.19	0.13	0.95	0.16	77.76
Bacteria;__Actinobacteria;__Actinobacteria;__Corynebacteriales;__Mycobacteriaceae;__Mycobacterium	0.18	0	0.13	1.08	0.16	77.92
Bacteria;__Proteobacteria;Other;Other;Other;Other	0.24	0.06	0.13	1.47	0.16	78.08
Bacteria;__Proteobacteria;__Gammaproteobacteria;__Pseudomonadales;__Pseudomonadaceae;__Pseudomonas	0.12	0.3	0.13	1.23	0.15	78.23
Bacteria;__Proteobacteria;__Gammaproteobacteria;__Oceanospirillales;__Oceanospirillaceae;__Marinomonas	0	0.17	0.13	1.08	0.15	78.38
Bacteria;__Verrucomicrobia;__Verrucomicrobiae;__Verrucomicrobiales;__Verrucomicrobiaceae;__g	0.17	0	0.13	3.01	0.15	78.54
Bacteria;__Bacteroidetes;__Sphingobacteriia;__Sphingobacteriales;Other;Other	0.17	0	0.13	3.69	0.15	78.69
Bacteria;__Proteobacteria;__Deltaproteobacteria;__Syntrophobacterales;__Syntrophaceae;__Smithella	0.16	0	0.13	0.66	0.15	78.84
Bacteria;__Proteobacteria;__Alphaproteobacteria;__Rhizobiales;__Hyphomicrobiaceae;__Devosia	0.17	0	0.13	1.32	0.15	78.99
Bacteria;__Lentisphaerae;__Lentisphaeria;__Lentisphaerales;__Lentisphaeraceae;__Lentisphaera	0	0.18	0.13	0.97	0.15	79.14
Bacteria;__Actinobacteria;__Acidimicrobiia;__Acidimicrobiales;__OCS155_marine_group;__g	0	0.16	0.13	0.67	0.15	79.29
Bacteria;__Proteobacteria;__Gammaproteobacteria;__Oceanospirillales;__Oceanospirillaceae;__Pseudospirillum	0.18	0.06	0.13	1.25	0.15	79.43
Bacteria;__Bacteroidetes;__WCHB1-32;__o;__f;__g	0.17	0	0.13	1.22	0.15	79.58
Eukaryota;__Alveolata;__Ciliophora;__Conthreep;__Phyllopharyngea;__Hartmannula	0	0.18	0.12	0.67	0.15	79.73
Bacteria;__Proteobacteria;__Deltaproteobacteria;__Desulfobacterales;__Nitrospinaceae;__Nitrospina	0	0.16	0.12	1.16	0.14	79.87

Bacteria;__Proteobacteria;__Betaproteobacteria;__Rhodocyclales;__Rhodocyclaceae;Other	0.16	0	0.12	3.16	0.14	80.01

Groups Atlas Cove & Winston Lagoon

Average dissimilarity = 68.61

Species	Atlas Cove Av.Abund	Winston Lagoon Av.Abund	Av.Diss	Diss/SD	Contrib%	Cum.%
Bacteria;__Proteobacteria;__Gammaproteobacteria;Other;Other;Other	3.15	0.38	2.16	2.49	3.14	3.14
Bacteria;__Proteobacteria;__Alphaproteobacteria;__SAR11_clade;__Surface_1;__g	2.82	2.12	2.1	1.36	3.06	6.2
Bacteria;__Cyanobacteria;__Chloroplast;__Chloroplast;__Chloroplast;__Chloroplast	2.38	3.37	1.93	1.14	2.81	9.01
Bacteria;__Proteobacteria;__Gammaproteobacteria;__Oceanospirillales;__ZD0405;__g	3.19	1.34	1.72	1.34	2.51	11.53
Bacteria;__Proteobacteria;__Gammaproteobacteria;__Alteromonadales;__Alteromonadaceae;__SAR92_clade	2.09	0.35	1.34	2.01	1.96	13.48
Bacteria;__Bacteroidetes;__Flavobacteria;__Flavobacteriales;__Flavobacteriaceae;Other	1.01	2.34	1.33	0.91	1.94	15.42
Bacteria;__Proteobacteria;__Alphaproteobacteria;__Sphingomonadales;__Sphingomonadaceae;__Sphingopyxis	0.26	1.75	1.31	0.71	1.91	17.33
Bacteria;__Proteobacteria;__Alphaproteobacteria;__Rhodobacterales;__Rhodobacteraceae;Other	0.29	1.87	1.24	1.41	1.81	19.14
Bacteria;__Bacteroidetes;__Flavobacteria;__Flavobacteriales;__Flavobacteriaceae;__NS2b_marine_group	1.92	0.42	1.19	3.32	1.73	20.87
Bacteria;__Proteobacteria;__Gammaproteobacteria;__Oceanospirillales;__SAR86_clade;__g	1.99	1.39	1.08	1.93	1.58	22.45
Archaea;__Thaumarchaeota;__Marine_Group_I;__o;__f;__g	0.65	1.55	0.97	0.85	1.42	23.86
Bacteria;__Bacteroidetes;__Flavobacteria;__Flavobacteriales;__NS9_marine_group;__g	1.54	0.4	0.9	3.14	1.31	25.17
Archaea;__Euryarchaeota;__Halobacteria;__Halobacteriales;__Deep_Sea_Hydrothermal_Vent_Gp_6(DHVEG-6);__Candidatus_Parvarchaeum	0	1.11	0.89	0.84	1.3	26.47
Bacteria;__Proteobacteria;__Betaproteobacteria;__Burkholderiales;__Comamonadaceae;Other	0.03	1.03	0.86	0.69	1.25	27.72
Bacteria;__Proteobacteria;__Gammaproteobacteria;__Alteromonadales;__Pseudoalteromonadaceae;__Pseudoalteromonas	1.18	0	0.86	1.54	1.25	28.97
Bacteria;__Proteobacteria;__Alphaproteobacteria;__Rhodobacterales;__Rhodobacteraceae;__Roseobacter_clade_DC5-80-3_lineage	1.59	0.55	0.83	2.06	1.21	30.17
Bacteria;__Proteobacteria;__Gammaproteobacteria;__Alteromonadales;__Pseudoalteromonadaceae;__g	0.07	1.15	0.81	1.51	1.18	31.35
Bacteria;__Bacteroidetes;__Flavobacteria;__Flavobacteriales;__Flavobacteriaceae;__Polaribacter	1.7	0.91	0.81	1.66	1.18	32.53
Bacteria;__Proteobacteria;__Gammaproteobacteria;__Alteromonadales;__Alteromonadaceae;__Alteromonas	1.1	0	0.8	1.32	1.16	33.69
Bacteria;__Bacteroidetes;__Flavobacteria;__Flavobacteriales;__Flavobacteriaceae;__NS4_marine_group	1.31	0.36	0.79	1.46	1.15	34.84
Archaea;__Euryarchaeota;__Thermoplasmata;__Thermoplasmatales;__Marine_Group_II;__g	1.28	0.37	0.75	1.46	1.09	35.93

Eukaryota;__Stramenopiles;__MAST-1;__MAST-1 B;__;__	1	0	0.73	1.29	1.06	36.99
Bacteria;__Actinobacteria;__Actinobacteria;__Corynebacteriales;__Nocardiaceae;__Rhodococcus	0	0.96	0.7	1.1	1.02	38.02
Eukaryota;Other;Other;Other;Other;Other	0.97	0.11	0.64	0.86	0.94	38.95
Eukaryota;__Haptophyta;__Prymnesiophyceae;Other;Other;Other	0.86	0	0.62	1.32	0.91	39.86
Bacteria;__Proteobacteria;__Alphaproteobacteria;__SAR11_clade;Other;Other	0.9	0.61	0.62	1.58	0.9	40.76
Eukaryota;__Chloroplastida;__Mamiellophyceae;__Micromonas;__;__	0.33	0.8	0.57	0.92	0.83	41.59
Bacteria;__Bacteroidetes;__Flavobacteria;__Flavobacteriales;__Flavobacteriaceae;__NS3a_marine_group	0.38	0.9	0.57	0.79	0.83	42.43
Bacteria;__Bacteroidetes;__Flavobacteria;__Flavobacteriales;Other;Other	0.84	0.09	0.56	1.86	0.82	43.25
Archaea;__Euryarchaeota;__Halobacteria;__Halobacteriales;__Deep_Sea_Hydrothermal_Vent_Gp_6(DHVEG-6);__g	0	0.68	0.54	0.78	0.78	44.03
Eukaryota;__Alveolata;__Ciliophora;__Conthreep;__Phyllopharyngea;__AT1-2	0	0.75	0.54	0.9	0.78	44.81
Eukaryota;__Metazoa;__Arthropoda;__Maxillopoda;__;__	0.78	0.25	0.52	0.91	0.76	45.57
Eukaryota;__Alveolata;__Dinoflagellata;__Dinophyceae;__Gymnodiniphycidae;__Gyrodinium	0.77	0.09	0.52	0.76	0.75	46.32
Bacteria;__Proteobacteria;__Gammaproteobacteria;__Alteromonadales;__Colwelliaceae;__Colwellia	0.61	0.95	0.5	1.39	0.73	47.05
Bacteria;__Proteobacteria;__Gammaproteobacteria;__Oceanospirillales;__Alcanivoracaceae;__Alcanivorax	0.67	0	0.49	2.4	0.72	47.77
Bacteria;__Proteobacteria;__Alphaproteobacteria;__Rhodobacterales;__Rhodobacteraceae;__Sulfitobacter	0.73	0.22	0.49	1.34	0.71	48.48
Eukaryota;__Picozoa;__;__;__;__	0.68	0	0.49	1.28	0.71	49.19
Bacteria;__Proteobacteria;__Alphaproteobacteria;__Rhodobacterales;__Rhodobacteraceae;__g	0.85	0.89	0.48	1.58	0.69	49.89
Bacteria;__Proteobacteria;__Deltaproteobacteria;__Bdellovibrionales;__Bacteriovoracaceae;__Peredibacter	0	0.59	0.46	1.62	0.67	50.55
Bacteria;__Candidate_division_OD1;__c;__o;__f;__g	0	0.56	0.45	0.82	0.66	51.21
Unassigned;Other;Other;Other;Other;Other	1.01	1.43	0.45	1.64	0.66	51.87
Bacteria;__Actinobacteria;__Acidimicrobiia;__Acidimicrobiales;__Acidimicrobiaceae;__g	0.04	0.54	0.45	0.73	0.65	52.52
Eukaryota;__Stramenopiles;__MAST-3;__;__;__	0.61	0	0.44	1.29	0.65	53.17
Bacteria;__Bacteroidetes;__Flavobacteria;__Flavobacteriales;__Flavobacteriaceae;__g	0.39	0.68	0.44	1.11	0.64	53.81
Eukaryota;__Stramenopiles;__MAST-7;__;__;__	0.62	0.09	0.44	1.44	0.64	54.45
Eukaryota;__Alveolata;__Ciliophora;__Conthreep;__Phyllopharyngea;__Dysteria	0	0.59	0.42	0.78	0.61	55.05
Bacteria;__Proteobacteria;__Alphaproteobacteria;__SAR11_clade;__Surface_4;__g	0.49	0.36	0.41	1.87	0.6	55.66
Bacteria;__Deferribacteres;__Deferribacteres;__Deferribacterales;__SAR406_clade(Marine_group_A);__g	0.55	0.12	0.4	1.07	0.58	56.24
Bacteria;__Proteobacteria;__Betaproteobacteria;Other;Other;Other	0.45	0.68	0.37	1.21	0.54	56.78
Bacteria;__Proteobacteria;__Gammaproteobacteria;__Pseudomonadales;__Moraxellaceae;__Perlucidibaca	0.49	0	0.35	1.34	0.51	57.29
Bacteria;__Bacteroidetes;__Sphingobacteriia;__Sphingobacteriales;__Saprospiraceae;__Lewinella	0	0.48	0.35	1.07	0.51	57.8
Eukaryota;__Alveolata;__Protalveolata;__Syndiniales;__Syndiniales Group I;__	0.48	0	0.35	1.3	0.51	58.31

Bacteria;__Proteobacteria;__Gammaproteobacteria;__Oceanospirillales;__OM182_clade;__g	0.55	0.09	0.34	1.63	0.5	58.81
Bacteria;__Bacteroidetes;__Flavobacteria;__Flavobacteriales;__Flavobacteriaceae;__NS5_marine_group	0.6	0.32	0.34	1.08	0.5	59.3
Eukaryota;__Alveolata;__Ciliophora;__Spirotrichea;__Oligotrichia;__Strombidium	0.47	0.27	0.33	1.24	0.48	59.79
Eukaryota;__Alveolata;__Ciliophora;__Spirotrichea;__Oligotrichia;__uncultured	0.66	0.26	0.33	0.83	0.48	60.27
Eukaryota;__Holozoa;__Choanomonada;__Acanthoecida;__Diaphanoeca;__	0.45	0.06	0.31	0.95	0.45	60.72
Eukaryota;__Alveolata;__Protalveolata;__Syndiniales;__Syndiniales Group II;__	0.41	0.06	0.29	1.37	0.42	61.14
Bacteria;__Proteobacteria;__Alphaproteobacteria;__Rickettsiales;__SAR116_clade;__g	0.57	0.24	0.28	1.66	0.41	61.55
Eukaryota;__Stramenopiles;__MAST-1;__MAST-1 A;__;__	0.4	0.06	0.28	1.41	0.4	61.95
Bacteria;__Proteobacteria;__Alphaproteobacteria;__Rhodobacterales;__Rhodobacteraceae;__Loktanella	0	0.36	0.27	1.29	0.4	62.35
Bacteria;__Fusobacteria;__Fusobacteria;__Fusobacteriales;__Fusobacteriaceae;__Fusobacterium	0	0.36	0.27	1.65	0.39	62.74
Bacteria;__Proteobacteria;__Gammaproteobacteria;__Alteromonadales;__Alteromonadaceae;__Glaciecola	0	0.36	0.26	1.01	0.38	63.12
Eukaryota;__Alveolata;__Dinoflagellata;__Dinophyceae;__Gymnodiniphycidae;Other	0.38	0	0.26	0.76	0.38	63.5
Bacteria;__Proteobacteria;__Gammaproteobacteria;__Enterobacteriales;__Enterobacteriaceae;Other	0.05	0.37	0.26	1.23	0.38	63.87
Bacteria;__Bacteroidetes;__Sphingobacteriia;__Sphingobacteriales;__NS11-12_marine_group;__g	0	0.35	0.26	0.98	0.37	64.24
Eukaryota;__Metazoa;__Cnidaria;__Hydrozoa;__;__	0.33	0.09	0.25	0.91	0.37	64.61
Eukaryota;__Alveolata;__Ciliophora;__Spirotrichea;__Choreotrichia;__uncultured	0.37	0.06	0.25	0.87	0.36	64.98
Bacteria;__Proteobacteria;__Gammaproteobacteria;__Oceanospirillales;__Oceanospirillaceae;Other	0.68	0.36	0.25	1.63	0.36	65.34
Bacteria;__Bacteroidetes;__Cytophagia;__Cytophagales;__Flammeovirgaceae;__Marinoscillum	0.33	0.16	0.25	1	0.36	65.7
Bacteria;__Proteobacteria;__Alphaproteobacteria;Other;Other;Other	0.42	0.21	0.24	1.46	0.35	66.06
Bacteria;__Proteobacteria;__Deltaproteobacteria;__SAR324_clade(Marine_group_B);__f;__g	0.35	0.09	0.24	1.14	0.35	66.41
Eukaryota;__Rhizaria;__Cercozoa;__Thecofilosea;__Cryomonadida;__Cryothecomonas	0.34	0.11	0.24	1.03	0.35	66.76
Bacteria;__Bacteroidetes;__Sphingobacteriia;__Sphingobacteriales;__Saprospiraceae;__g	0	0.34	0.23	0.66	0.34	67.1
Bacteria;__Bacteroidetes;__Sphingobacteriia;__Sphingobacteriales;__Saprospiraceae;__Aureispira	0	0.32	0.23	0.82	0.33	67.43
Eukaryota;__Alveolata;__Ciliophora;__Spirotrichea;__Hypotrichia;__	0	0.31	0.23	1.56	0.33	67.76
Eukaryota;__Alveolata;__Ciliophora;__Spirotrichea;__Hypotrichia;Other	0	0.3	0.23	2.05	0.33	68.09
Bacteria;__Proteobacteria;__Alphaproteobacteria;__Rhodospirillales;__Rhodospirillaceae;__Defluviicoccus	0.25	0.2	0.22	2.9	0.33	68.42
Eukaryota;__Chloroplastida;__Ulvophyceae;Other;Other;Other	0	0.31	0.22	1.11	0.32	68.74
Bacteria;__Proteobacteria;__Alphaproteobacteria;__SAR11_clade;__f;__g	0.32	0.24	0.22	1.11	0.32	69.07
Bacteria;__Proteobacteria;__Gammaproteobacteria;__Alteromonadales;__Shewanellaceae;__Shewanella	0	0.28	0.22	2.87	0.32	69.38
Eukaryota;__Stramenopiles;__MAST-8;__;__;__	0.29	0	0.21	1.22	0.31	69.7
Bacteria;__Bacteroidetes;__Cytophagia;__Cytophagales;__Flammeovirgaceae;Other	0.27	0	0.21	1.89	0.31	70.01
Bacteria;__Bacteroidetes;__Bacteroidia;__Bacteroidales;__Bacteroidaceae;__Bacteroides	0.04	0.3	0.21	1.35	0.31	70.32
Bacteria;__Proteobacteria;__Gammaproteobacteria;__Thiotrichales;__Thiotrichaceae;__Thiothrix	0.12	0.23	0.21	1.12	0.31	70.63

Bacteria;__Proteobacteria;__Alphaproteobacteria;__Kordiimonadales;__Kordiimonadaceae;__Kordiimonas	0	0.28	0.21	1.71	0.31	70.94
Eukaryota;__Alveolata;__Dinoflagellata;__Dinophyceae;__Gymnodiniphycidae;__Gymnodinium	0.39	0.13	0.21	0.76	0.3	71.25
Bacteria;__Proteobacteria;__Gammaproteobacteria;__BD7-8_marine_group;__f;__g	0.08	0.29	0.21	1.34	0.3	71.55
Eukaryota;__Haptophyta;__Prymnesiophyceae;__Prymnesiales;Other;Other	0.27	0	0.2	1.32	0.29	71.83
Eukaryota;__Rhizaria;__Cercozoa;__Thecofilosea;Other;Other	0.19	0.2	0.19	1.28	0.28	72.12
Bacteria;__Proteobacteria;__Gammaproteobacteria;__Alteromonadales;Other;Other	0	0.24	0.19	1.86	0.28	72.4
Eukaryota;__Alveolata;__Ciliophora;__Spirotrichea;__Euplotia;__Aspidisca	0	0.26	0.19	1.78	0.28	72.67
Bacteria;Other;Other;Other;Other;Other	0.25	0	0.19	3.8	0.27	72.95
Eukaryota;__Metazoa;__Annelida;__;__;__	0.27	0.09	0.18	1.11	0.27	73.22
Bacteria;__Verrucomicrobia;__Opitutae;__MB11C04_marine_group;__f;__g	0.24	0	0.18	2.39	0.27	73.48
Bacteria;__Bacteroidetes;__Flavobacteria;__Flavobacteriales;__Flavobacteriaceae;__Pibocella	0.03	0.26	0.18	0.99	0.26	73.75
Bacteria;__Candidate_division_OP3;__c;__o;__f;__g	0	0.23	0.18	0.94	0.26	74.01
Eukaryota;__Alveolata;__Protalveolata;__Perkinsidae;__NA2-2D10;__	0	0.23	0.18	1.16	0.26	74.27
Bacteria;__Proteobacteria;__Gammaproteobacteria;__Chromatiales;__Granulosicoccaceae;__Granulosicoccus	0	0.24	0.18	1.31	0.26	74.53
Eukaryota;__Telonema;__Incertae Sedis;__;__;__	0.23	0.07	0.17	0.86	0.25	74.78
Bacteria;__Proteobacteria;__Gammaproteobacteria;__Pseudomonadales;__Pseudomonadaceae;__Pseudomonas	0.08	0.3	0.17	1.59	0.25	75.04
Bacteria;__Planctomycetes;__BD7-11;__o;__f;__g	0	0.21	0.17	2.01	0.25	75.29
Bacteria;__Proteobacteria;__Gammaproteobacteria;__Alteromonadales;__Alteromonadaceae;Other	0.45	0.25	0.17	1.59	0.25	75.53
Bacteria;__Proteobacteria;__Gammaproteobacteria;__Oceanospirillales;__Oceanospirillaceae;__Balneatrix	0.25	0.04	0.17	1.32	0.24	75.77
Eukaryota;__Stramenopiles;__MAST-1;__MAST-1 C;__;__	0.24	0	0.17	0.92	0.24	76.02
Bacteria;__Proteobacteria;__Alphaproteobacteria;__Sphingomonadales;__Erythrobacteraceae;__Altererythrobacter	0	0.2	0.17	0.66	0.24	76.26
Bacteria;__Proteobacteria;__Gammaproteobacteria;__Alteromonadales;__Moritellaceae;__Moritella	0.18	0.26	0.17	1.26	0.24	76.5
Bacteria;__Proteobacteria;__Gammaproteobacteria;__Thiotrichales;__Thiotrichaceae;__Leucothrix	0	0.23	0.16	0.89	0.24	76.74
Bacteria;__Bacteroidetes;__Flavobacteria;__Flavobacteriales;__Cryomorphaceae;__Owenweeksia	0.47	0.39	0.16	1.11	0.23	76.97
Eukaryota;__Alveolata;__Ciliophora;__Spirotrichea;__Choreotrichia;Other	0.22	0	0.16	0.94	0.23	77.2
Eukaryota;__Chloroplastida;__Prasinophytae;__Pyramimonas;__;__	0.22	0.06	0.16	0.96	0.23	77.43
Bacteria;__Candidate_division_TM7;__c;__o;__f;__g	0	0.19	0.16	0.66	0.23	77.66
Bacteria;__Bacteroidetes;__Cytophagia;__Cytophagales;__Cytophagaceae;__Leadbetterella	0	0.18	0.15	0.66	0.23	77.88
Bacteria;__Proteobacteria;Other;Other;Other;Other	0.25	0.06	0.15	1.72	0.22	78.1
Bacteria;__Bacteroidetes;__Bacteroidia;__Bacteroidales;__Prevotellaceae;__Prevotella	0	0.21	0.15	1.6	0.22	78.33
Bacteria;__Candidate_division_OP11;__c;__o;__f;__g	0	0.19	0.15	0.66	0.22	78.55

Bacteria;__Proteobacteria;__Gammaproteobacteria;__Oceanospirillales;Other;Other	0.3	0.11	0.15	1.4	0.22	78.77
Bacteria;__Proteobacteria;__Gammaproteobacteria;__Oceanospirillales;__Oceanospirillaceae;__Neptunomonas	0.07	0.23	0.15	0.89	0.22	78.99
Bacteria;__Firmicutes;__Clostridia;__Clostridiales;__Ruminococcaceae;__g	0	0.21	0.15	0.92	0.22	79.21
Eukaryota;__Alveolata;__Protalveolata;__Syndiniales;__Amoebophrya;__	0.21	0	0.15	1.26	0.22	79.43
Eukaryota;__Rhizaria;__Cercozoa;Other;Other;Other	0	0.21	0.15	1.06	0.22	79.65
Eukaryota;__Rhizaria;__Cercozoa;__Novel Clade 2;__;__	0.21	0	0.15	0.96	0.22	79.87
Bacteria;__Proteobacteria;__Gammaproteobacteria;__Order_Incertae_Sedis;__Family_Incertae_Sedis;__Marinicella	0.18	0.15	0.15	1.16	0.22	80.09

Groups Atlas Pool & Brown Lagoon

Average dissimilarity = 81.70

Species	Atlas Pool Av.Abund	Brown Lagoon Av.Abund	Av.Diss	Diss/SD	Contrib%	Cum.%
Bacteria;__Proteobacteria;__Alphaproteobacteria;__Sphingomonadales;__Sphingomonadaceae;__Sphingopyxis	0	4.06	4.01	2.03	4.91	4.91
Bacteria;__Cyanobacteria;__Chloroplast;__Chloroplast;__Chloroplast;__Chloroplast	3.27	5.14	3.67	1.42	4.5	9.41
Eukaryota;__Alveolata;__Ciliophora;__Spirotrichea;__Hypotrichia;Other	3.49	0.04	3.46	2.66	4.24	13.65
Bacteria;__Bacteroidetes;__Flavobacteria;__Flavobacteriales;__Flavobacteriaceae;__NS3a_marine_group	0	3.07	3.05	1.94	3.74	17.38
Bacteria;__Actinobacteria;__Actinobacteria;__Frankiales;__Sporichthyaceae;__hgcI_clade	2.32	0	2.48	0.91	3.04	20.42
Bacteria;__Actinobacteria;__Actinobacteria;__Micrococcales;__Microbacteriaceae;Other	1.99	0	2.07	1.15	2.53	22.94
Eukaryota;__Chloroplastida;__Chlorophyceae;__Chlamydomonas;__;__	2.25	0	2.06	1.69	2.52	25.47
Bacteria;__Bacteroidetes;__Flavobacteria;__Flavobacteriales;__Flavobacteriaceae;__Flavobacterium	2.2	0.15	1.92	2.27	2.34	27.81
Bacteria;__Proteobacteria;__Betaproteobacteria;__Burkholderiales;__Burkholderiaceae;__Polynucleobacter	1.75	0	1.84	1.05	2.25	30.06
Eukaryota;__Chloroplastida;__Chlorophyceae;Other;Other;Other	1.85	0	1.68	1.5	2.05	32.11
Bacteria;__Proteobacteria;__Betaproteobacteria;__Burkholderiales;__Comamonadaceae;Other	2.66	3.08	1.45	1.17	1.78	33.89
Bacteria;__Proteobacteria;__Alphaproteobacteria;__Rhodobacterales;__Rhodobacteraceae;Other	1.03	2.06	1.39	1.09	1.7	35.59
Eukaryota;__Chloroplastida;__Mamiellophyceae;__Micromonas;__;__	0	1.27	1.18	0.98	1.45	37.04
Bacteria;__Proteobacteria;__Betaproteobacteria;Other;Other;Other	0.64	1.33	1.12	0.91	1.37	38.41
Bacteria;__Actinobacteria;__Actinobacteria;__Frankiales;__Sporichthyaceae;Other	1.02	0	1.08	0.93	1.33	39.74
Bacteria;__Proteobacteria;__Betaproteobacteria;__Burkholderiales;__Alcaligenaceae;__Achromobacter	1.09	0	1.07	2.16	1.31	41.05
Bacteria;__Proteobacteria;__Betaproteobacteria;__Burkholderiales;__Comamonadaceae;__Polaromonas	1.23	0.12	1.03	1.81	1.26	42.31

Eukaryota;__Rhizaria;__Cercozoa;__Endomyxa;__Novel Clade 10;__	1.09	0	1.03	2.14	1.26	43.57
Bacteria;__Actinobacteria;__Acidimicrobiia;__Acidimicrobiales;__Acidimicrobiaceae;__g	0.04	0.99	0.94	2.67	1.15	44.72
Bacteria;__Proteobacteria;__Betaproteobacteria;__Burkholderiales;__Comamonadaceae;__Simplicispira	0.91	0	0.84	2.04	1.02	45.75
Bacteria;__Proteobacteria;__Alphaproteobacteria;__Sphingomonadales;__Erythrobacteraceae;__Altererythrobacter	0	0.78	0.78	1.35	0.95	46.7
Bacteria;__Actinobacteria;__Actinobacteria;__PeM15;__f;__g	0.79	0.15	0.69	1.08	0.85	47.55
Eukaryota;__Fungi;__Chytridiomycota;__;__;__	0.86	0.09	0.69	1.57	0.85	48.4
Bacteria;__Bacteroidetes;__Flavobacteria;__Flavobacteriales;__Flavobacteriaceae;__Polaribacter	0	0.68	0.67	3.86	0.82	49.22
Eukaryota;__Cryptophyceae;__Cryptomonadales;__Geminigera;__;__	0	0.66	0.65	0.79	0.8	50.02
Bacteria;__Proteobacteria;__Betaproteobacteria;__Burkholderiales;__Comamonadaceae;__Paucibacter	0.61	0	0.57	1.82	0.7	50.71
Bacteria;__Candidate_division_OP11;__c;__o;__f;__g	0.55	0.04	0.56	0.83	0.68	51.4
Eukaryota;__Rhizaria;__Cercozoa;__Endomyxa;__Phytomyxea;__Spongospora	0.59	0	0.54	2.66	0.67	52.06
Bacteria;__Actinobacteria;__Actinobacteria;__Micrococcales;__Microbacteriaceae;__Candidatus_Aquiluna	0.53	0	0.54	1.38	0.66	52.73
Eukaryota;__Stramenopiles;__Diatomea;__Bacillariophytina;__Mediophyceae;Other	0.58	0	0.52	1.33	0.64	53.37
Eukaryota;__Cryptophyceae;__Cryptomonadales;__Rhodomonas;__;__	0	0.5	0.5	0.7	0.61	53.97
Bacteria;__Proteobacteria;__Alphaproteobacteria;__Rhodobacterales;__Rhodobacteraceae;__Loktanella	0	0.49	0.49	4.95	0.6	54.57
Unassigned;Other;Other;Other;Other;Other	1.1	0.73	0.47	1.61	0.57	55.14
Bacteria;__Actinobacteria;__Actinobacteria;__Micrococcales;__Microbacteriaceae;__Candidatus_Limnoluna	0.45	0	0.47	1.17	0.57	55.71
Bacteria;__Proteobacteria;__Betaproteobacteria;__Burkholderiales;__Alcaligenaceae;Other	0.57	0.15	0.42	1.68	0.51	56.23
Bacteria;__Proteobacteria;__Gammaproteobacteria;__Pseudomonadales;__Moraxellaceae;__Psychrobacter	0.5	0.1	0.41	1.65	0.5	56.72
Bacteria;__Proteobacteria;__Betaproteobacteria;__Burkholderiales;__Comamonadaceae;__g	0.45	0.07	0.4	1.39	0.49	57.22
Eukaryota;__Chloroplastida;__Trebouxiophyceae;__;__;__	0.4	0	0.38	2.06	0.46	57.68
Bacteria;__Bacteroidetes;__Cytophagia;__Cytophagales;__Cytophagaceae;__Leadbetterella	0.14	0.5	0.37	1.07	0.46	58.13
Bacteria;__Proteobacteria;__Deltaproteobacteria;__Myxococcales;__0319-6G20;__g	0.41	0.04	0.36	2.13	0.44	58.57
Bacteria;__Proteobacteria;__Gammaproteobacteria;__Xanthomonadales;__Xanthomonadaceae;__Arenimonas	0.38	0	0.36	2.16	0.44	59.02
Eukaryota;__Stramenopiles;__T53A1;__;__;__	0.37	0	0.35	2.02	0.43	59.44
Bacteria;__Proteobacteria;__Alphaproteobacteria;__Rickettsiales;__mitochondria;__g	0.37	0	0.34	1.74	0.42	59.86
Eukaryota;__Stramenopiles;__Bicosoecida;__Siluaniidae;__Siluania;__	0.36	0	0.34	1.81	0.41	60.28
Bacteria;__Proteobacteria;__Betaproteobacteria;__Burkholderiales;__Oxalobacteraceae;__Undibacterium	0.36	0	0.33	1.8	0.41	60.68
Eukaryota;__Alveolata;__Ciliophora;__Spirotrichea;__Hypotrichia;__Gonostomum	0.29	0	0.32	0.66	0.4	61.08
Bacteria;__Proteobacteria;__Betaproteobacteria;__Burkholderiales;Other;Other	0.38	0.16	0.3	0.85	0.37	61.45
Bacteria;__Proteobacteria;__Alphaproteobacteria;__Rhodobacterales;__Rhodobacteraceae;__g	0.03	0.35	0.3	2.26	0.37	61.82
Eukaryota;__Alveolata;__Protalveolata;__Colpodellida;Other;Other	0.33	0	0.3	1.25	0.37	62.19

Bacteria;__Proteobacteria;__Betaproteobacteria;__Burkholderiales;__Comamonadaceae;__Albidiferax	0.3	0	0.29	7.38	0.36	62.55
Bacteria;__Bacteroidetes;__Bacteroidia;__Bacteroidales;__Bacteroidaceae;__Bacteroides	0.31	0.28	0.29	1.46	0.36	62.9
Bacteria;__Actinobacteria;__Actinobacteria;__Micrococcales;__Microbacteriaceae;__Candidatus_Planktoluna	0.28	0	0.29	1.05	0.36	63.26
Bacteria;__Proteobacteria;__Gammaproteobacteria;__BD7-8_marine_group;__f;__g	0	0.29	0.28	2.66	0.35	63.6
Bacteria;__Firmicutes;__Clostridia;__Clostridiales;__Ruminococcaceae;__g	0.35	0.19	0.27	1.45	0.34	63.94
Bacteria;__Fusobacteria;__Fusobacteria;__Fusobacteriales;__Fusobacteriaceae;__Fusobacterium	0.36	0.22	0.27	1.37	0.33	64.27
Bacteria;__Proteobacteria;__Alphaproteobacteria;__Rhodobacterales;__Rhodobacteraceae;__Rhodobacter	0.33	0.17	0.26	1.77	0.32	64.6
Bacteria;__Bacteroidetes;__Sphingobacteriia;__Sphingobacteriales;__NS11-12_marine_group;__g	0.41	0.17	0.26	1.36	0.32	64.92
Bacteria;__Proteobacteria;__Gammaproteobacteria;__Alteromonadales;__Pseudoalteromonadaceae;__g	0	0.29	0.26	0.66	0.32	65.24
Archaea;__Euryarchaeota;__Halobacteria;__Halobacteriales;__Deep_Sea_Hydrothermal_Vent_Gp_6(DHVEG-6);__g	0.29	0.09	0.26	0.83	0.32	65.55
Bacteria;__Proteobacteria;__Gammaproteobacteria;__Pseudomonadales;__Moraxellaceae;__Perlucidibaca	0.27	0	0.25	2.16	0.31	65.86
Eukaryota;__Stramenopiles;__Chrysophyceae;__Chromulinales;__Phaeoplaca;__	0.26	0	0.25	6.7	0.31	66.17
Eukaryota;__Alveolata;__Ciliophora;__Conthreep;__Prostomatea;__Cryptocaryon	0.26	0	0.25	4.02	0.31	66.47
Eukaryota;__Alveolata;__Ciliophora;__Conthreep;__Oligohymenophorea;Other	0.27	0	0.25	1.12	0.3	66.77
Bacteria;__Proteobacteria;__Gammaproteobacteria;__Oceanospirillales;__oc58;__g	0.27	0	0.25	1.33	0.3	67.07
Bacteria;__Bacteroidetes;__Sphingobacteriia;__Sphingobacteriales;__Chitinophagaceae;__g	0.28	0.07	0.24	1.29	0.3	67.37
Bacteria;__Proteobacteria;__Betaproteobacteria;__Burkholderiales;__Comamonadaceae;__Hydrogenophaga	0.26	0	0.24	1.1	0.29	67.66
Bacteria;__Candidate_division_TM7;__c;__o;__f;__g	0.23	0	0.24	1.66	0.29	67.95
Bacteria;__Bacteroidetes;__Sphingobacteriia;__Sphingobacteriales;__Chitinophagaceae;__Filimonas	0.26	0	0.24	1.21	0.29	68.25
Eukaryota;__Chloroplastida;__Chlorophyceae;__Desmodesmus;__;__	0.27	0.02	0.24	1.41	0.29	68.54
Bacteria;__Proteobacteria;__Alphaproteobacteria;__SAR11_clade;__Chesapeake-Delaware_Bay;__g	0	0.23	0.24	0.76	0.29	68.83
Bacteria;__Proteobacteria;__Betaproteobacteria;__Burkholderiales;__Burkholderiaceae;__Ralstonia	0.23	0	0.23	1.08	0.29	69.11
Eukaryota;__Cryptophyceae;__Cryptomonadales;__Teleaulax;__;__	0	0.23	0.22	0.66	0.27	69.39
Bacteria;__Proteobacteria;__Betaproteobacteria;__Burkholderiales;__Alcaligenaceae;__Pusillimonas	0.2	0	0.22	0.66	0.27	69.66
Bacteria;__Bacteroidetes;__Sphingobacteriia;__Sphingobacteriales;__WCHB1-69;__g	0.24	0	0.22	1.18	0.27	69.93
Eukaryota;__Metazoa;__Arthropoda;__Maxillopoda;__;__	0.24	0.02	0.22	2.92	0.27	70.2
Bacteria;__Proteobacteria;__Betaproteobacteria;__Rhodocyclales;__Rhodocyclaceae;__Dechloromonas	0.23	0	0.22	2.94	0.27	70.46
Bacteria;__Proteobacteria;__Alphaproteobacteria;__Rhodospirillales;__Rhodospirillaceae;__Thalassospira	0	0.22	0.21	3	0.26	70.72
Bacteria;__Actinobacteria;__Actinobacteria;__Micrococcales;__Microbacteriaceae;__Alpinimonas	0.19	0	0.21	0.66	0.26	70.98
Bacteria;__Bacteroidetes;__Flavobacteria;__Flavobacteriales;__Flavobacteriaceae;Other	0.03	0.22	0.2	1.37	0.25	71.23
Eukaryota;__Chloroplastida;__Chlorophyceae;__;__;__	0.22	0	0.2	1.16	0.25	71.48

Bacteria;__Proteobacteria;__Deltaproteobacteria;__Bdellovibrionales;__Bacteriovoracaceae;__Bacteriovorax	0.24	0.08	0.2	1.39	0.24	71.72
Bacteria;__WCHB1-60;__c;__o;__f;__g	0.18	0.04	0.2	0.86	0.24	71.96
Bacteria;__Proteobacteria;__Gammaproteobacteria;__Enterobacteriales;__Enterobacteriaceae;Other	0.07	0.17	0.19	0.98	0.24	72.19
Bacteria;__Proteobacteria;__Gammaproteobacteria;__Xanthomonadales;__Xanthomonadaceae;Other	0.21	0	0.19	1.13	0.23	72.43
Archaea;__Thaumarchaeota;__Marine_Group_I;__o;__f;__g	0	0.19	0.19	1.3	0.23	72.66
Archaea;__Euryarchaeota;__Halobacteria;__Halobacteriales;__Deep_Sea_Hydrothermal_Vent_Gp_6(DHVEG-6);__Candidatus_Parvarchaeum	0.19	0.1	0.19	1.02	0.23	72.89
Bacteria;__Proteobacteria;__Gammaproteobacteria;__Alteromonadales;__Colwelliaceae;__Colwellia	0.05	0.19	0.19	0.8	0.23	73.11
Bacteria;__Proteobacteria;__Betaproteobacteria;__Burkholderiales;__Comamonadaceae;__BAL58_marine_group	0	0.18	0.18	0.86	0.23	73.34
Archaea;__Euryarchaeota;__Methanomicrobia;__Methanomicrobiales;__Methanoregulaceae;__Methanoregula	0.19	0	0.18	2.03	0.22	73.56
Bacteria;__Candidate_division_OD1;__c;__o;__f;__g	0.2	0.02	0.18	1.4	0.22	73.78
Eukaryota;__Rhizaria;__Cercozoa;Other;Other;Other	0.19	0	0.17	1.28	0.21	73.99
Eukaryota;__Fungi;__Blastocladiomycota;__;__;__	0.19	0	0.17	1.17	0.21	74.2
Bacteria;__Firmicutes;__Bacilli;__Bacillales;__Staphylococcaceae;Other	0.2	0.04	0.17	1.49	0.21	74.41
Bacteria;__Proteobacteria;__Gammaproteobacteria;__Xanthomonadales;__Sinobacteraceae;__g	0.17	0	0.17	4.66	0.2	74.61
Bacteria;__Proteobacteria;__Deltaproteobacteria;__Syntrophobacterales;__Syntrophaceae;__Smithella	0.17	0	0.16	6.52	0.2	74.81
Bacteria;__Proteobacteria;__Alphaproteobacteria;__SAR11_clade;__Surface_1;__g	0	0.17	0.16	1.31	0.2	75.02
Bacteria;__Bacteroidetes;__Flavobacteria;__Flavobacteriales;__Flavobacteriaceae;__Chryseobacterium	0.18	0	0.16	1.32	0.2	75.22
Bacteria;__Proteobacteria;__Betaproteobacteria;__Burkholderiales;__Comamonadaceae;__Pelomonas	0.17	0	0.16	1.06	0.2	75.41
Bacteria;__Proteobacteria;__Betaproteobacteria;__Burkholderiales;__Comamonadaceae;__Acidovorax	0.13	0.29	0.16	1.09	0.2	75.61
Bacteria;__Proteobacteria;__Gammaproteobacteria;__Alteromonadales;__Alteromonadaceae;__BD1-7_clade	0.18	0	0.16	1.3	0.19	75.8
Bacteria;__Bacteroidetes;__Sphingobacteriia;__Sphingobacteriales;__Chitinophagaceae;__Ferruginibacter	0.17	0	0.16	1.28	0.19	75.99
Bacteria;__Bacteroidetes;__vadinHA17;__o;__f;__g	0.16	0	0.15	2.95	0.19	76.18
Bacteria;__Bacteroidetes;__Bacteroidia;__Bacteroidales;__Prevotellaceae;__Prevotella	0.19	0.09	0.15	1.32	0.19	76.36
Eukaryota;__Fungi;Other;Other;Other;Other	0.17	0	0.15	1.31	0.19	76.55
Bacteria;__Actinobacteria;Other;Other;Other;Other	0.15	0	0.15	1.01	0.18	76.74
Bacteria;__Bacteroidetes;__Bacteroidia;__Bacteroidales;__Rikenellaceae;__Alistipes	0.17	0	0.15	1.06	0.18	76.92
Eukaryota;__Alveolata;__Dinoflagellata;__Dinophyceae;__Gymnodiniphycidae;__FV18-2D9	0.16	0	0.15	1.29	0.18	77.1
Eukaryota;__Stramenopiles;__Eustigmatales;__Nannochloropsis;__;__	0.16	0	0.15	1.32	0.18	77.28
Eukaryota;__Stramenopiles;__CCI40;__;__;__	0.16	0	0.15	1.28	0.18	77.45
Bacteria;__Bacteroidetes;__Sphingobacteriia;__Sphingobacteriales;__Sphingobacteriaceae;__Pedobacter	0.17	0	0.14	0.87	0.18	77.63
Eukaryota;Other;Other;Other;Other;Other	0.09	0.17	0.14	1.04	0.18	77.81

Eukaryota;__Stramenopiles;__Pirsonia;__;__;__	0	0.16	0.14	0.66	0.18	77.98
Bacteria;__Firmicutes;__Bacilli;__Lactobacillales;__Carnobacteriaceae;__Atopostipes	0.16	0	0.14	1.27	0.17	78.16
Bacteria;__Proteobacteria;__Gammaproteobacteria;__Oceanospirillales;__SAR86_clade;__g	0	0.16	0.14	0.66	0.17	78.33
Bacteria;__Proteobacteria;__Alphaproteobacteria;__Kordiimonadales;__Kordiimonadaceae;__Kordiimonas	0	0.15	0.14	1.3	0.17	78.5
Bacteria;__Proteobacteria;__Gammaproteobacteria;__Pseudomonadales;__Pseudomonadaceae;__Pseudomonas	0.12	0.2	0.14	1.16	0.17	78.67
Eukaryota;__Alveolata;__Protalveolata;__Perkinsidae;__NA2-2D10;__	0	0.15	0.14	0.66	0.17	78.84
Bacteria;__Proteobacteria;__Betaproteobacteria;__Nitrosomonadales;__Gallionellaceae;__Gallionella	0.03	0.14	0.14	1	0.17	79.01
Bacteria;__Cyanobacteria;__Cyanobacteria;__SubsectionIII;__FamilyI;__g	0.14	0	0.13	1.06	0.16	79.17
Bacteria;__Proteobacteria;__Betaproteobacteria;__Burkholderiales;__Comamonadaceae;__Comamonas	0.15	0	0.13	1.29	0.16	79.33
Eukaryota;__Chloroplastida;__Ulvophyceae;Other;Other;Other	0	0.13	0.13	0.96	0.16	79.49
Bacteria;__Proteobacteria;__Alphaproteobacteria;__Rhodobacterales;__Rhodobacteraceae;__Roseobacter_clade_DC5-80-3_lineage	0	0.14	0.13	0.66	0.16	79.65
Bacteria;__Proteobacteria;__Betaproteobacteria;__Burkholderiales;__Comamonadaceae;__Limnohabitans	0.09	0.1	0.13	1.33	0.16	79.81
Bacteria;__Cyanobacteria;__Cyanobacteria;__SubsectionIII;__FamilyI;__Chamaesiphon	0.13	0	0.13	2.05	0.16	79.96
Bacteria;__Firmicutes;__Bacilli;__Lactobacillales;__Aerococcaceae;__Globicatella	0.14	0	0.13	1.04	0.15	80.12

Groups Seal Pool & Brown Lagoon

Average dissimilarity = 82.85

Species	Seal Pool Av.Abund	Brown Lagoon Av.Abund	Av.Diss	Diss/SD	Contrib%	Cum.%
Bacteria;__Cyanobacteria;__Chloroplast;__Chloroplast;__Chloroplast;__Chloroplast	0.84	5.14	4.13	1.46	4.98	4.98
Bacteria;__Proteobacteria;__Alphaproteobacteria;__Sphingomonadales;__Sphingomonadaceae;__Sphingopyxis	0.02	4.06	3.74	2.12	4.51	9.49
Bacteria;__Actinobacteria;__Actinobacteria;__PeM15;__f;__g	3.86	0.15	3.44	1.58	4.16	13.65
Bacteria;__Bacteroidetes;__Flavobacteria;__Flavobacteriales;__Flavobacteriaceae;__NS3a_marine_group	0	3.07	2.86	2.02	3.45	17.1
Bacteria;__Bacteroidetes;__Flavobacteria;__Flavobacteriales;__Flavobacteriaceae;__Flavobacterium	3.15	0.15	2.7	1.85	3.26	20.36
Bacteria;__Proteobacteria;__Betaproteobacteria;__Burkholderiales;__Burkholderiaceae;__Polynucleobacter	2.03	0	1.91	1.17	2.31	22.67
Bacteria;__Proteobacteria;__Betaproteobacteria;__Burkholderiales;__Comamonadaceae;__Polaromonas	2	0.12	1.7	2.16	2.05	24.72
Bacteria;__Proteobacteria;__Alphaproteobacteria;__Rhodobacterales;__Rhodobacteraceae;Other	1.79	2.06	1.47	1.35	1.77	26.49

Bacteria;__Actinobacteria;__Actinobacteria;__Frankiales;__Sporichthyaceae;__hgcI_clade	1.22	0	1.15	1.07	1.39	27.87
Bacteria;__Proteobacteria;__Betaproteobacteria;__Burkholderiales;__Comamonadaceae;Other	2.78	3.08	1.14	1.34	1.38	29.25
Bacteria;__Proteobacteria;__Betaproteobacteria;__Burkholderiales;__Comamonadaceae;__Simplicispira	1.25	0	1.12	1.69	1.35	30.6
Eukaryota;__Chloroplastida;__Mamiellophyceae;__Micromonas;__;__	0	1.27	1.11	0.99	1.34	31.95
Bacteria;__Actinobacteria;__Actinobacteria;__Micrococcales;__Microbacteriaceae;Other	1.18	0	1.11	1.34	1.34	33.28
Bacteria;__Proteobacteria;__Betaproteobacteria;Other;Other;Other	0.54	1.33	1	0.8	1.21	34.49
Eukaryota;__Rhizaria;__Cercozoa;__Thecofilosea;Other;Other	1.1	0	0.99	1.78	1.2	35.69
Bacteria;__Bacteroidetes;__Sphingobacteriia;__Sphingobacteriales;__Sphingobacteriaceae;__Pedobacter	1.06	0	0.98	1.95	1.18	36.86
Bacteria;__Proteobacteria;__Betaproteobacteria;__Burkholderiales;__Comamonadaceae;__g	1.15	0.07	0.97	1.29	1.17	38.04
Bacteria;__Bacteroidetes;__Sphingobacteriia;__Sphingobacteriales;__Chitinophagaceae;__Sediminibacterium	1.15	0.09	0.96	2.31	1.16	39.2
Bacteria;__Actinobacteria;__Actinobacteria;__Frankiales;__Sporichthyaceae;Other	1.02	0	0.96	1.05	1.16	40.36
Eukaryota;__Chloroplastida;__Chlorophyceae;__Desmodesmus;__;__	1.02	0.02	0.9	1.09	1.09	41.45
Bacteria;__Proteobacteria;__Betaproteobacteria;__Burkholderiales;__Comamonadaceae;__Paucibacter	0.97	0	0.87	1.24	1.05	42.5
Bacteria;__Bacteroidetes;__Sphingobacteriia;__Sphingobacteriales;__NS11-12_marine_group;__g	1.14	0.17	0.87	1.23	1.05	43.54
Bacteria;__Actinobacteria;__Acidimicrobiia;__Acidimicrobiales;__Acidimicrobiaceae;__g	0.13	0.99	0.8	2.51	0.96	44.51
Bacteria;__Bacteroidetes;__Sphingobacteriia;__Sphingobacteriales;__Chitinophagaceae;__Ferruginibacter	0.86	0	0.77	1.53	0.93	45.44
Bacteria;__Proteobacteria;__Alphaproteobacteria;__Sphingomonadales;__Erythrobacteraceae;__Altererythrobacter	0	0.78	0.73	1.38	0.88	46.32
Bacteria;__Actinobacteria;Other;Other;Other;Other	0.78	0	0.73	1.66	0.88	47.2
Bacteria;__Bacteroidetes;__Flavobacteria;__Flavobacteriales;__Cryomorphaceae;__Fluviicola	0.9	0.14	0.69	2.32	0.83	48.03
Bacteria;__Proteobacteria;__Betaproteobacteria;__Burkholderiales;__Comamonadaceae;__Albidiferax	0.77	0	0.69	1.48	0.83	48.86
Bacteria;__Proteobacteria;__Gammaproteobacteria;__Pseudomonadales;__Moraxellaceae;__Perlucidibaca	0.76	0	0.68	2.84	0.83	49.69
Bacteria;__Bacteroidetes;__Sphingobacteriia;__Sphingobacteriales;__Saprospiraceae;__g	0.76	0.04	0.66	0.93	0.8	50.48
Bacteria;__Candidate_division_OP11;__c;__o;__f;__g	0.72	0.04	0.64	1.12	0.77	51.26
Bacteria;__Proteobacteria;__Alphaproteobacteria;__Rhodospirillales;__Acetobacteraceae;__Roseococcus	0.71	0	0.64	1.39	0.77	52.02
Bacteria;__Proteobacteria;__Betaproteobacteria;__Burkholderiales;__Alcaligenaceae;__Achromobacter	0.67	0	0.62	2.45	0.75	52.77
Eukaryota;__Cryptophyceae;__Cryptomonadales;__Geminigera;__;__	0	0.66	0.61	0.8	0.74	53.51
Bacteria;__Bacteroidetes;__Sphingobacteriia;__Sphingobacteriales;__Chitinophagaceae;__g	0.71	0.07	0.6	1.19	0.72	54.24
Bacteria;__Actinobacteria;__Actinobacteria;__Micrococcales;__Microbacteriaceae;__Candidatus_Aquiluna	0.62	0	0.57	1.62	0.69	54.93
Bacteria;__Bacteroidetes;__Flavobacteria;__Flavobacteriales;__Flavobacteriaceae;__Polaribacter	0.06	0.68	0.57	3.28	0.69	55.62
Archaea;__Euryarchaeota;__Methanomicrobia;__Methanomicrobiales;__Methanoregulaceae;__Methanoregula	0.61	0	0.56	13.97	0.68	56.29
Eukaryota;__Fungi;__Chytridiomycota;__;__;__	0.63	0.09	0.52	1.19	0.62	56.92

Bacteria;__Proteobacteria;__Alphaproteobacteria;__Sphingomonadales;__Sphingomonadaceae;__Novosphingobium	0.68	0.11	0.51	1.72	0.62	57.53
Eukaryota;__Cryptophyceae;__Cryptomonadales;__Rhodomonas;__;__	0	0.5	0.47	0.71	0.56	58.1
Bacteria;__Actinobacteria;__Acidimicrobiia;__Acidimicrobiales;__Acidimicrobiaceae;__CL500-29_marine_group	0.49	0	0.46	0.97	0.56	58.66
Bacteria;__Proteobacteria;__Alphaproteobacteria;__Rhodobacterales;__Rhodobacteraceae;__Loktanella	0	0.49	0.46	6.25	0.55	59.21
Bacteria;__Proteobacteria;__Betaproteobacteria;__Burkholderiales;__Alcaligenaceae;Other	0.64	0.15	0.44	1.81	0.54	59.74
Bacteria;__Proteobacteria;__Gammaproteobacteria;__Pseudomonadales;__Moraxellaceae;__Psychrobacter	0.53	0.1	0.42	1.8	0.5	60.24
Bacteria;__Bacteroidetes;__Cytophagia;__Cytophagales;__Cytophagaceae;__Leadbetterella	0.5	0.5	0.4	1.27	0.49	60.73
Bacteria;__Bacteroidetes;__Sphingobacteriia;__Sphingobacteriales;__WCHB1-69;__g	0.44	0	0.39	1.14	0.48	61.2
Bacteria;__Candidate_division_TM7;__c;__o;__f;__g	0.41	0	0.39	0.84	0.48	61.68
Bacteria;__Proteobacteria;__Deltaproteobacteria;__Bdellovibrionales;__Bacteriovoracaceae;__Bacteriovorax	0.5	0.08	0.38	2.8	0.46	62.14
Bacteria;__Actinobacteria;__Acidimicrobiia;__Acidimicrobiales;__uncultured;__g	0.41	0	0.38	2.92	0.45	62.59
Bacteria;__Bacteroidetes;__Sphingobacteriia;__Sphingobacteriales;__Chitinophagaceae;__Filimonas	0.42	0	0.38	0.94	0.45	63.05
Archaea;__Euryarchaeota;__Halobacteria;__Halobacteriales;__Deep_Sea_Hydrothermal_Vent_Gp_6(DHVEG-6);__g	0.41	0.09	0.36	0.98	0.44	63.49
Bacteria;__Proteobacteria;__Gammaproteobacteria;__Oceanospirillales;__oc58;__g	0.39	0	0.36	2.16	0.43	63.92
Unassigned;Other;Other;Other;Other;Other	0.91	0.73	0.36	1.21	0.43	64.35
Bacteria;__WCHB1-60;__c;__o;__f;__g	0.37	0.04	0.34	0.8	0.41	64.76
Bacteria;__Proteobacteria;__Betaproteobacteria;__Burkholderiales;Other;Other	0.53	0.16	0.33	1.8	0.4	65.16
Bacteria;__Verrucomicrobia;__Opitutae;__Opitutales;__Opitutaceae;__Opitutus	0.36	0	0.32	1.49	0.39	65.55
Bacteria;__Proteobacteria;__Alphaproteobacteria;__Rhodobacterales;__Rhodobacteraceae;__g	0	0.35	0.32	2.91	0.38	65.93
Bacteria;__Gemmatimonadetes;__Gemmatimonadetes;__Gemmatimonadales;__Gemmatimonadaceae;__Gemmatimonas	0.35	0	0.31	1.72	0.38	66.31
Bacteria;__Bacteroidetes;__vadinHA17;__o;__f;__g	0.34	0	0.31	2.55	0.37	66.68
Bacteria;__Candidate_division_OD1;__c;__o;__f;__g	0.3	0.02	0.3	0.71	0.36	67.03
Bacteria;__Proteobacteria;__Deltaproteobacteria;__Myxococcales;__0319-6G20;__g	0.36	0.04	0.29	1.36	0.35	67.39
Bacteria;__Proteobacteria;__Gammaproteobacteria;__BD7-8_marine_group;__f;__g	0	0.29	0.27	2.85	0.32	67.71
Bacteria;__Fusobacteria;__Fusobacteria;__Fusobacteriales;__Fusobacteriaceae;__Fusobacterium	0.38	0.22	0.27	1.34	0.32	68.03
Bacteria;__Proteobacteria;__Betaproteobacteria;__Burkholderiales;__Comamonadaceae;__Acidovorax	0.38	0.29	0.26	1.92	0.31	68.34
Bacteria;__Proteobacteria;__Gammaproteobacteria;__Alteromonadales;__Alteromonadaceae;__BD1-7_clade	0.29	0	0.26	1.52	0.31	68.65
Bacteria;__Proteobacteria;__Betaproteobacteria;__Burkholderiales;__Comamonadaceae;__Rhizobacter	0.28	0	0.26	2.45	0.31	68.96
Bacteria;__Proteobacteria;__Betaproteobacteria;__Burkholderiales;__Oxalobacteraceae;__Undibacterium	0.28	0	0.25	3.35	0.31	69.26

Bacteria;__Bacteroidetes;__Bacteroidia;__Bacteroidales;__Bacteroidaceae;__Bacteroides	0.21	0.28	0.25	1.2	0.3	69.57
Archaea;__Euryarchaeota;__Halobacteria;__Halobacteriales;__Deep_Sea_Hydrothermal_Vent_Gp_6(DHVEG-6);__Candidatus_Parvarchaeum	0.3	0.1	0.25	1.19	0.3	69.87
Bacteria;__Proteobacteria;__Gammaproteobacteria;__Alteromonadales;__Pseudoalteromonadaceae;__g	0	0.29	0.25	0.67	0.3	70.16
Bacteria;__Proteobacteria;__Alphaproteobacteria;__Sphingomonadales;__Sphingomonadaceae;__Sandarakinorhabdus	0.27	0	0.24	1.26	0.3	70.46
Bacteria;__Proteobacteria;__Betaproteobacteria;__Neisseriales;__Neisseriaceae;__g	0.27	0	0.24	2.14	0.29	70.75
Bacteria;__Proteobacteria;__Alphaproteobacteria;__Rhodobacterales;__Rhodobacteraceae;__Rhodobacter	0.32	0.17	0.24	1.35	0.29	71.04
Eukaryota;__Fungi;__LKM11;__;__;__	0.26	0	0.24	2.69	0.29	71.32
Bacteria;__Bacteroidetes;__Sphingobacteriia;__Sphingobacteriales;__env.OPS_17;__g	0.25	0	0.22	1.86	0.27	71.59
Bacteria;__Proteobacteria;__Alphaproteobacteria;__SAR11_clade;__Chesapeake-Delaware_Bay;__g	0	0.23	0.22	0.77	0.27	71.86
Eukaryota;__Stramenopiles;__T53A1;__;__;__	0.24	0	0.22	1.81	0.27	72.13
Bacteria;__Proteobacteria;Other;Other;Other;Other	0.24	0	0.22	3.25	0.27	72.39
Eukaryota;__Cryptophyceae;__Cryptomonadales;__Teleaulax;__;__	0	0.23	0.21	0.67	0.25	72.65
Eukaryota;__Fungi;Other;Other;Other;Other	0.23	0	0.21	1.82	0.25	72.9
Bacteria;__Firmicutes;__Clostridia;__Clostridiales;__Ruminococcaceae;__g	0.24	0.19	0.2	1.37	0.24	73.14
Bacteria;__Proteobacteria;__Betaproteobacteria;__Burkholderiales;__Comamonadaceae;__Comamonas	0.22	0	0.2	1.1	0.24	73.38
Bacteria;__Proteobacteria;__Alphaproteobacteria;__Rhodospirillales;__Rhodospirillaceae;__Thalassospira	0	0.22	0.2	3.24	0.24	73.63
Eukaryota;__Stramenopiles;__Chrysophyceae;Other;Other;Other	0.22	0	0.2	3.37	0.24	73.86
Bacteria;__Proteobacteria;__Gammaproteobacteria;__Enterobacteriales;__Enterobacteriaceae;Other	0.13	0.17	0.2	1.33	0.24	74.1
Bacteria;__Bacteroidetes;__Bacteroidia;__Bacteroidales;__Prevotellaceae;__Prevotella	0.24	0.09	0.19	1.12	0.23	74.33
Eukaryota;__Chloroplastida;__Chlorophyceae;__Chlamydomonas;__;__	0.21	0	0.19	1.03	0.23	74.56
Bacteria;__Bacteroidetes;__Flavobacteria;__Flavobacteriales;__Flavobacteriaceae;Other	0.05	0.22	0.19	1.3	0.23	74.79
Bacteria;__Proteobacteria;__Betaproteobacteria;__Nitrosomonadales;__Nitrosomonadaceae;__Nitrosomonas	0.21	0.03	0.19	0.94	0.23	75.01
Bacteria;__Firmicutes;__Bacilli;__Bacillales;__Staphylococcaceae;Other	0.23	0.04	0.19	1.27	0.22	75.24
Bacteria;__Actinobacteria;__Actinobacteria;Other;Other;Other	0.19	0	0.18	1.24	0.22	75.46
Archaea;__Thaumarchaeota;__Marine_Group_I;__o;__f;__g	0	0.19	0.18	1.33	0.21	75.67
Bacteria;__Proteobacteria;__Betaproteobacteria;__Burkholderiales;__Comamonadaceae;__BAL58_marine_group	0	0.18	0.17	0.87	0.21	75.88
Bacteria;__Bacteroidetes;Other;Other;Other;Other	0.19	0	0.17	2.25	0.21	76.08
Bacteria;__Proteobacteria;__Alphaproteobacteria;__Rhodobacterales;__Rhodobacteraceae;__Paracoccus	0.18	0.04	0.17	0.82	0.2	76.29
Bacteria;__Proteobacteria;__Gammaproteobacteria;__Oceanospirillales;__Oceanospirillaceae;__Pseudospirillum	0.18	0	0.17	1.27	0.2	76.49
Bacteria;Other;Other;Other;Other;Other	0.19	0	0.17	1.66	0.2	76.69

Bacteria;__Bacteroidetes;__Flavobacteria;__Flavobacteriales;__NS9_marine_group;__g	0.21	0.07	0.16	1.53	0.2	76.89
Bacteria;__Actinobacteria;__Actinobacteria;__Corynebacteriales;__Mycobacteriaceae;__Mycobacterium	0.18	0	0.16	1.09	0.2	77.08
Eukaryota;__Alveolata;__Ciliophora;__Conthreep;__Oligohymenophorea;Other	0.17	0	0.16	1.31	0.19	77.28
Bacteria;__Proteobacteria;__Gammaproteobacteria;__Alteromonadales;__Colwelliaceae;__Colwellia	0	0.19	0.16	0.67	0.19	77.47
Bacteria;__Verrucomicrobia;__Verrucomicrobiae;__Verrucomicrobiales;__Verrucomicrobiaceae;__g	0.17	0	0.16	3.01	0.19	77.66
Eukaryota;Other;Other;Other;Other;Other	0	0.17	0.16	1.02	0.19	77.85
Bacteria;__Bacteroidetes;__Sphingobacteriia;__Sphingobacteriales;Other;Other	0.17	0	0.16	3.91	0.19	78.04
Bacteria;__Proteobacteria;__Deltaproteobacteria;__Syntrophobacterales;__Syntrophaceae;__Smithella	0.16	0	0.16	0.67	0.19	78.23
Bacteria;__Proteobacteria;__Gammaproteobacteria;__Oceanospirillales;__SAR86_clade;__g	0.06	0.16	0.15	0.91	0.19	78.42
Bacteria;__Proteobacteria;__Alphaproteobacteria;__Rhizobiales;__Hyphomicrobiaceae;__Devosia	0.17	0	0.15	1.32	0.19	78.6
Bacteria;__Bacteroidetes;__WCHB1-32;__o;__f;__g	0.17	0	0.15	1.22	0.18	78.79
Bacteria;__Proteobacteria;__Betaproteobacteria;__Rhodocyclales;__Rhodocyclaceae;Other	0.16	0	0.15	3.29	0.18	78.96
Bacteria;__Proteobacteria;__Alphaproteobacteria;__Rhodobacterales;__Rhodobacteraceae;__Roseobacter_clade_DC5-80-3_lineage	0.07	0.14	0.14	0.94	0.17	79.14
Bacteria;__Proteobacteria;__Alphaproteobacteria;__Caulobacterales;__Caulobacteraceae;__Phenylobacterium	0.16	0	0.14	1.33	0.17	79.31
Bacteria;__Proteobacteria;__Deltaproteobacteria;__Desulfuromonadales;Other;Other	0.16	0	0.14	1.33	0.17	79.48
Bacteria;__Acidobacteria;__Holophagae;__Holophagales;__Holophagaceae;__Geothrix	0.16	0	0.14	2.38	0.17	79.65
Bacteria;__Proteobacteria;__Alphaproteobacteria;__SAR11_clade;__Surface_1;__g	0.06	0.17	0.14	1.26	0.16	79.82
Bacteria;__Proteobacteria;__Betaproteobacteria;__Nitrosomonadales;__Gallionellaceae;__Gallionella	0	0.14	0.14	1.03	0.16	79.98
Eukaryota;__Stramenopiles;__Pirsonia;__;__;__	0	0.16	0.14	0.67	0.16	80.14

Groups Atlas Cove & Brown Lagoon

Average dissimilarity = 83.55

Species	Atlas Cove Av.Abund	Brown Lagoon Av.Abund	Av.Diss	Diss/SD	Contrib%	Cum.%
Bacteria;__Cyanobacteria;__Chloroplast;__Chloroplast;__Chloroplast;__Chloroplast	2.38	5.14	3.87	1.53	4.63	4.63
Bacteria;__Proteobacteria;__Alphaproteobacteria;__Sphingomonadales;__Sphingomonadaceae;__Sphingopyxis	0.26	4.06	3.68	1.88	4.4	9.03
Bacteria;__Proteobacteria;__Gammaproteobacteria;__Oceanospirillales;__ZD0405;__g	3.19	0.12	3.09	1.8	3.7	12.73
Bacteria;__Proteobacteria;__Betaproteobacteria;__Burkholderiales;__Comamonadaceae;Other	0.03	3.08	2.93	2.61	3.51	16.24
Bacteria;__Proteobacteria;__Gammaproteobacteria;Other;Other;Other	3.15	0.13	2.88	2.66	3.45	19.69
Bacteria;__Proteobacteria;__Alphaproteobacteria;__SAR11_clade;__Surface_1;__g	2.82	0.17	2.71	1.27	3.25	22.94

Bacteria;__Bacteroidetes;__Flavobacteria;__Flavobacteriales;__Flavobacteriaceae;__NS3a_marine_group	0.38	3.07	2.61	1.72	3.13	26.06
Bacteria;__Proteobacteria;__Gammaproteobacteria;__Alteromonadales;__Alteromonadaceae;__SAR92_clade	2.09	0.13	1.86	2.21	2.23	28.29
Bacteria;__Proteobacteria;__Gammaproteobacteria;__Oceanospirillales;__SAR86_clade;__g	1.99	0.16	1.83	2.03	2.19	30.48
Bacteria;__Bacteroidetes;__Flavobacteria;__Flavobacteriales;__Flavobacteriaceae;__NS2b_marine_group	1.92	0.1	1.77	3.25	2.12	32.61
Bacteria;__Proteobacteria;__Alphaproteobacteria;__Rhodobacterales;__Rhodobacteraceae;Other	0.29	2.06	1.69	1.15	2.02	34.63
Bacteria;__Proteobacteria;__Alphaproteobacteria;__Rhodobacterales;__Rhodobacteraceae;__Roseobacter_clade_DC5-80-3_lineage	1.59	0.14	1.43	2.33	1.71	36.34
Bacteria;__Bacteroidetes;__Flavobacteria;__Flavobacteriales;__NS9_marine_group;__g	1.54	0.07	1.39	7.28	1.67	38.01
Archaea;__Euryarchaeota;__Thermoplasmata;__Thermoplasmatales;__Marine_Group_II;__g	1.28	0.06	1.23	1.77	1.48	39.48
Bacteria;__Bacteroidetes;__Flavobacteria;__Flavobacteriales;__Flavobacteriaceae;__NS4_marine_group	1.31	0.1	1.23	1.64	1.47	40.96
Bacteria;__Proteobacteria;__Betaproteobacteria;Other;Other;Other	0.45	1.33	1.04	0.76	1.25	42.2
Eukaryota;__Chloroplastida;__Mamiellophyceae;__Micromonas;__;__	0.33	1.27	1	0.9	1.2	43.41
Bacteria;__Proteobacteria;__Gammaproteobacteria;__Alteromonadales;__Pseudoalteromonadaceae;__Pseudoalteromonas	1.18	0.03	1	1.51	1.2	44.6
Bacteria;__Bacteroidetes;__Flavobacteria;__Flavobacteriales;__Flavobacteriaceae;__Polaribacter	1.7	0.68	0.97	2.72	1.16	45.76
Bacteria;__Proteobacteria;__Gammaproteobacteria;__Alteromonadales;__Alteromonadaceae;__Alteromonas	1.1	0	0.96	1.33	1.14	46.9
Bacteria;__Actinobacteria;__Acidimicrobiia;__Acidimicrobiales;__Acidimicrobiaceae;__g	0.04	0.99	0.91	2.65	1.09	47.99
Eukaryota;__Stramenopiles;__MAST-1;__MAST-1 B;__;__	1	0	0.88	1.3	1.05	49.04
Bacteria;__Proteobacteria;__Alphaproteobacteria;__SAR11_clade;Other;Other	0.9	0.07	0.84	1.51	1	50.04
Bacteria;__Bacteroidetes;__Flavobacteria;__Flavobacteriales;__Flavobacteriaceae;Other	1.01	0.22	0.8	1.27	0.96	51
Bacteria;__Proteobacteria;__Alphaproteobacteria;__Sphingomonadales;__Erythrobacteraceae;__Altererythrobacter	0	0.78	0.76	1.34	0.91	51.91
Bacteria;__Bacteroidetes;__Flavobacteria;__Flavobacteriales;Other;Other	0.84	0	0.76	2.51	0.91	52.82
Eukaryota;__Haptophyta;__Prymnesiophyceae;Other;Other;Other	0.86	0.02	0.74	1.37	0.89	53.71
Eukaryota;Other;Other;Other;Other;Other	0.97	0.17	0.73	0.85	0.88	54.59
Eukaryota;__Metazoa;__Arthropoda;__Maxillopoda;__;__	0.78	0.02	0.64	0.82	0.76	55.35
Eukaryota;__Alveolata;__Dinoflagellata;__Dinophyceae;__Gymnodiniphycidae;__Gyrodinium	0.77	0	0.63	0.75	0.76	56.11
Eukaryota;__Cryptophyceae;__Cryptomonadales;__Geminigera;__;__	0.13	0.66	0.63	0.84	0.76	56.86
Bacteria;__Proteobacteria;__Alphaproteobacteria;__Rhodobacterales;__Rhodobacteraceae;__Sulfitobacter	0.73	0.03	0.63	1.37	0.75	57.61
Bacteria;__Proteobacteria;__Gammaproteobacteria;__Oceanospirillales;__Alcanivoracaceae;__Alcanivorax	0.67	0	0.6	2.55	0.72	58.33
Eukaryota;__Picozoa;__;__;__;__	0.68	0	0.58	1.29	0.7	59.03
Bacteria;__Deferribacteres;__Deferribacteres;__Deferribacterales;__SAR406_clade(Marine_group_A);__g	0.55	0	0.58	1.15	0.69	59.72

Bacteria;__Proteobacteria;__Gammaproteobacteria;__Oceanospirillales;__Oceanospirillaceae;Other	0.68	0.09	0.56	3.57	0.67	60.39
Bacteria;__Bacteroidetes;__Flavobacteria;__Flavobacteriales;__Flavobacteriaceae;__NS5_marine_group	0.6	0.07	0.56	1.09	0.67	61.06
Eukaryota;__Stramenopiles;__MAST-7;__;__;__	0.62	0	0.55	1.25	0.66	61.71
Eukaryota;__Stramenopiles;__MAST-3;__;__;__	0.61	0	0.53	1.3	0.64	62.35
Eukaryota;__Alveolata;__Ciliophora;__Spirotrichea;__Oligotrichia;__uncultured	0.66	0.05	0.53	1.09	0.64	62.99
Bacteria;__Proteobacteria;__Alphaproteobacteria;__Rickettsiales;__SAR116_clade;__g	0.57	0.05	0.52	2.22	0.63	63.61
Bacteria;__Proteobacteria;__Gammaproteobacteria;__Oceanospirillales;__OM182_clade;__g	0.55	0.03	0.49	2.07	0.58	64.2
Eukaryota;__Cryptophyceae;__Cryptomonadales;__Rhodomonas;__;__	0	0.5	0.49	0.7	0.58	64.78
Bacteria;__Proteobacteria;__Alphaproteobacteria;__Rhodobacterales;__Rhodobacteraceae;__g	0.85	0.35	0.48	1.25	0.58	65.35
Bacteria;__Bacteroidetes;__Cytophagia;__Cytophagales;__Cytophagaceae;__Leadbetterella	0	0.5	0.48	1.33	0.57	65.93
Bacteria;__Proteobacteria;__Alphaproteobacteria;__Rhodobacterales;__Rhodobacteraceae;__Loktanella	0	0.49	0.47	4.72	0.57	66.49
Bacteria;__Proteobacteria;__Alphaproteobacteria;__SAR11_clade;__Surface_4;__g	0.49	0.02	0.47	1.51	0.56	67.06
Unassigned;Other;Other;Other;Other;Other	1.01	0.73	0.47	1.26	0.56	67.62
Archaea;__Thaumarchaeota;__Marine_Group_I;__o;__f;__g	0.65	0.19	0.47	1.05	0.56	68.19
Bacteria;__Proteobacteria;__Gammaproteobacteria;__Alteromonadales;__Colwelliaceae;__Colwellia	0.61	0.19	0.47	1.41	0.56	68.75
Bacteria;__Proteobacteria;__Gammaproteobacteria;__Pseudomonadales;__Moraxellaceae;__Perlucidibaca	0.49	0	0.42	1.38	0.51	69.26
Eukaryota;__Alveolata;__Protalveolata;__Syndiniales;__Syndiniales Group I;__	0.48	0	0.42	1.31	0.5	69.76
Bacteria;__Bacteroidetes;__Flavobacteria;__Flavobacteriales;__Cryomorphaceae;__Owenweeksia	0.47	0.07	0.4	1.68	0.48	70.24
Eukaryota;__Holozoa;__Choanomonada;__Acanthoecida;__Diaphanoeca;__	0.45	0	0.38	0.95	0.45	70.69
Eukaryota;__Alveolata;__Ciliophora;__Spirotrichea;__Oligotrichia;__Strombidium	0.47	0.11	0.37	0.88	0.44	71.13
Eukaryota;__Alveolata;__Protalveolata;__Syndiniales;__Syndiniales Group II;__	0.41	0	0.36	1.28	0.43	71.56
Bacteria;__Proteobacteria;__Alphaproteobacteria;Other;Other;Other	0.42	0.08	0.34	1.39	0.41	71.97
Eukaryota;__Stramenopiles;__MAST-1;__MAST-1 A;__;__	0.4	0	0.34	1.32	0.41	72.38
Bacteria;__Proteobacteria;__Deltaproteobacteria;__SAR324_clade(Marine_group_B);__f;__g	0.35	0.02	0.34	1.19	0.41	72.79
Bacteria;__Proteobacteria;__Alphaproteobacteria;__SAR11_clade;__f;__g	0.32	0	0.33	1.13	0.39	73.18
Eukaryota;__Alveolata;__Dinoflagellata;__Dinophyceae;__Gymnodiniphycidae;Other	0.38	0.08	0.32	0.91	0.39	73.57
Bacteria;__Proteobacteria;__Gammaproteobacteria;__Alteromonadales;__Alteromonadaceae;Other	0.45	0.1	0.32	2.11	0.38	73.95
Bacteria;__Bacteroidetes;__Cytophagia;__Cytophagales;__Flammeovirgaceae;__Marinoscillum	0.33	0.04	0.32	0.82	0.38	74.33
Eukaryota;__Alveolata;__Ciliophora;__Spirotrichea;__Choreotrichia;__uncultured	0.37	0	0.31	0.86	0.37	74.7
Eukaryota;__Alveolata;__Dinoflagellata;__Dinophyceae;__Gymnodiniphycidae;__Gymnodinium	0.39	0.02	0.31	0.88	0.37	75.07
Bacteria;__Bacteroidetes;__Flavobacteria;__Flavobacteriales;__Flavobacteriaceae;__g	0.39	0.06	0.3	2.59	0.36	75.43
Bacteria;__Proteobacteria;__Gammaproteobacteria;__Oceanospirillales;Other;Other	0.3	0	0.3	2.15	0.36	75.8
Bacteria;__Proteobacteria;__Gammaproteobacteria;__Alteromonadales;__Pseudoalteromonadaceae;__g	0.07	0.29	0.28	0.82	0.33	76.13
Eukaryota;__Rhizaria;__Cercozoa;__Thecofilosea;__Cryomonadida;__Cryothecomonas	0.34	0.08	0.28	1	0.33	76.46
Bacteria;__Proteobacteria;__Betaproteobacteria;__Burkholderiales;__Comamonadaceae;__Acidovorax	0	0.29	0.28	1.83	0.33	76.8

Eukaryota;__Metazoa;__Cnidaria;__Hydrozoa;__;__	0.33	0.02	0.28	0.72	0.33	77.13
Bacteria;__Bacteroidetes;__Cytophagia;__Cytophagales;__Flammeovirgaceae;Other	0.27	0	0.26	1.88	0.31	77.44
Eukaryota;__Stramenopiles;__MAST-8;__;__;__	0.29	0	0.26	1.22	0.31	77.75
Bacteria;__Bacteroidetes;__Bacteroidia;__Bacteroidales;__Bacteroidaceae;__Bacteroides	0.04	0.28	0.25	1.14	0.3	78.05
Bacteria;__Proteobacteria;Other;Other;Other;Other	0.25	0	0.24	2.64	0.29	78.35
Bacteria;__Proteobacteria;__Gammaproteobacteria;__Oceanospirillales;__Oceanospirillaceae;__Balneatrix	0.25	0	0.24	1.63	0.29	78.63
Bacteria;__Proteobacteria;__Alphaproteobacteria;__SAR11_clade;__Chesapeake-Delaware_Bay;__g	0.08	0.23	0.24	0.99	0.29	78.92
Eukaryota;__Haptophyta;__Prymnesiophyceae;__Prymnesiales;Other;Other	0.27	0	0.24	1.33	0.28	79.21
Eukaryota;__Cryptophyceae;__Cryptomonadales;__Teleaulax;__;__	0.07	0.23	0.24	0.8	0.28	79.49
Bacteria;Other;Other;Other;Other;Other	0.25	0	0.23	4.29	0.27	79.76
Eukaryota;__Metazoa;__Annelida;__;__;__	0.27	0	0.22	1.01	0.27	80.03

Groups Winston Lagoon & Brown Lagoon

Average dissimilarity = 66.06

Species	Winston Lagoon Av.Abund	Brown Lagoon Av.Abund	Av.Diss	Diss/SD	Contrib%	Cum.%
Bacteria;__Cyanobacteria;__Chloroplast;__Chloroplast;__Chloroplast;__Chloroplast	3.37	5.14	3.66	1.42	5.53	5.53
Bacteria;__Proteobacteria;__Alphaproteobacteria;__Sphingomonadales;__Sphingomonadaceae;__Sphingopyxis	1.75	4.06	3.02	1.7	4.57	10.11
Bacteria;__Proteobacteria;__Betaproteobacteria;__Burkholderiales;__Comamonadaceae;Other	1.03	3.08	2.22	1.61	3.36	13.47
Bacteria;__Bacteroidetes;__Flavobacteria;__Flavobacteriales;__Flavobacteriaceae;__NS3a_marine_group	0.9	3.07	2.18	1.27	3.3	16.77
Bacteria;__Proteobacteria;__Alphaproteobacteria;__SAR11_clade;__Surface_1;__g	2.12	0.17	2.05	0.73	3.1	19.88
Bacteria;__Bacteroidetes;__Flavobacteria;__Flavobacteriales;__Flavobacteriaceae;Other	2.34	0.22	1.87	0.88	2.83	22.71
Bacteria;__Proteobacteria;__Alphaproteobacteria;__Rhodobacterales;__Rhodobacteraceae;Other	1.87	2.06	1.53	1.46	2.32	25.03
Archaea;__Thaumarchaeota;__Marine_Group_I;__o;__f;__g	1.55	0.19	1.41	0.82	2.13	27.16
Bacteria;__Proteobacteria;__Gammaproteobacteria;__Oceanospirillales;__SAR86_clade;__g	1.39	0.16	1.32	0.93	1.99	29.15
Bacteria;__Proteobacteria;__Gammaproteobacteria;__Oceanospirillales;__ZD0405;__g	1.34	0.12	1.28	0.94	1.94	31.09
Bacteria;__Proteobacteria;__Betaproteobacteria;Other;Other;Other	0.68	1.33	1.23	1.01	1.86	32.95
Eukaryota;__Chloroplastida;__Mamiellophyceae;__Micromonas;__;__	0.8	1.27	1.16	1.18	1.75	34.7
Archaea;__Euryarchaeota;__Halobacteria;__Halobacteriales;__Deep_Sea_Hydrothermal_Vent_Gp_6(DHVEG-6);__Candidatus_Parvarchaeum	1.11	0.1	1.04	0.77	1.58	36.27
Bacteria;__Proteobacteria;__Gammaproteobacteria;__Alteromonadales;__Pseudoalteromonadaceae;__g	1.15	0.29	0.95	1.53	1.44	37.71

Bacteria;__Actinobacteria;__Actinobacteria;__Corynebacteriales;__Nocardiaceae;__Rhodococcus	0.96	0.13	0.82	1.09	1.24	38.96
Bacteria;__Proteobacteria;__Gammaproteobacteria;__Alteromonadales;__Colwelliaceae;__Colwellia	0.95	0.19	0.8	1.59	1.21	40.16
Bacteria;__Actinobacteria;__Acidimicrobiia;__Acidimicrobiales;__Acidimicrobiaceae;__g	0.54	0.99	0.78	2.19	1.18	41.35
Unassigned;Other;Other;Other;Other;Other	1.43	0.73	0.71	1.44	1.07	42.42
Archaea;__Euryarchaeota;__Halobacteria;__Halobacteriales;__Deep_Sea_Hydrothermal_Vent_Gp_6(DHVEG-6);__g	0.68	0.09	0.67	0.8	1.01	43.43
Bacteria;__Proteobacteria;__Alphaproteobacteria;__Sphingomonadales;__Erythrobacteraceae;__Altererythrobacter	0.2	0.78	0.66	1.22	1	44.42
Eukaryota;__Cryptophyceae;__Cryptomonadales;__Geminigera;__;__	0.05	0.66	0.63	0.77	0.95	45.38
Eukaryota;__Alveolata;__Ciliophora;__Conthreep;__Phyllopharyngea;__AT1-2	0.75	0.05	0.62	0.85	0.94	46.32
Bacteria;__Proteobacteria;__Alphaproteobacteria;__Rhodobacterales;__Rhodobacteraceae;__g	0.89	0.35	0.62	1.33	0.94	47.26
Bacteria;__Proteobacteria;__Alphaproteobacteria;__SAR11_clade;Other;Other	0.61	0.07	0.61	0.78	0.92	48.19
Bacteria;__Bacteroidetes;__Flavobacteria;__Flavobacteriales;__Flavobacteriaceae;__g	0.68	0.06	0.59	0.92	0.9	49.08
Bacteria;__Candidate_division_OD1;__c;__o;__f;__g	0.56	0.02	0.57	0.82	0.87	49.95
Bacteria;__Bacteroidetes;__Flavobacteria;__Flavobacteriales;__Flavobacteriaceae;__Polaribacter	0.91	0.68	0.57	1.32	0.86	50.81
Bacteria;__Proteobacteria;__Deltaproteobacteria;__Bdellovibrionales;__Bacteriovoracaceae;__Peredibacter	0.59	0.08	0.52	1.36	0.78	51.59
Eukaryota;__Alveolata;__Ciliophora;__Conthreep;__Phyllopharyngea;__Dysteria	0.59	0.03	0.51	0.79	0.77	52.36
Eukaryota;__Cryptophyceae;__Cryptomonadales;__Rhodomonas;__;__	0.04	0.5	0.51	0.72	0.77	53.13
Bacteria;__Proteobacteria;__Alphaproteobacteria;__Rhodobacterales;__Rhodobacteraceae;__Roseobacter_clade_DC5-80-3_lineage	0.55	0.14	0.45	1.6	0.68	53.8
Bacteria;__Bacteroidetes;__Cytophagia;__Cytophagales;__Cytophagaceae;__Leadbetterella	0.18	0.5	0.43	1.39	0.66	54.46
Bacteria;__Bacteroidetes;__Sphingobacteriia;__Sphingobacteriales;__Saprospiraceae;__Lewinella	0.48	0.02	0.42	1.04	0.63	55.09
Bacteria;__Proteobacteria;__Alphaproteobacteria;__SAR11_clade;__Surface_4;__g	0.36	0.02	0.38	0.7	0.58	55.66
Bacteria;__Proteobacteria;__Gammaproteobacteria;__Enterobacteriales;__Enterobacteriaceae;Other	0.37	0.17	0.33	1.58	0.51	56.17
Bacteria;__Bacteroidetes;__Flavobacteria;__Flavobacteriales;__Flavobacteriaceae;__NS2b_marine_group	0.42	0.1	0.33	2.04	0.5	56.67
Bacteria;__Bacteroidetes;__Flavobacteria;__Flavobacteriales;__NS9_marine_group;__g	0.4	0.07	0.32	1.37	0.49	57.16
Bacteria;__Bacteroidetes;__Flavobacteria;__Flavobacteriales;__Cryomorphaceae;__Owenweeksia	0.39	0.07	0.32	1.87	0.49	57.65
Archaea;__Euryarchaeota;__Thermoplasmata;__Thermoplasmatales;__Marine_Group_II;__g	0.37	0.06	0.32	1.89	0.48	58.13
Bacteria;__Proteobacteria;__Gammaproteobacteria;__Alteromonadales;__Alteromonadaceae;__Glaciecola	0.36	0.03	0.32	1.06	0.48	58.61
Bacteria;__Bacteroidetes;__Sphingobacteriia;__Sphingobacteriales;__Saprospiraceae;__g	0.34	0.04	0.3	0.76	0.46	59.07
Bacteria;__Bacteroidetes;__Sphingobacteriia;__Sphingobacteriales;__Saprospiraceae;__Aureispira	0.32	0	0.28	0.83	0.43	59.5
Bacteria;__Bacteroidetes;__Flavobacteria;__Flavobacteriales;__Flavobacteriaceae;__NS4_marine_group	0.36	0.1	0.28	1.56	0.43	59.92
Bacteria;__Proteobacteria;__Gammaproteobacteria;Other;Other;Other	0.38	0.13	0.28	1.54	0.42	60.35
Bacteria;__Bacteroidetes;__Flavobacteria;__Flavobacteriales;__Flavobacteriaceae;__NS5_marine_group	0.32	0.07	0.28	1.12	0.42	60.77

Bacteria;__Bacteroidetes;__Sphingobacteriia;__Sphingobacteriales;__NS11-12_marine_group;__g	0.35	0.17	0.27	1.13	0.41	61.18
Bacteria;__Proteobacteria;__Gammaproteobacteria;__Oceanospirillales;__Oceanospirillaceae;Other	0.36	0.09	0.27	1.79	0.41	61.59
Bacteria;__Bacteroidetes;__Bacteroidia;__Bacteroidales;__Bacteroidaceae;__Bacteroides	0.3	0.28	0.26	1.21	0.39	61.98
Eukaryota;__Alveolata;__Ciliophora;__Spirotrichea;__Hypotrichia;__	0.31	0.03	0.25	1.37	0.39	62.37
Bacteria;__Proteobacteria;__Alphaproteobacteria;__SAR11_clade;__Chesapeake-Delaware_Bay;__g	0.13	0.23	0.25	1.12	0.38	62.75
Eukaryota;__Alveolata;__Ciliophora;__Spirotrichea;__Hypotrichia;Other	0.3	0.04	0.25	1.72	0.38	63.13
Bacteria;__Fusobacteria;__Fusobacteria;__Fusobacteriales;__Fusobacteriaceae;__Fusobacterium	0.36	0.22	0.25	1.49	0.38	63.51
Bacteria;__Proteobacteria;__Alphaproteobacteria;__SAR11_clade;__f;__g	0.24	0	0.25	0.86	0.37	63.89
Bacteria;__Proteobacteria;__Gammaproteobacteria;__Alteromonadales;__Alteromonadaceae;__SAR92_clade	0.35	0.13	0.24	1.42	0.37	64.25
Bacteria;__Proteobacteria;__Betaproteobacteria;__Burkholderiales;__Comamonadaceae;__Acidovorax	0.14	0.29	0.24	1.7	0.37	64.62
Eukaryota;__Chloroplastida;__Ulvophyceae;Other;Other;Other	0.31	0.13	0.24	1.13	0.36	64.98
Bacteria;__Proteobacteria;__Gammaproteobacteria;__Thiotrichales;__Thiotrichaceae;__Thiothrix	0.23	0	0.23	0.67	0.35	65.34
Bacteria;__Proteobacteria;__Alphaproteobacteria;__Rickettsiales;__SAR116_clade;__g	0.24	0.05	0.23	1.27	0.35	65.69
Bacteria;__Bacteroidetes;__Flavobacteria;__Flavobacteriales;__Flavobacteriaceae;__Pibocella	0.26	0.02	0.23	0.99	0.35	66.04
Bacteria;__Candidate_division_OP3;__c;__o;__f;__g	0.23	0	0.23	0.94	0.35	66.39
Eukaryota;__Alveolata;__Protalveolata;__Perkinsidae;__NA2-2D10;__	0.23	0.15	0.23	1.15	0.35	66.73
Eukaryota;__Cryptophyceae;__Cryptomonadales;__Teleaulax;__;__	0	0.23	0.23	0.66	0.35	67.08
Bacteria;__Firmicutes;__Clostridia;__Clostridiales;__Ruminococcaceae;__g	0.21	0.19	0.22	1.06	0.34	67.42
Bacteria;__Proteobacteria;__Gammaproteobacteria;__Alteromonadales;__Moritellaceae;__Moritella	0.26	0.05	0.22	1.19	0.34	67.76
Bacteria;__Proteobacteria;__Gammaproteobacteria;__Alteromonadales;__Shewanellaceae;__Shewanella	0.28	0.07	0.22	1.78	0.34	68.09
Bacteria;__Proteobacteria;__Alphaproteobacteria;__Rhodospirillales;__Rhodospirillaceae;__Defluviicoccus	0.2	0.03	0.22	0.76	0.33	68.43
Eukaryota;__Alveolata;__Ciliophora;__Spirotrichea;__Euplotia;__Aspidisca	0.26	0.02	0.22	1.61	0.33	68.76
Eukaryota;__Metazoa;__Arthropoda;__Maxillopoda;__;__	0.25	0.02	0.21	1.86	0.32	69.08
Bacteria;__Proteobacteria;__Gammaproteobacteria;__BD7-8_marine_group;__f;__g	0.29	0.29	0.21	1.6	0.32	69.4
Bacteria;__Candidate_division_OP11;__c;__o;__f;__g	0.19	0.04	0.21	0.82	0.32	69.72
Bacteria;__Proteobacteria;__Gammaproteobacteria;__Chromatiales;__Granulosicoccaceae;__Granulosicoccus	0.24	0.06	0.21	1.41	0.32	70.03
Bacteria;__Proteobacteria;__Gammaproteobacteria;__Oceanospirillales;__Oceanospirillaceae;__Neptunomonas	0.23	0.06	0.21	1.03	0.32	70.35
Eukaryota;__Alveolata;__Ciliophora;__Spirotrichea;__Oligotrichia;__uncultured	0.26	0.05	0.21	1.9	0.31	70.66
Bacteria;__Proteobacteria;__Gammaproteobacteria;__Alteromonadales;Other;Other	0.24	0.06	0.2	1.45	0.31	70.97
Bacteria;__Proteobacteria;__Alphaproteobacteria;__Rhodobacterales;__Rhodobacteraceae;__Loktanella	0.36	0.49	0.2	0.83	0.31	71.28
Bacteria;__Proteobacteria;__Alphaproteobacteria;__Rhodobacterales;__Rhodobacteraceae;__Sulfitobacter	0.22	0.03	0.2	0.78	0.31	71.58

Bacteria;__Bacteroidetes;__Flavobacteria;__Flavobacteriales;__Cryomorphaceae;__Fluviicola	0.31	0.14	0.2	1.15	0.3	71.88
Bacteria;__Proteobacteria;__Alphaproteobacteria;Other;Other;Other	0.21	0.08	0.2	0.97	0.3	72.19
Bacteria;__Candidate_division_TM7;__c;__o;__f;__g	0.19	0	0.2	0.67	0.3	72.49
Bacteria;__Proteobacteria;__Gammaproteobacteria;__Thiotrichales;__Thiotrichaceae;__Leucothrix	0.23	0.03	0.2	0.91	0.3	72.79
Bacteria;__Bacteroidetes;__Cytophagia;__Cytophagales;__Flammeovirgaceae;__Reichenbachiella	0.29	0.1	0.19	1.34	0.29	73.07
Eukaryota;__Stramenopiles;__Pirsonia;__;__;__	0.12	0.16	0.19	1.33	0.29	73.36
Eukaryota;__Rhizaria;__Cercozoa;Other;Other;Other	0.21	0	0.19	1.08	0.29	73.65
Bacteria;__Proteobacteria;__Betaproteobacteria;__Burkholderiales;__Comamonadaceae;__BAL58_marine_group	0	0.18	0.19	0.86	0.29	73.93
Bacteria;__Bacteroidetes;__Bacteroidia;__Bacteroidales;__Marinilabiaceae;__Marinifilum	0.22	0.02	0.19	0.94	0.28	74.22
Eukaryota;__Alveolata;__Ciliophora;__Spirotrichea;__Oligotrichia;__Strombidium	0.27	0.11	0.18	1.7	0.28	74.49
Bacteria;__Proteobacteria;__Gammaproteobacteria;__Order_Incertae_Sedis;__Family_Incertae_Sedis;__Arenicella	0.21	0.05	0.18	1.19	0.28	74.77
Bacteria;__Proteobacteria;__Alphaproteobacteria;__Kordiimonadales;__Kordiimonadaceae;__Kordiimonas	0.28	0.15	0.18	1.25	0.27	75.04
Eukaryota;Other;Other;Other;Other;Other	0.11	0.17	0.18	1.09	0.27	75.31
Bacteria;__Planctomycetes;__BD7-11;__o;__f;__g	0.21	0.05	0.18	1.47	0.27	75.58
Eukaryota;__Rhizaria;__Cercozoa;__Thecofilosea;Other;Other	0.2	0	0.17	0.67	0.26	75.84
Eukaryota;__Stramenopiles;__Diatomea;__Coscinodiscophytina;__Fragilariales;__	0.19	0	0.17	0.96	0.26	76.1
Bacteria;__Actinobacteria;__Acidimicrobiia;__Acidimicrobiales;__OCS155_marine_group;__g	0.16	0	0.17	0.67	0.25	76.36
Bacteria;__Proteobacteria;__Gammaproteobacteria;__Oceanospirillales;__Oceanospirillaceae;__Marinomonas	0.17	0.03	0.16	1.1	0.25	76.61
Eukaryota;__Alveolata;__Ciliophora;__Conthreep;__Phyllopharyngea;__Hartmannula	0.18	0	0.16	0.67	0.24	76.85
Bacteria;__Proteobacteria;__Betaproteobacteria;__Burkholderiales;Other;Other	0	0.16	0.16	3.42	0.24	77.08
Bacteria;__Lentisphaerae;__Lentisphaeria;__Lentisphaerales;__Lentisphaeraceae;__Lentisphaera	0.18	0.02	0.16	1	0.24	77.32
Bacteria;__Proteobacteria;__Gammaproteobacteria;__Legionellales;__Coxiellaceae;__Coxiella	0.16	0	0.16	0.96	0.24	77.56
Bacteria;__Proteobacteria;__Deltaproteobacteria;__Desulfobacterales;__Nitrospinaceae;__Nitrospina	0.16	0.02	0.16	1.19	0.24	77.79
Bacteria;__Bacteroidetes;__Flavobacteria;__Flavobacteriales;__Flavobacteriaceae;__Flavobacterium	0	0.15	0.15	0.66	0.23	78.03
Bacteria;__Proteobacteria;__Deltaproteobacteria;__Sh765B-TzT-29;__f;__g	0.15	0	0.15	0.67	0.23	78.26
Bacteria;__Proteobacteria;__Gammaproteobacteria;__Pseudomonadales;__Moraxellaceae;__Psychrobacter	0.16	0.1	0.15	1.24	0.23	78.49
Bacteria;__Proteobacteria;__Gammaproteobacteria;__Alteromonadales;__Alteromonadaceae;Other	0.25	0.1	0.15	1.53	0.23	78.72
Bacteria;__Firmicutes;__Clostridia;__Clostridiales;__Ruminococcaceae;__Faecalibacterium	0.16	0.04	0.15	0.87	0.23	78.95
Bacteria;__Proteobacteria;__Betaproteobacteria;__Nitrosomonadales;__Gallionellaceae;__Gallionella	0	0.14	0.15	1.01	0.22	79.17
Bacteria;__Bacteroidetes;__Flavobacteria;__Flavobacteriales;__Flavobacteriaceae;__Lutibacter	0.15	0.05	0.15	0.88	0.22	79.4
Bacteria;__Proteobacteria;__Deltaproteobacteria;__Bdellovibrionales;__Bacteriovoracaceae;__g	0.13	0.04	0.15	0.88	0.22	79.62

Bacteria;__Bacteroidetes;__Bacteroidia;__Bacteroidales;__Prevotellaceae;__Prevotella	0.21	0.09	0.15	1.38	0.22	79.84
Bacteria;__Bacteroidetes;__Cytophagia;__Cytophagales;__Flammeovirgaceae;__Marinoscillum	0.16	0.04	0.14	1.41	0.22	80.06
